# A macrolactonization approach to the total synthesis of the antimicrobial cyclic depsipeptide LI-F04a and diastereoisomeric analogues

**DOI:** 10.3762/bjoc.8.154

**Published:** 2012-08-21

**Authors:** James R Cochrane, Dong Hee Yoon, Christopher S P McErlean, Katrina A Jolliffe

**Affiliations:** 1 School of Chemistry, The University of Sydney, 2006, NSW, Australia; Tel: +61-2-93512297; Fax: +61-2-93513329

**Keywords:** antifungal, cyclic depsipeptide, epimerization, lipopeptide, macrolactonization, peptides

## Abstract

The cyclic peptide core of the antifungal and antibiotic cyclic depsipeptide LI-F04a was synthesised by using a modified Yamaguchi macrolactonization approach. Alternative methods of macrolactonization (e.g., Corey–Nicolaou) resulted in significant epimerization of the *C*-terminal amino acid during the cyclization reaction. The D-stereochemistry of the alanine residue in the naturally occurring cyclic peptide may be required for the antifungal activity of this natural product.

## Introduction

**Scheme 1:**
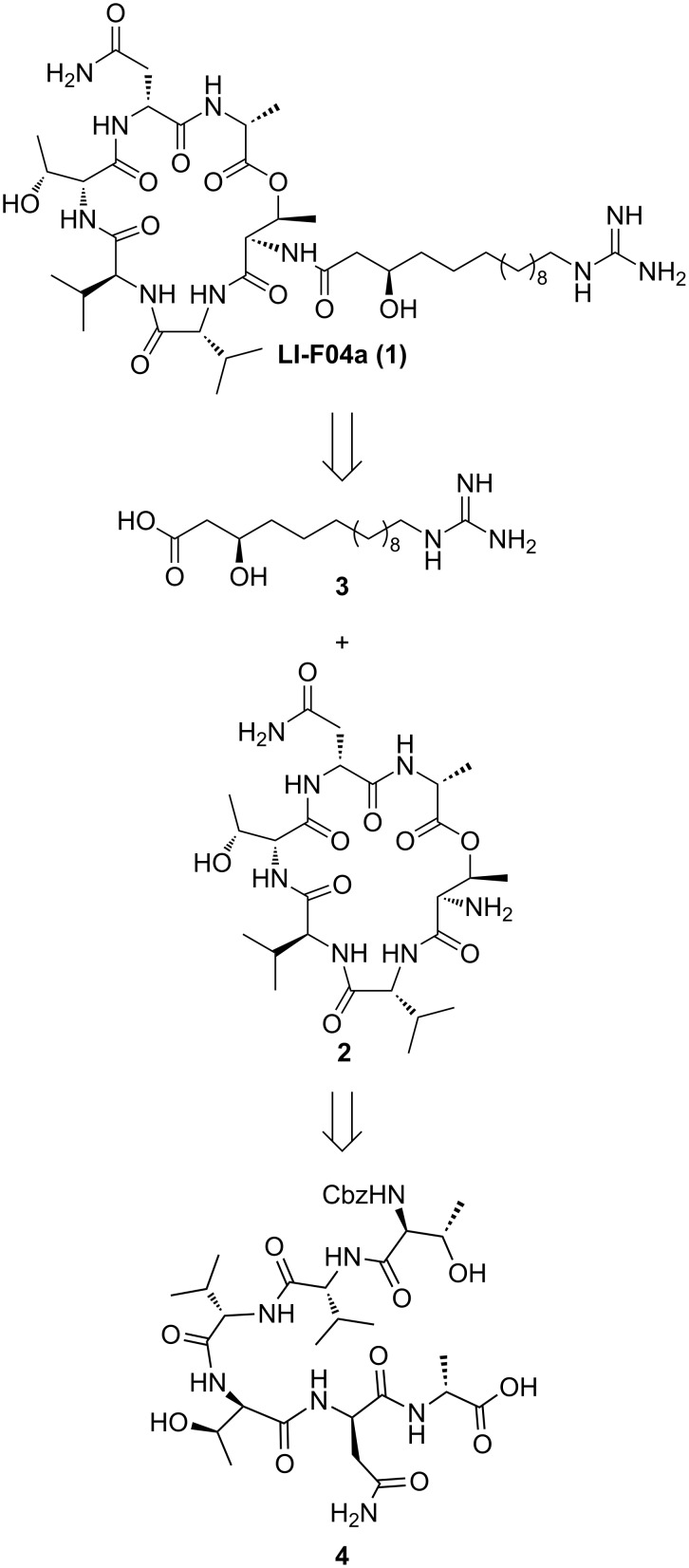
Retrosynthetic strategy.

The LI-F or fusaricidin class of cyclic depsipeptides are produced by a number of strains of *Bacillus* (*Paenebacillus*) and exhibit antifungal and antibacterial activity against a range of clinically relevant species, including *Candida albicans*, *Cryptococcus neoformans*, *Staphylococcus aureus* and *Micrococcus luteus* [1–7]. These compounds have a cyclic hexadepsipeptide core, in which three amino acids, L-Thr, D-*allo*-Thr and D-Ala are conserved throughout the series, while there are slight variations in the other three amino acids. In LI-F04a these are D-Asn, L-Val and D-Val. A unique 15-guanidino-3-hydroxypentadecanoyl (GHPD) side chain is appended to the cyclic peptide core through the nitrogen atom of the L-Thr residue. There has been recent interest in the synthesis of the LI-F family of cyclic depsipeptides due to their antifungal activity. Biosynthetic processes have been employed to this end, although these provide mixtures of depsipeptides, which makes it difficult to determine structure–activity relationships [7–8]. More recently, the solid-phase synthesis of a number of analogues of the fusaricidins has been reported. However, in all cases, the side chain 3-hydroxy group was not incorporated into the structure [9]. By total synthesis of both side-chain epimers of this structure we have recently established that the absolute configuration of this side-chain hydroxy group is (*R*) in the naturally occurring LI-F04a **1** [10]. We employed a late-stage coupling of the cyclic peptide core **2** with the GHDP side chain **3** to enable ready access to both side-chain epimers (Scheme 1). While macrocyclization to give the core **2** could be performed at any of the amide or ester bonds [10], we chose to use a macrolactonization approach to enable ready access to analogues of the LI-F04a core through straightforward Fmoc solid-phase peptide synthesis of the linear precursors. We report here our optimization of these macrolactonization conditions, together with the synthesis of several analogues of LI-F04a using this approach, and an investigation of the antifungal activity of these synthetic lipodepsipeptides.

## Results and Discussion

**Scheme 2:**
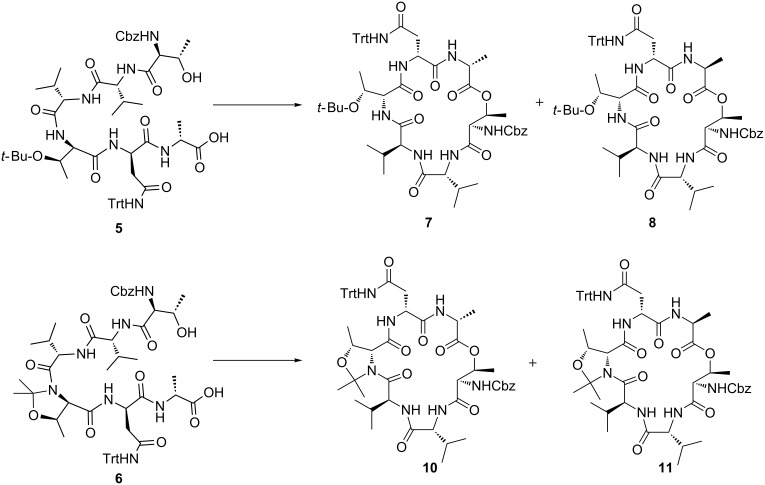
Macrolactonization reactions of seco acids **5** and **6** (for reagents and yields see Table 1 and Table 2).

The required linear precursor for the synthesis of **2** by a macrolactonization approach is **4** (Scheme 1), in which the *N-*terminal amino group of the L-Thr residue is protected while the side-chain hydroxy group is free. The Cbz group was chosen as a suitable protecting group for the *N*-terminus. The D-Asn and D-*allo*-Thr residues were the only amino acids requiring side-chain protection. Given previous reports that 2,2-dimethylated pseudoprolines (Ψ^Me,Me'^Pros) [11–13] are useful turn-inducers for improving yields of macrolactamization reactions [14–17], we chose to prepare two linear precursors, **5** and **6** (Scheme 2), in which the D-*allo*-Thr protecting group was either *tert*-butyl or Ψ^Me,Me'^Pro, respectively, to investigate whether a turn-inducer might assist the macrolactonization reaction. Linear peptides **5** and **6** were prepared by standard Fmoc solid-phase peptide synthesis protocols using PyBOP/Hünigs base as the activation reagent and 2-chlorotritylchloride resin to allow cleavage of the peptide from the solid support with side-chain protecting groups intact. In the case of **6**, the Ψ^Me,Me'^Pro was introduced by coupling the known dipeptide Fmoc-Val-D-*a*-Thr(Ψ^Me,Me'^Pro)-OH [18] into the growing peptide chain.

While there are a large number of methods available for macrolactonization reactions, those most commonly employed include the Corey–Nicolaou [19], Boden–Keck [20] and Yamaguchi [21] lactonization procedures. We initially chose to screen these three procedures for the macrolactonization of **5**. In all three cases (Table 1, entries 1–3), analysis of the crude product mixtures showed that mixtures of cyclic diastereoisomers were obtained, indicating that the *C*-terminal amino acid underwent epimerization during the macrolactonization reactions (see Supporting Information File 1 for full experimental details) [22]. However, the ratio of the two diastereoisomers differed significantly under the three sets of conditions, with the major diastereomer formed under the Yamaguchi conditions differing from the major product obtained in the other reactions.

**Table 1:** Reaction conditions for macrocyclization of **5**.

entry	reaction conditions	yield of major isomer^a^	ratio of **7**:**8**^b^

1	dithiopyridine, triphenylphosphine, MeCN, 80 °C	56%	13:87
2	DCC, DMAP, camphorsulfonic acid, CH_2_Cl_2_	14%	45:55
3	2,4,6-trichlorobenzoyl chloride, DMAP, NEt_3_, toluene, 110 °C	20%	61:39
4	2,4,6-trichlorobenzoyl chloride, DMAP, iPr_2_NEt, toluene, 80 °C	33%	69:31
5	2,4,6-trichlorobenzoyl chloride, DMAP, NEt_3_, toluene, 25 °C	33%	89:11
6	2,4,6-trichlorobenzoyl chloride, DMAP, NEt_3_, THF, 25 °C	16%	82:18
7	2,4,6-trichlorobenzoyl chloride, DMAP, NEt_3_, toluene, 25 °C, slow addition of **5**	58%	92:8^c^
8	2-methyl-6-nitrobenzoic anhydride, DMAP, NEt_3_, toluene, 25 °C, slow addition of **5**	52%	94:6
9	cyanuric chloride, MeCN, 25 °C	30%	80:20

^a^Isolated yield; ^b^as determined by integration of analytical HPLC traces of the crude reaction mixtures; ^c^average value (5–12% epimerization was observed upon repeat reactions).

**Figure 1:**
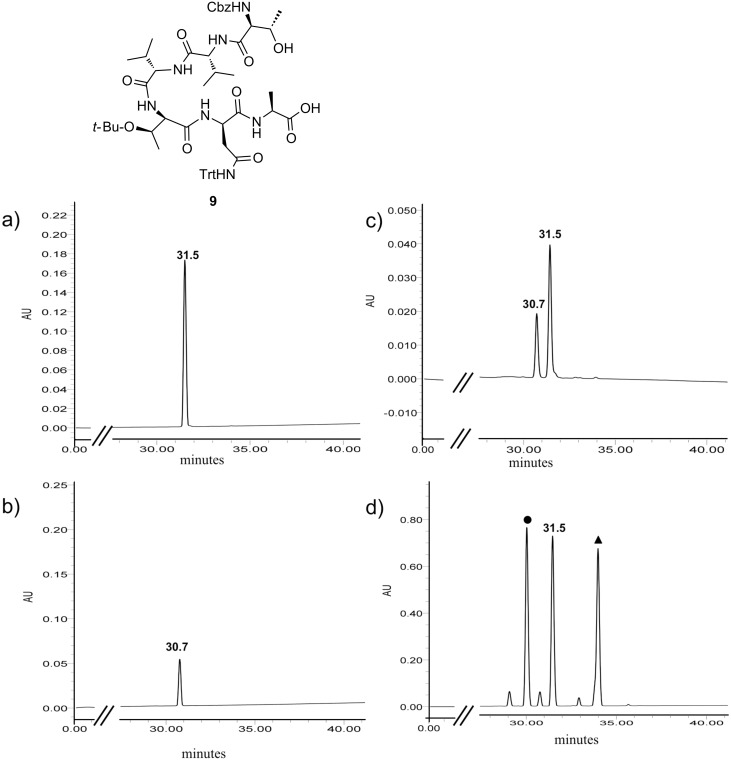
Analytical HPLC traces of linear peptides. (a) Compound **9** (retention time = 31.5 min); (b) Compound **5** (retention time = 30.7 min); (c) co-injection of mixture of **9** and **5** (2:1); (d) Crude reaction mixture after hydrolysis of major cyclic peptide from Corey–Nicolaou cyclization, indicating that the major product of hydrolysis is **9**; ▲ indicates the unreacted cyclic peptide (retention time = 34.0 min) and ● is a peak attributed to hydrolysis of both the cyclic peptide ester bond and Cbz protecting group (LCMS *m*/*z* = 902 [M + H]^+^).

In order to establish which set of conditions gave the highest ratio of the desired product **7**, the mixture of cyclic depsipeptides **7** and **8** obtained from macrolactonization under the highest yielding conditions (Corey–Nicolaou) was separated, and the major diastereomer was subjected to hydrolysis upon treatment with MeOH/H_2_O/aq NH_4_OH (4:1:1 v/v/v). Comparison of the crude peptide, so obtained, to authentic samples of both **5** and the *C*-terminal epimer **9** (which was independently prepared by solid-phase peptide synthesis), indicated that the major product obtained upon macrolactonization of **5** under the Corey–Nicolaou conditions was the undesired cyclic depsipeptide **8**, in which the *C*-terminal Ala residue had epimerized (Figure 1). Since the Yamaguchi conditions gave improved ratios of the desired/epimerized cyclic depsipeptides, subsequent optimization of the macrolactonization conditions focussed on this and related procedures (Table 1). The best yields of the desired cyclic depsipeptide **7** (58% isolated yield, with 5–12% of epimer **8** also observed) were obtained using a modification of Yonemitsu’s conditions [23] in which linear peptide **5** was added slowly to a solution of DMAP, 2,4,6-trichlorobenzoyl chloride and triethylamine in toluene at room temperature. Similar yields and low epimerization (52% isolated yield, 6% epimer observed) were obtained using 2-methyl-6-nitrobenzoic anhydride (MNBA) [24] as the activating agent in place of 2,4,6-trichlorobenzoyl chloride.

**Scheme 3:**
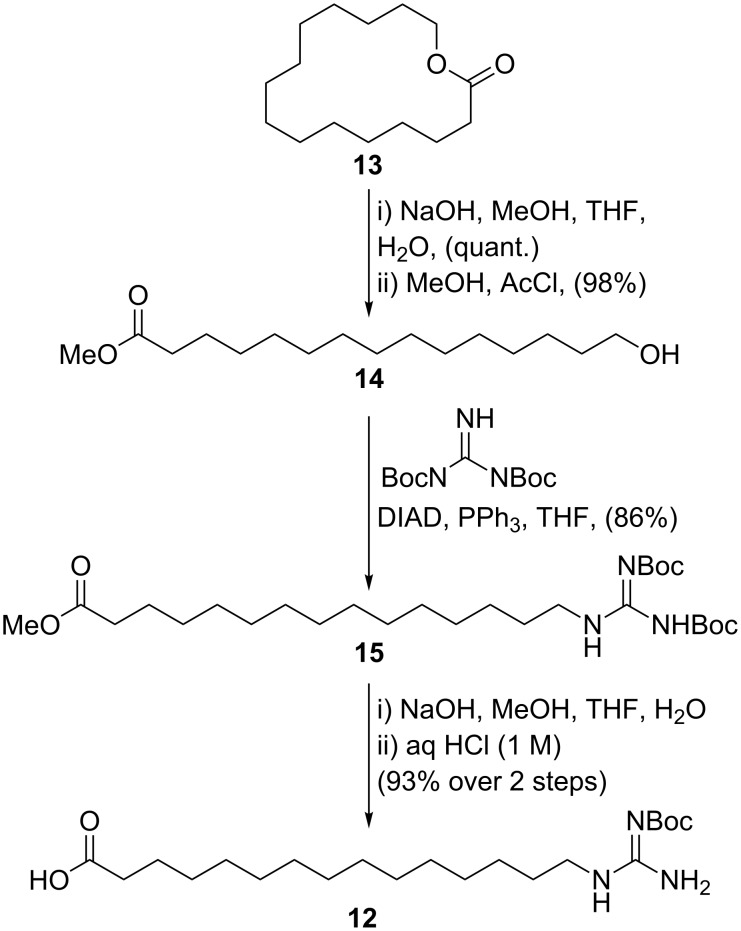
Synthesis of the dehydroxy side chain **12**.

To investigate whether the Ψ^Me,Me'^Pro would assist macrolactonization, linear precursor **6** was subject to cyclization under a range of conditions (Table 2). However, in all cases, the cyclization yields were found to be lower for the Ψ^Me,Me'^Pro-containing peptide than for the *tert*-butyl protected precursor **5**, with higher amounts of *C*-terminal epimerization also observed, except in the case of cyclization using cyanuric chloride [25]. Unfortunately, the yield could not be improved above 17% in this case, so all further reactions were performed using the Thr(O-*t*-Bu) protected cyclic peptides **7** and **8**. With cyclic peptides **7** and **8** in hand, we chose to attach the GHPD side chain to both compounds to enable the effect of peptide stereochemistry on the biological activity of the LI-F cyclic peptides to be assessed. Additionally, to investigate the effect of the 3-hydroxy group of the GHPD side chain on the biological activity of this class of cyclic peptide, we prepared the dehydroxy side-chain analogue **12** for attachment to the cyclic peptide core (Scheme 3). Notably, a previously synthesised LI-F04a analogue with a twelve-carbon side chain lacking the hydroxy group has been observed to have antimicrobial activity [9]. Thus, hydrolysis of pentadecanolide **13** [26] was followed by esterification to give the methyl ester **14** in excellent yield. Reaction of **14** with di(*tert*-butoxycarbonyl)guanidine under Mitsunobu conditions [27] proceeded smoothly to give **15** in 86% yield. Hydrolysis of the methyl ester followed by acidic work up to enable extraction of the resulting carboxylic acid gave **12**, in which one of the guanidino Boc protecting groups was also removed.

**Table 2:** Reaction conditions for macrocyclization of **6**.

entry	reaction conditions	yield of major isomer^a^	ratio of **10**:**11**^b^

1	dithiopyridine, triphenylphosphine, MeCN, 80 °C	24%	<5 to >95
2	2,4,6-trichlorobenzoyl chloride, DMAP, NEt_3_, toluene, 110 °C	19%	50:50
3	2,4,6-trichlorobenzoyl chloride, DMAP, NEt_3_, toluene, 25 °C	7%	80:20
4	2,4,6-trichlorobenzoyl chloride, DMAP, NEt_3_, toluene, 25 °C, slow addition of **5**	24%	79:21
5	cyanuric chloride, MeCN, 25 °C	17%	>95 to <5

^a^Isolated yield; ^b^as determined by integration of analytical HPLC traces of the crude reaction mixtures.

**Scheme 4:**
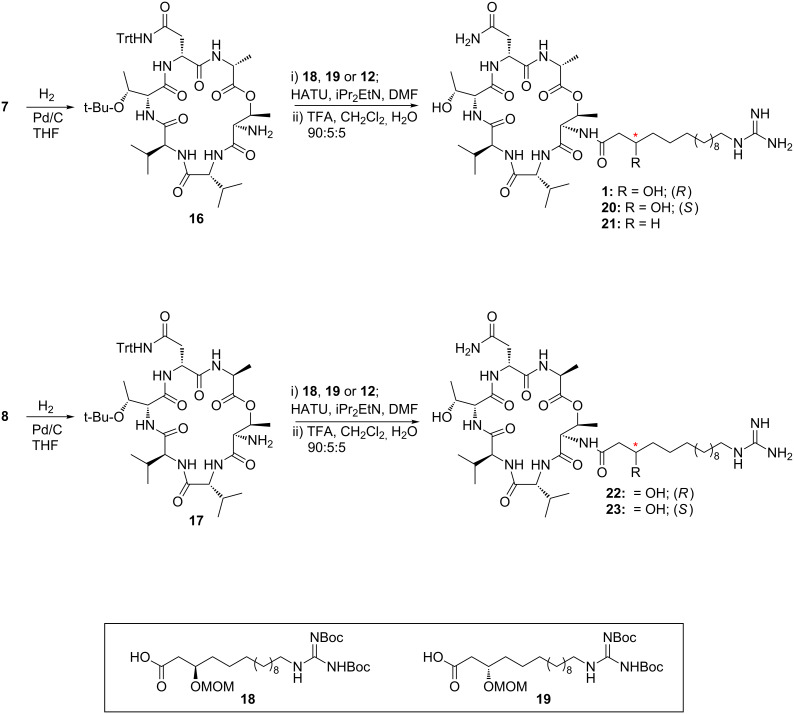
Synthesis of LI-F04a (**1**) and analogues **20**–**23**.

Hydrogenolysis of the Cbz protecting groups of **7** and **8** gave the corresponding amines **16** and **17**, respectively (Scheme 4). These were coupled with the previously synthesised side chains **18**, **19** [10] and **12**, by using HATU as the coupling agent. The resulting compounds were not isolated but immediately subjected to global deprotection upon treatment with trifluoroacetic acid/CH_2_Cl_2_/H_2_O (90:5:5 v/v/v) to give LI-F04a (**1**), side-chain epimer **20**, dehydroxy analogue **21** and the two side-chain epimers of the L-Ala derivative, **22** and **23**.

**Figure 2:**
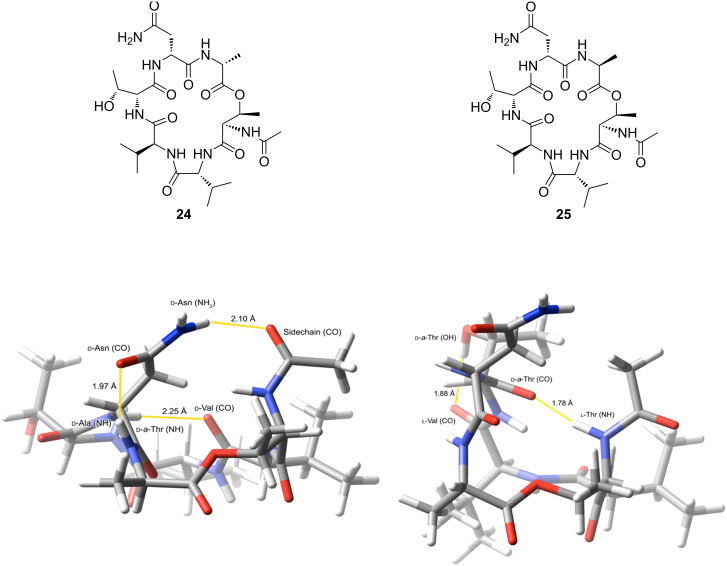
Structures and lowest-energy conformers of **24** (left) and **25** (right) obtained using Macromodel. Hydrogen bonding is highlighted in yellow.

The antifungal activity of **1** and **20**–**23** was evaluated by using a standardised serial dilution sensitivity assay [28] against reference strains of *Candida albicans*, *Cryptococcus neoformans* and *Aspergillus fumigatus* (Table 3). Synthetic LI-F04a was found to exhibit good activity against both *C. albicans* and *C. neoformans*, but only modest activity against *A. fumigatus*, consistent with the previously reported activity of the natural product [1,3,5]. The side-chain epimer **20** exhibited significantly lower activity than **1** against *C. albicans* and *C. neoformans*, indicating that the stereochemistry of the side-chain hydroxy group is important for the antifungal activity of the compounds. Removal of the hydroxy group, as in **21**, resulted in a further small decrease in activity against these species. Notably, compounds **22** and **23**, prepared from the *C*-terminal-epimerised cyclic peptide did not exhibit antifungal activity against any of the species tested. This suggests that the conformation of the cyclic peptide core is important in determining the antifungal activity of these compounds, since inversion of the stereocentre of one of the amino acids in the macrocycle is expected to result in a significantly different peptide conformation [29]. Modelled structures of the side-chain-acylated cyclic peptides **24** and **25** obtained by using Monte Carlo conformational searches in Macromodel [30] suggest that these cyclic peptides adopt significantly different conformations with different arrangements of hydrogen bonds (Figure 2). The temperature dependence of the chemical shifts of the signals attributable to the amide NHs of **1** in *d*_6_-DMSO was determined experimentally and confirmed the involvement of the D-Ala and D-Asn amide protons in hydrogen bonds [31] as suggested by the modelling studies of the acetamide analogue (see Supporting Information File 1 for details). This indicates that these hydrogen bonds may be important in locking the cyclic depsipeptide into a biologically active conformation.

**Table 3:** Antifungal activity.

compound	*C. albicans* ATCC 10231 (MIC µM)^a^	*C. neoformans* ATCC 90112 (MIC µM)^a^	*A. fumigatus* ATCC 204305 (MIC µM)^a^

**1**	5.5	2.8	44
**20**	22	11	22
**21**	44	22	22
**22**	>88	>88	>88
**23**	>88	>88	>88

^a^MICs for amphotericin B: *C. albicans* 0.35 µM; *C. neoformans* 0.35 µM; *A. fumigatus* 0.70 µM.

## Conclusion

In summary, macrolactonization to form the cyclic depsipeptide core of LI-F04a was achieved in good yields by using either modified Yonemitsu conditions or similar conditions, in which the 2,4,6-trichlorobenzoyl chloride activating agent was replaced with MNBA. Slow addition of the linear seco-acid to the activating agents was found to be the key factor to minimizing epimerization of the *C-*terminal amino acid during the macrolactonization reaction. Synthetic LI-F04a was found to exhibit similar antifungal activity to that reported for the naturally occurring material. The antifungal activity of **1** was reduced upon either inversion of the stereochemistry, or deletion of the side-chain hydroxy group. Inversion of the D-Ala residue in the cyclic depsipeptide core resulted in complete loss of antifungal activity, indicating that the cyclic peptide conformation may be important in the biological activity of this class of cyclic lipodepsipeptide.

## Supporting Information

File 1 Experimental details for all new compounds.

File 2 ^1^H, ^13^C and 2D NMR data for all new compounds.
