# Supplementary material for: A macrolactonization approach to the total synthesis of the antimicrobial cyclic depsipeptide LI-F04a and diastereoisomeric analogues
Source: Beilstein J Org Chem. 2012 Aug 21;8:1344–51. doi: 10.3762/bjoc.8.154 (PMC3458759; doi:10.3762/bjoc.8.154)
Supplement: File 1 — Experimental details for all new compounds. [file Beilstein_J_Org_Chem-08-1344-s001.pdf]

Supporting Information File 1
for

A macrolactonization approach to the total synthesis of the antimicrobial cyclic depsipeptide LI-F04a and diastereoisomeric analogues

James R. Cochrane, Dong Hee Yoon, Christopher S. P. McErlean and Katrina A. Jolliffe*

Address: School of Chemistry, The University of Sydney, 2006, NSW, Australia;

Tel: +61-2-93512297; Fax: +61-2-93513329

Email: Katrina A. Jolliffe - kate.jolliffe@sydney.edu.au

* Corresponding author

Experimental details for all new compounds

	Page
General information	S2
Experimental procedures for new compounds and analytical HPLC traces of cyclisation reactions	S3
Temperature dependence of amide proton chemical shifts of 1	S19
References	S20

General information

Melting points were obtained using a Gallenkamp melting point apparatus and are uncorrected. ^{1}H Nuclear magnetic resonance spectra were recorded on a Bruker Avance DPX 200 at a frequency of 200.13 MHz or a Bruker Avance DPX 400 at a frequency of 400.13 MHz. The spectra are reported as parts per million (ppm) downfield shift, by using the residual solvent peak as a reference. The data is reported as chemical shift (δ), relative integral, multiplicity (s = singlet, br = broad, d = doublet, t = triplet, q = quartet, dd = doublet of doublets, dt = doublet of triplets, dq doublet of quartets, m = multiplet) and coupling constant (*J* in hertz).^{13}C Nuclear magnetic resonance spectra were recorded by using a Bruker Avance DPX 400 at a frequency of 100.61 MHz and are reported as parts per million (ppm) downfield shift with the solvent peak as a reference. Low-resolution mass spectra were recorded on a Finnigan LCQ ion trap mass spectrometer (ESI). High-resolution mass spectra were recorded on a Bruker DALTONICS Apex Qe 7.0T Fourier transform ion cyclotron resonance mass spectrometer (FTICR) with an analytical electrospray source. Infrared spectra were obtained with an FTIR fitted with a ZnSe ATR accessory. Analytical, preparative and semipreparative reverse phase HPLC (RP-HPLC) was performed by using a Waters 600 multisolvent delivery system and Waters 500 pump with a 2998 photodiode array detector or a Waters 490E programmable wavelength detector operating at 254 and 214 nm. Analytical HPLC employed a Waters Sunfire C18 column (2.1 × 150 mm column, 5 μm particle size, flow rate of 0.8 mL min^{-1}). Preparative RP-HPLC employed a Waters Sunfire Prep C18 OBD column (19 × 150 mm, 5 μm particle size, flow rate 7 mL min^{-1}). Semipreparative RP-HPLC employed a Waters Sunfire C18 column (10 × 250 mm, 5 μm particle size, flow rate 4 mL min^{-1}). The mobile phase consisted of eluents A (0.1% TFA in water) and B (0.1% TFA in acetonitrile) for all HPLC runs a gradient of 30–100% B over 40 min was used, unless otherwise stated. Chiral HPLC was performed by using a Waters 510 pump with a Waters 2487 photodiode array detector and a Waters 410 differential reflectometer. A DIACEL chiralcell OD-H column (0.46 × 25 cm column, flow rate 1 mL/min) was employed, using a mobile phase consisting of 95:5 (v/v) *n*-hexane/isopropanol. The results were analysed on Waters System's Empower software. Elemental analysis was performed at Campbell Microanalytical Laboratory, Department of Chemistry, University of Otago. Optical rotations were obtained by using a Perkin Elmer model 341 polarimeter at 20 °C with the indicated spectroscopic grade solvents. Analytical thin layer chromatography (TLC) was performed using preconditioned plates (Merck Kieselgel 60 F254) and spots were visualized under UV, or stained with potassium permanganate or ceric ammonium molybdate solution. Flash column chromatography was carried out by using Merck Kieselgel 60 silica gel (SiO_2, 0.04–0.065 μm) with the indicated solvents. Ratios of the solvents used for TLC or column chromatography are expressed in v/v as specified. Most reagents were commercially available reagent-grade chemicals

and were used without further purification. CH_2Cl_2, methanol and triethylamine were distilled from calcium hydride; THF was distilled from sodium and benzophenone before use. DMF was obtained as peptide synthesis grade from Auspep and stored over 4 Å molecular sieves.

General methods for SPPS; optimised synthetic procedures and characterisation data for compounds **5**, **7**, **16**, **1** and **20** have been previously reported [1].

Experimental procedures

Cbz-L-Thr-D-Val-L-Val-D-*allo*-Thr(ΨPro)-D-Asn(Trt)-D-Ala-OH (6)

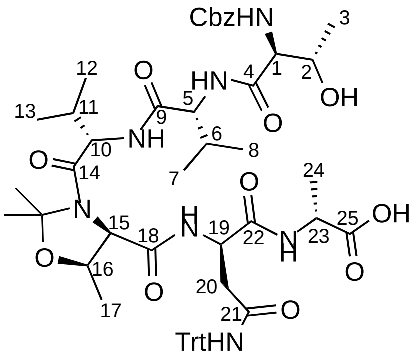

The hexapeptide **6** was synthesized following the general method for solid-phase peptide synthesis on a 0.30 mmol scale. The peptide was cleaved from the resin by agitating with 4:1 v/v CH_2Cl_2/1,1,1,3,3,3-hexafluoro-2-propanol (3 × 5 mL) for 20 min. The solvent was removed under reduced pressure and the residue azeotropically distilled with toluene (3 × 30 mL). The residue was then purified by flash chromatography eluting with 4:1 chloroform/methanol. This afforded **6** as an amorphous colourless solid (0.23 g, 75%, based on estimated loading of D-Ala resin of 0.8 mmol/g); **mp** 155–160 °C; $[\alpha]^{20}_D$ +30 (*c* 0.73, MeOH); ^{1}**H NMR** (400 MHz, CD_3CN) δ 7.63 (1 H, br s, C21NH), 7.54 (1 H, d, *J* = 8.4 Hz, C19NH), 7.38–7.22 (22 H, m, ArH, C5NH, C23NH), 7.01 (1 H, d, 7.8 Hz, C10NH), 6.18 (1 H, d, *J* = 6.4 Hz, C1NH), 5.15 (1 H, d, *J* = 12.5 Hz, OCHHAr), 5.09 (1 H, d, *J* = 12.5 Hz, OCHHAr), 4.86 (1 H, d, *J* = 6.0 Hz, H15), 4.64 (1 H, t, *J* = 6.1 Hz, H19), 4.35 (1 H, m, H16), 4.28 (1 H, m, H23), 4.15–4.11 (2 H, m, H2, H5), 4.06 (1 H, dd, *J* = 6.4, 3.9 Hz, H1), 3.97 (1 H, m, H10), 2.85 (1 H, dd, *J* = 16.0, 6.6 Hz, H20a), 2.80 (1 H, dd, *J* = 16.0, 5.6 Hz, H20b), 2.41 (1 H, br s, C2OH), 2.15 (1 H, m, H6), 1.96 (1 H, m, H11), 1.66 (3 H, s, $OC(CH_3)_2N$), 1.47 (3 H, s, $OC(CH_3)_2N$), 1.28 (3 H, d, *J* = 7.1 Hz, H24), 1.21 (3 H, d, *J* = 6.0 Hz, H17), 1.15 (3 H, d, *J* = 6.1 Hz, H3), 0.91 (3 H, d, *J* = 6.8 Hz, H7), 0.85 (3 H, d, *J* = 6.8 Hz, H8), 0.81 (3 H, d, *J* = 6.4 Hz, H12), 0.71 (3 H, d, *J* = 6.8 Hz, H13); ^{13}**C NMR** (100 MHz, CD_3CN) δ 174.2 (C=O, C25), 172.6 (C=O, C9), 172.3 (C=O, C4), 171.5 (C=O, C22), 171.2 (C=O, C21), 170.0 (C=O, C18), 169.9 (C=O, C14), 157.5 (C=O, $NHCOOCH_2Ar$), 145.6 (Cq, Ar), 138.0 (Cq, Ar), 129.7 (CH, Ar), 129.5

(CH, Ar), 129.0 (CH, Ar), 128.7 (CH, Ar), 127.8 (CH, Ar), 96.4 (Cq, O<u>C</u>(CH_3)$_2$N), 73.6 (CH, C16), 71.3 (Cq, Ar), 68.3 (CH, C2), 67.4 (CH_2, O<u>C</u>H_2Ar), 65.1 (CH, C15), 61.6 (CH, C1), 59.8 (CH, C5), 59.0 (CH, C10), 50.3 (CH, C19), 49.3 (CH, C23), 39.5 (CH_2, C20), 31.3 (CH, C6), 30.9 (CH, C11), 25.6 (CH_3, OC(<u>C</u>H_3)$_2$N), 23.6 (CH_3, OC(<u>C</u>H_3)$_2$N), 21.1 (CH_3, C8), 20.1 (CH_3, C7), 19.9 (CH_3, C3), 19.2 (CH_3, C13), 18.5 (CH_3,C12), 17.9 (CH_3, C24), 15.8 (CH_3, C17); **MS** (ESI) *m/z* 1042 [(M+Na)$^+$, 100%] 1043 (80), 1045 (35); **HRMS** (ESI, MNa^+) Calcd for $C_{55}H_{69}N_7O_{12}Na$ 1042.4897 found 1042.4880; **IR** ($CHCl_3$): 3300, 2967, 1652, 1513, 1222 cm^{-1}.

Cbz-L-Thr-D-Val-L-Val-D-*allo*-Thr(*tert*-butyl)-D-Asn(Trt)-L-Ala-OH (9)

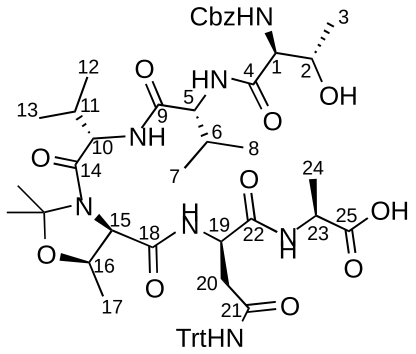

The hexapeptide **9** was synthesized by following the general method for solid-phase peptide synthesis on 0.20 mmol scale. The peptide was cleaved from the resin by agitating with (4:1 CH_2Cl_2/1,1,1,3,3,3-hexafluoro-2-propanol) (3 × 5 mL) for 20 min. The solvent was removed under reduced pressure and the residue azeotropically distilled with toluene (3 × 30 mL). The residue was then purified by preparative RP-HPLC under the standard conditions. This afforded **9** as an amorphous colourless solid (0.19 g, 90%, based on estimated loading of L-Ala resin of 0.8 mmol/g); **[α]$^{20}_D$** +8.4 (*c* 0.73, MeOH); **^{1}H NMR** (400 MHz, CD_3OD) δ 8.52 (1 H, s, C21NH), 8.18 (1 H, d, *J* = 7.3 Hz, C15NH), 8.11 (1 H, d, *J* = 7.6 Hz, C19NH), 7.96 (1 H, d, *J* = 7.3 Hz, C10NH), 7.89 (1 H, d, *J* =7.8 Hz, C23NH), 7.83, (1 H, d, *J* = 7.8 Hz, C5NH), 7.34–7.17 (20 H, m, ArH), 7.03 (1 H, m, C1NH), 5.10 (1 H, d, *J* = 12.5 Hz, OC<u>H</u>HAr), 5.03 (1 H, d, *J* = 12.5 Hz, OCH<u>H</u>Ar), 4.72 (1 H, t, *J* = 6.6 Hz, H19), 4.47 (1 H, m, H15), 4.42 (1 H, m, H23), 4.26 (1 H, d, *J* = 6.4 Hz, H5), 4.20 (1 H, m, H16), 4.13 (3 H, m, H1, H2, H10), 2.98 (1 H, dd, *J* = 6.0, 15.6 Hz, H20a), 2.77 (1 H, dd, *J* =8.3, 15.6 Hz, H20b), 2.17 (1 H, m, H6), 2.07 (1H, m, H11), 1.37 (3 H, d, *J* = 7.3 Hz, H24), 1.17 (12 H, m, H3, *tert*-butyl), 1.05 (3 H, d, *J* = 6.4 Hz, H17), 0.98 (6 H, d, *J* = 6.4 Hz, H7, H8), 0.94 (3 H, d, *J* = 6.6 Hz, H13), 0.90 (3 H, d, *J* = 6.6 Hz, H12), (OHs not observed); **^{13}C NMR** (100 MHz, CD_3OD) δ 175.6 (C=O, C25), 174.9 (C=O, C14), 174.0 (C=O, C9), 173.5 (C=O, C4), 172.5 (C=O, C18), 172.2 (C=O, C22), 171.5 (C=O, C21), 158.5 (C=O, NH<u>C</u>OOCH$_2$Ar), 145.9 (Cq, Ar), 138.1 (Cq, Ar), 130.1 (CH, Ar), 129.5 (CH, Ar), 129.1 (CH, Ar), 129.0 (CH, Ar), 128.7 (CH, Ar), 127.8 (CH,

Ar), 75.6 (Cq, *tert*-butyl), 71.8 (Cq, Ar), 68.6 (CH, C2), 67.8 (CH_2, OC̲H_2Ar), 67.4 (CH, C16), 62.2 (CH, C1), 61.0 (CH, C5), 60.9 (CH, C15), 60.1 (CH, C10), 51.7 (CH, C19), 49.2 (CH, C23), 39.0 (CH_2, C20), 31.5 (CH, C11), 31.0 (CH, C6), 28.5 (3 C, CH_3, *tert*-butyl), 20.0 (CH_3, C3), 19.9 (CH_3, C8), 19.8 (CH_3, C12), 19.4 (CH_3, C7), 18.9 (CH_3, C17), 18.5 (CH_3, C13), 18.0 (CH_3, C24); **MS** (ESI) *m/z* 1059 [$(M+Na)^+$, 100%]; **HRMS** (ESI, MNa^+) Calcd for $C_{56}H_{74}N_7O_{12}$ 1036.5390 found 1036.5410.

Cyclic depsipeptide (7)

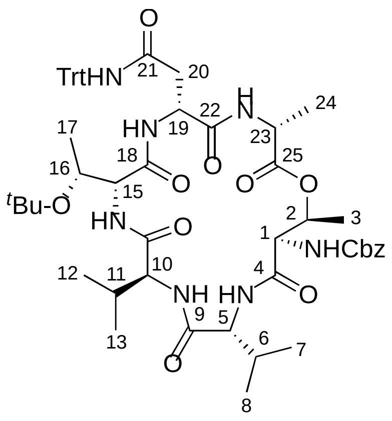

Optimised conditions

The seco acid **5** (0.18 g, 0.17 mmol) was dissolved in dry toluene (30 mL) and added dropwise over 16 h to a solution of triethylamine (34 μL, 0.25 mmol), 2,4,6 trichlorobenzoyl chloride (40 μL, 0.25 mmol) and DMAP (0.82 g, 0.67 mmol) in toluene (140 mL, final concentration of acid 0.001 M). After the addition, the solution was left under stirring for 72 h. The toluene was then removed under reduced pressure. The residue was redissolved in methanol and analysed by analytical HPLC (50% to 100% B over 60 min) before the crude product was purified by flash chromatography (9:1 $CHCl_3$/MeOH). This afforded **7** as a colourless solid (0.10 g, 58%, 12% epimerisation of crude material when processed at 214 nm, 8% epimerisation using max plot); **mp** 260 °C (decomp.); $[\alpha]^{20}_D$ +13 (*c* 0.1, MeOH); ^{1}**H NMR** (400 MHz, DMSO-d_6, 60 °C) δ 8.41 (1 H, br s, C1NH), 8.16 (2 H, m, C19NH, C10NH), 8.05 (1 H, d, *J* = 7.5 Hz, C15NH). 7.33–7.15 (21 H, m, ArH, C21NH), 7.07 (1 H, br s, C23NH), 6.95 (1 H, d, *J* = 9.0 Hz, C5NH), 5.28 (1 H, dq, *J* = 6.4, 2.0 Hz, H2), 5.1 (1 H, dd, *J* = 12.1 Hz, OCH̲HAr), 5.04 (1 H, d, *J* = 12.4 Hz, OCHH̲Ar), 4.48 (1 H, dd, *J* = 9.0, 6.8 Hz, H5), 4.31 (2 H, m, H19, H10), 4.22–4.13 (3 H, m, H23, H1, H15), 3.98 (1 H, q, *J* = 6.1 Hz, H16), 3.08 (1 H, dd, *J* = 15.4, 6.6 Hz, H20a), 2.61 (1 H, dd, *J* = 15.4, 6.6 Hz, H20b), 2.00 (1 H, m, H11), 1.83 (1 H, m, H6), 1.26 (3 H, br s, H24), 1.20 (3 H, m, H3), 1.13 (9 H, s, *tert*-butyl), 1.06 (3

H, d, J = 6.3 Hz, H17), 0.89 (6 H, m, H12, H13), 0.81 (3 H, d, J = 6.8 Hz, H8), 0.73 (3 H, d, J = 6.8 Hz, H7); ^{13}**C NMR** (100 MHz, DMSO-d_6, 60 °C) δ 172.1 (C=O, C9), 170.3 (C=O, C18), 170.1 (C=O, C25), 169.8 (C=O, C14), 169.7 (C=O, C22), 169.2 (C=O, C21), 168.1 (C=O, C4), 156.0 (C=O, NH$\underline{C}$OOCH$_2$Ar), 144.7 (Cq, Ar), 136.5 (Cq, Ar), 128.4 (CH, Ar), 128.0 (CH, Ar), 127.5 (CH, Ar), 127.2 (CH, Ar), 127.1 (CH, Ar), 126.0 (CH, Ar), 73.3 (Cq, *tert*-butyl), 69.9 (CH, C2), 69.4 (Cq, Ar), 65.7 (CH$_2$, $\underline{C}$H$_2$Ar), 59.6, (CH, C10), 59.5 (CH, C1), 59.3 (CH, C16), 57.5 (CH, C5), 56.4 (CH, C15), 50.1 (CH, C19), 47.7 (CH, C23), 37.7, (CH$_2$, C20), 31.4 (CH, C6), 30.0 (CH, C11), 27.9 (3 C, *tert*-butyl), 18.9 (CH$_3$, C13), 18.6 (CH$_3$, C12), 18.5 (CH$_3$, C8), 17.7 (CH$_3$, C17), 17.4 (CH$_3$, C7), 17.1 (CH$_3$, C24), 16.2 (CH$_3$, C3); **IR** (CHCl$_3$): 3041, 3024, 2401, 1664, 1529, 1514 cm^{-1}; **MS** (ESI) m/z 1018 [(M+H)$^+$, 100%], 1050 (50), 1040 (30); **HRMS** (ESI, MH$^+$) Calcd for C$_{56}$H$_{72}$N$_7$O$_{11}$, 1018.5284; found, 108.5306.

Analytical HPLC trace of crude reaction mixture: expansion 20-40 min

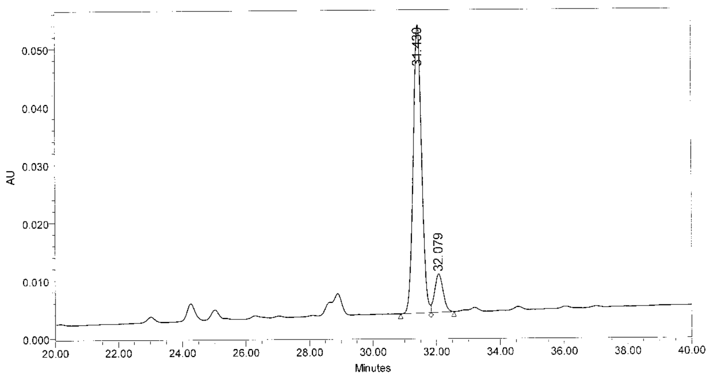

	RT	Area	% Area	Height
1	31.430	916369	88.27	49787
2	32.079	121725	11.73	6628

Analytical HPLC trace after purification by preparative TLC:

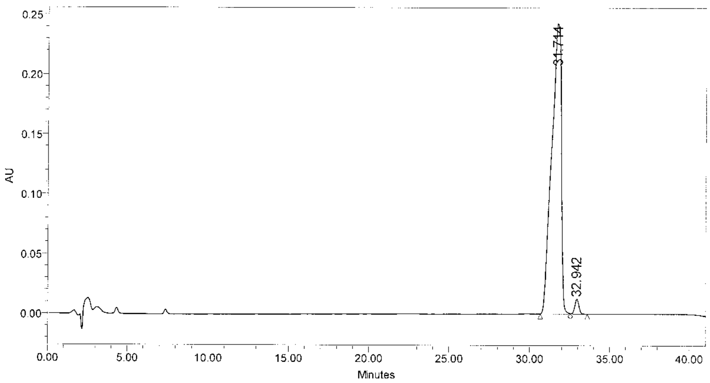

	RT	Area	% Area	Height
1	31.714	10110734	97.85	242622
2	32.942	222565	2.15	12465

Cyclic depsipeptide (8)

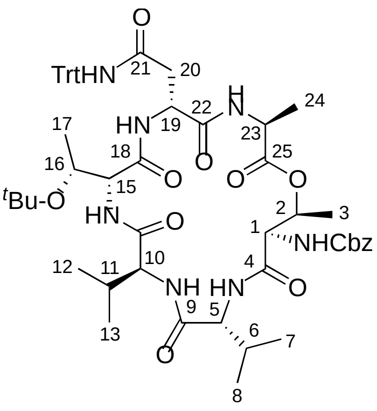

5 (0.19 g, 0.18 mmol), triphenylphosphine (71 mg, 0.27 mmol) and dithiopyridine (60 mg, 0.27 mmol) were dissolved in THF (3.6 mL) under an atmosphere of argon. This solution was stirred for 16 h, before being diluted to 10 mL with acetonitrile and added over 16 h to acetonitrile (90 mL) under reflux. After the addition the solution was heated under reflux for 48 h. The solvent was then removed under reduced pressure and the residue was purified by using RP-HPLC under the standard conditions, followed by Prep TLC (9:1 methanol/chloroform) afforded the title product as a colourless solid (0.10 g, 56%); **mp** 250 °C (decomp.); **$[\alpha]^{20}_{D}$** +20 (*c* 0.1, MeOH); **^{1}H NMR** (400 MHz, CD_3OD) δ 7.24 (20 H, s, ArH), 5.26 (1 H, m, H2), 4.93 (2 H, m, OC<u>H</u>$_2$Ar), 4.69 (1 H, m, H19), 4.46 (1 H, d, *J* = 5.9 Hz, H5), 4.26 (3 H, m, H15, H16, H23), 4.18 (1 H, m, H1), 3.96 (1 H, d, *J* = 8.8 Hz, H10), 2.98 (2 H, m, H20), 1.96 (1 H, m, H11), 1.86 (1 H, m, H6), 1.35 (3 H, d, *J* = 7.3 Hz, H24), 1.28 (3 H, m, H3), 1.20 (9H, s, *tert*-butyl), 1.11 (3 H, d, *J* = 6.6 Hz, H17), 1.09 (3 H, d, *J* = 6.6 Hz, H13), 1.02 (3 H, d, *J* = 6.6 Hz, H12), 0.84 (3 H, d, *J* = 6.6 Hz, H8), 0.73 (3 H, d, *J* = 6.6 Hz, H7); **^{13}C NMR** (100 MHz, CD_3OD) δ 176.7 (C=O, C14), 173.6 (C=O, C25), 173.6 (C=O, C22), 172.4 (C=O, C9), 172.4 (C=O, C21), 171.1 (C=O, C18), 170.4 (C=O, C4), 158.6 (C=O, NH<u>C</u>OOCH$_2$Ar), 145.9 (Cq, Ar), 137.7 (Cq, Ar), 130.1 (CH, Ar), 129.6 (CH, Ar), 129.1 (CH, Ar), 128.7 (CH, Ar), 127.9 (CH, Ar), 75.7 (Cq, *tert*-butyl), 72.8 (CH, C2), 72.0 (Cq, Ar), 68.2 (CH, C16), 66.5 (CH_2, <u>C</u>H$_2$Ar), 62.3 (CH, C1), 62.0 (CH, C10), 61.5 (CH, C5), 57.9 (CH, C15), 53.2 (CH, C19), 50.2 (CH, C23), 40.2 (CH_2, C20), 34.2 (CH, C6), 30.6 (CH, C11), 28.4 (3 C, CH_3, *tert*-butyl), 20.4 (CH_3, C13), 19.7 (CH_3, C12), 19.5 (CH_3, C8), 18.2 (CH_3, C17), 16.8 (CH_3, C7), 16.6 (CH_3, C24), 16.4 (CH_3, C3); **IR** ($CHCl_3$): 3041, 3024, 2401, 1664, 1529, 1514 cm^{-1}; MS (ESI) *m/z* 1018 [$(M+H)^+$, 100%], 1050 (50), 1040 (30); **HRMS** (ESI, MNa^+) Calcd for $C_{56}H_{71}N_7O_{11}Na$ 1040.5104 found 1040.5120.

Analytical HPLC trace of crude reaction mixture:

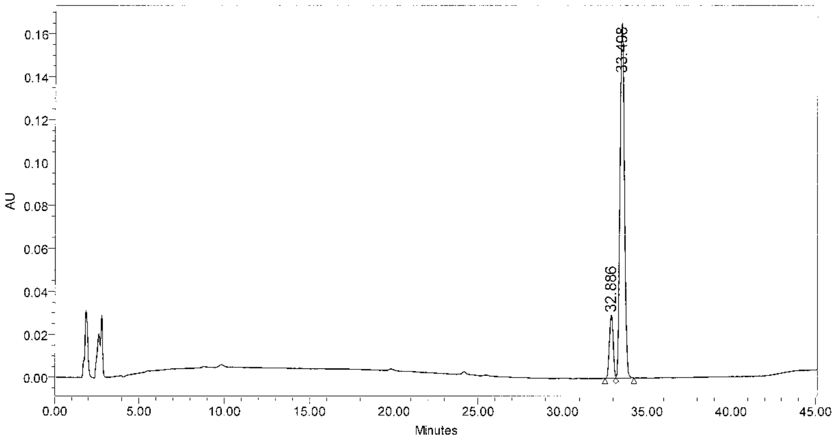

	RT	Area	% Area	Height
1	32.886	461735	13.16	29431
2	33.498	3047448	86.84	165115

Cyclic depsipeptide (11)

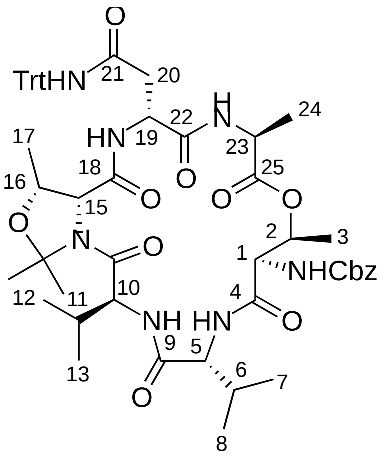

Compound **6** (86 mg, 0.084 mmol), triphenylphosphine (33 mg, 0.15 mmol) and dithiopyridine (33 mg, 0.15 mmol) were dissolved in toluene (3.5 mL) under an atmosphere of argon. This solution was stirred for 16 h, before being diluted to 10 mL and added over 16 h to toluene (20 mL) under reflux. After the addition, the solution was left under reflux for 48 h. The toluene was then removed under reduced pressure and the residue purified by RP-HPLC under the standard conditions. This afforded the title product **11** as a colourless solid (20 mg, 24%); **mp** 260 °C (decomp.); $[\alpha]^{20}_{D}$ +14 (*c* 0.05, MeOH), **^{1}H NMR** (400 MHz, CD_3OD) δ 8.27 (1 H, s br, C21NH), 7.93 (1 H, s br, C23NH), 7.61 (1 H, s br, C5NH), 7.37–7.18 (20 H, m, ArH), 5.20 (1 H, m, H2), 4.95 (2 H, s, $OC\underline{H}_2Ar$), 4.67 (1 H, m, H19), 4.55 (2 H, m, H16, H5), 4.42 (1 H, d, *J* = 6.6 Hz, H15),

4.23 (2 H, m, H1, H10), 4.13 (m, 1 H, H23), 3.05 (2 H, m, H20), 2.06 (2 H, m, H6, H11), 1.82 (3 H, s, $OC(C\underline{H}_3)_2N$), 1.67 (3 H, s, $OC(C\underline{H}_3)_2N$), 1.35 (3 H, d, *J* = 7.1 Hz, H24), 1.32 (6 H, m, H3, H17), 1.04 (6 H, m, H12, H13), 0.88 (3 H, d, *J* = 6.8 Hz, H8), 0.68 (3 H, s br, H7), (3 NH signals not observed); **^{13}C NMR** (100 MHz, CD_3OD) δ 173.4 (C=O, C22), 173.2 (C=O, C9), 172.7 (C=O, C25), 171.6 (C=O, C14), 171.3 (C=O, C4), 170.6 (C=O, C18), 170.4 (C=O, C21), 158.4 (C=O, $NH\underline{C}OOCH_2Ar$), 146.0 (Cq, Ar), 137.9 (Cq, Ar), 130.1 (CH, Ar), 129.6 (CH, Ar), 129.5 (CH, Ar), 129.1 (CH, Ar), 128.8 (CH, Ar), 127.8 (CH, Ar), 95.8 ($O\underline{C}(CH_3)_2N$), 72.3 (CH, C2), 72.0 (CH, C16), 71.9 (Cq, Ar), 67.9 (CH_2, $O\underline{C}H_2Ar$), 66.8 (CH, C1), 60.5 (CH, C15), 58.0 (CH, C10), 58.0 (CH, C5), 52.6 (CH, C19), 50.6 (CH, C23), 40.4 (CH_2, C20), 32.5 (CH, C6), 30.8 (CH, C11), 29.5 (CH_3, $OC(\underline{C}H_3)_2N$), 28.0 (CH_3, $OC(\underline{C}H_3)_2N$), 20.0 (CH_3, C13), 19.9 (CH_3, C12), 18.2 (CH_3, C8), 18.0 (CH_3, C7), 16.7 (CH_3, C17), 15.9 (CH_3, C24), 15.6 (CH_3, C3); **MS** (ESI) *m/z* 1024 [$(M+Na)^+$, 100%]; **HRMS** (ESI, MNa^+) Calcd for $C_{55}H_{69}N_7O_{12}Na$ 1024.4791 found 1024.4785; **IR** (CH_2Cl_2): 3043, 3032, 3024, 3016, 3009, 2970.17, 2399, 2392, 1728, 1713, 1670, 1643, 1632, 1528, 1520 cm^{-1}.

Analytical HPLC trace of crude reaction mixture

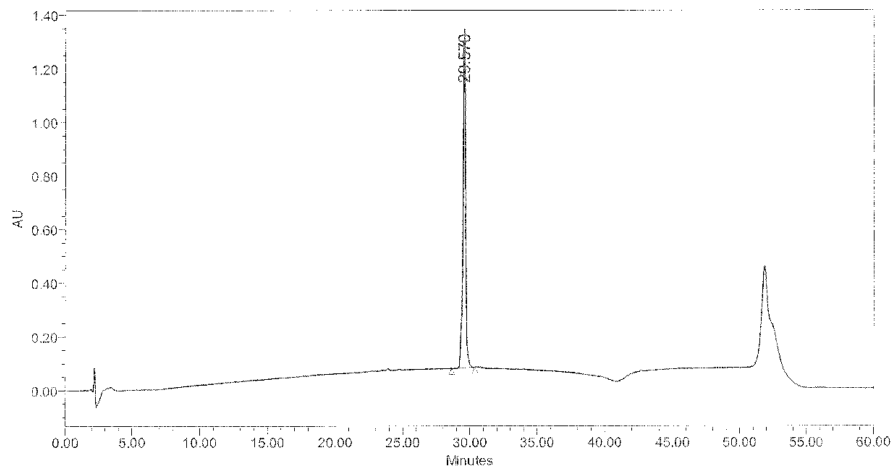

	RT	Area	% Area	Height
1	29.570	17339765	100.00	1263648

Cyclic depsipeptide (10)

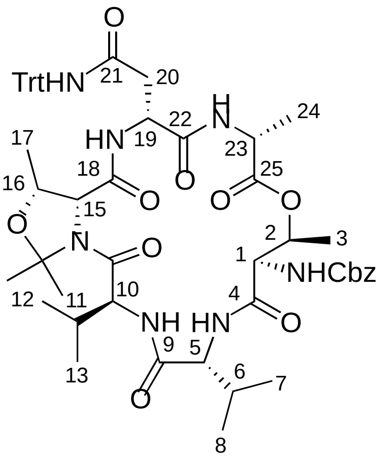

Compound **6** (65 mg, 64 µmol) was dissolved in dry toluene (30 mL) and added dropwise over 16 h to a solution of triethylamine (24 µL, 0.17 mmol), 2,4,6 trichlorobenzoyl chloride (16 µL, 0.10 mmol) and DMAP (37.6 mg, 0.308 mmol) in toluene (140 mL, final concentration of acid 0.001 M). After the addition, the solution was left under stirring for 72 h. The toluene was then removed under reduced pressure. The crude product was purified by flash chromatography (9:1 $CHCl_3$/MeOH) to afford **10** (18 mg, 28%, 5:1 mixture of diastereomers). Purification by Prep TLC (9:1 chloroform/methanol) afforded **10** as a colourless solid (15 mg, 24%); **mp** 260 °C (decomp.); $[\alpha]^{20}_D$ +35 (*c* 0.09, MeOH); **^{1}H NMR** (400 MHz, CD_3OD) δ 7.35–7.19 (20 H, m, ArH), 5.11 (1 H, m, H2), 4.99 (2 H, m, OC<u>H</u>$_2$Ar), 4.51 (1 H, m, H19), 4.42–4.32 (2 H, m, H16, H5), 4.40 (1 H, d, *J* = 6.9 Hz, H15), 4.22 (2 H, m, H1, H10), 3.94 (1 H, m, H23), 3.08 (2 H, m, H20), 2.14–2.03 (2 H, m, H6, H11), 1.82 (3 H, s, OC(C<u>H</u>$_3$)$_2$N), 1.71 (3 H, s, OC(C<u>H</u>$_3$)$_2$N), 1.38 (3 H, d, *J* =7.1 Hz, H24), 1.29 (3 H, d, *J* = 5.9 Hz, H3), 1.03 (3 H, d, *J* = 8.3 Hz, H17), 1.00 (6 H, d, *J* = 7.1 Hz, H12, H13), 0.90 (3 H, d, *J* = 6.4 Hz, H7), 0.79 (3 H, d, *J* = 6.9 Hz, H8); **^{13}C NMR** (100 MHz, CD_3OD) δ 174.0 (C=O, C22), 173.9 (C=O, C9), 173.3 (C=O, C14), 172.2 (C=O, C4), 171.7 (C=O, C25), 170.8 (C=O, C18), 170.3 (C=O, C21), 158.1 (C=O, NH<u>C</u>OOCH$_2$Ar), 146.0 (Cq, Ar), 137.9 (Cq, Ar), 130.1 (CH, Ar), 129.6 (CH, Ar), 129.5 (CH, Ar), 129.1 (CH, Ar), 128.8 (CH, Ar), 127.8 (CH, Ar), 96.0 (Cq, O<u>C</u>(CH$_3$)$_2$N), 72.4 (Cq, Ar), 71.7 (CH$_2$, O<u>C</u>H$_2$Ar), 71.5 (CH, C1), 68.3 (CH, C16), 66.7, (CH, C2), 62.0 (CH, C15), 60.2 (CH, C10), 59.5 (CH, C5), 57.5 (CH, C19), 50.6 (CH, C23), 50.2 (CH$_2$, C20), 32.0 (CH, C6), 30.4 (CH, C11), 27.7 (CH$_3$, OC(<u>C</u>H$_3$)$_2$N), 25.0 (CH$_3$, OC(<u>C</u>H$_3$)$_2$N), 19.9 (CH$_3$, C13), 19.8 (CH$_3$, C12), 18.7 (CH$_3$, C8), 18.1 (CH$_3$, C7), 18.0 (CH$_3$, C17), 16.3 (CH$_3$,

C24), 15.8 (CH_3, C3); **MS** (ESI) *m/z* 1024 [$(M+Na)^+$, 100%]; **HRMS** (ESI, MNa^+) Calcd for $C_{55}H_{69}N_7O_{12}Na$ 1024.4791 found 1024.4804.

Analytical HPLC trace of crude reaction mixture: expansion 20–40 min

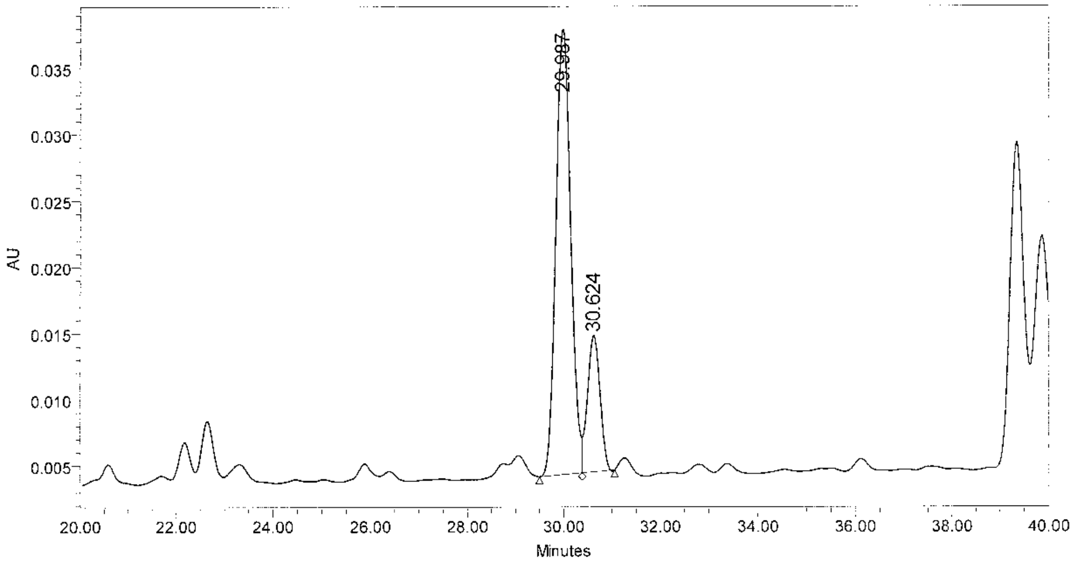

	RT	Area	% Area	Height
1	29.987	717209	79.32	33603
2	30.624	186971	20.68	10291

15-Hydroxypentadecanoic acid

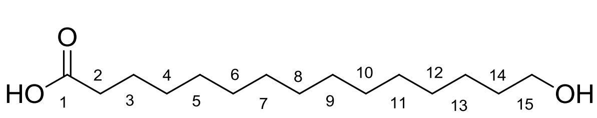

Pentadecanolide (5.0 g, 21 mmol) was dissolved in a 1:1 solution of methanol/THF (20 mL). To this solution was added NaOH (7.7 g, 0.19 mol) dissolved in H_2O (10 mL). The reaction mixture was stirred overnight before being partitioned between 3:1 chloroform/isopropanol (50 mL) and 1 M HCl (50 mL). The aqueous fraction was extracted with 3:1 chloroform/isopropanol (3 × 50 mL). The organic fractions were then combined and dried (Na_2SO_4), and the solvent was removed under reduced pressure. This afforded 15-hydroxypentadecanoic acid as a colourless solid (5.4 g, quant.). **mp** 82–85 °C (lit. 78 °C); **^{1}H NMR** (400 MHz, $CDCl_3$) δ 5.20 (2 H, br s, OHs), 3.64 (2 H, t, *J* = 6.6 Hz, H15), 2.34 (2 H, t, *J* = 7.3 Hz, H2), 1.67–1.53 (4 H, m, H3, H14), 1.37–1.30 (6 H, m, H4, H5,

H13), 1.26 (14 H, s, H6–H12); ^{13}**C NMR** (100 MHz, $CDCl_3$) δ 179.1 (C=O, C1), 63.1 (CH_2, C15), 34.0 (CH_2, C2), 29.6 (4 C, CH_2, C3–C6), 29.5(6), 29.5(3), 29.4(4), 29.4(1), 29.2, 29.1, 25.8, 24.8 (CH_2, C7–C14); **IR** (CH_2Cl_2): 3423, 2916, 2849, 1699 cm^{-1}; **MS** (ESI) *m/z* 259 [$(M+H)^+$, 100%], 242 (80). All data consistent with that reported in the literature [2].

1-Methyl-15-[bis(*tert*-Butoxycarbonyl)guanidino] pentadecanoate (15)

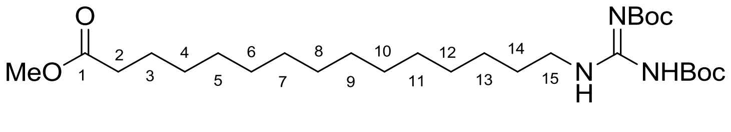

To a solution of 15-hydroxypentadecanoic acid (3.3 g, 13 mmol) in methanol (60 mL) was added acetyl chloride (3.0 mL, 39 mmol). This solution was heated under reflux for 16 h, then the solvent was then removed under reduced pressure and the residue azeotropically distilled with toluene (3 × 30 mL). The crude methyl ester **14** was then isolated as a white solid (3.5 g, 98%) and used without further purification.

Under an atmosphere of argon a solution of **14** (1.78 g, approx. 6.5 mmol), triphenylphosphine (3.4 g, 13 mmol) and 1-3-bis(*tert*-butoxycarbonyl) guanidine (2.6 g, 13 mmol) in THF (50 mL) was cooled to 0 °C then diisopropyl azodicarboxylate (2.6 mL, 13 mmol) was added dropwise. This solution was stirred for 16 h before the solvent was removed under reduced pressure. The residue was then purified by flash chromatography (4:1 hexane/Et_2O). This afforded **15** as a colourless oil (2.8 g, 86%).^{1}**H NMR** (400 MHz, $CDCl_3$) δ 9.40 (1 H, br s, NH), 9.24 (1 H, br s, NH), 4.05 (2 H, t, *J* = 6.6 Hz, H15), 3.88 (2 H, m, H2), 3.66 (3 H, s, $OC\underline{H}_3$), 2.29 (4 H, m, H3, H14), 1.64–1.55 (4 H, m, H4, H13), 1.57 (4 H, s, H5, H12), 1.51 (9 H, s, *tert*-butyl), 1.49 (9 H, s, *tert*-butyl), 1.25 (12 H, s, H6–H11); ^{13}**C NMR** (100 MHz, $CDCl_3$) δ 174.2 (C=O, C1), 164.1 (C=O, $N\underline{C}OO$*t*-Bu), 160.9 (C=O, $N\underline{C}OO$*t*-Bu), 155.3 (C=N, $NH\underline{C}NN$), 83.5 (Cq, *tert*-butyl), 78.9 (Cq, *tert*-butyl), 51.6 (CH_3, $O\underline{C}H_3$), 44.8 (CH_2, C15), 34.5 (CH_2, C2), 27.7 (CH_2, C14), 29.6(0), 29.6(1), 29.5, 29.4, 29.3(5), 29.2 (6), 29.2(4), 28.7 (CH_2, C4–C11), 28.3 (3 C, *tert*-butyl), 28.0 (3 C, *tert*-butyl), 26.8 (CH_2, C12), 26.0 (CH_2, C13), 25.1 (CH_2, C3); **MS** (ESI) *m/z* 514 [$(M+H)^+$, 100%], **HRMS** (ESI, MH^+) Calcd. for $C_{27}H_{51}N_3O_4$, 514.3851, found 514.3845; **IR** (CH_2Cl_2): 2926, 2854, 1738, 1713, 1609, 1510 cm^{-1}.

15-(*tert*-Butoxycarbonylguanidino) pentadecanoic acid (12)

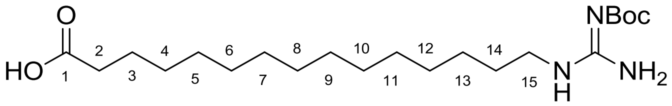

Compound **15** (2.4 g, 4.7 mmol) was dissolved in a 1:1 solution of methanol/THF (20 mL). To this solution was added NaOH (1.9 g, 47 mmol) dissolved in H_2O (10 mL). This solution was stirred for 16 h, before partitioning between 3 M NaOH (100 mL) and Et_2O (100 mL). The solution was then washed with Et_2O (3 × 100 mL). 1 M HCl was then added until a white precipitate formed. The solution was then extracted with EtOAc (3 × 100 mL). This afforded the title compound **12** as a colourless solid (1.8 g, 93%); **^{1}H NMR** (400 MHz, $CDCl_3$) δ 3.32 (2 H, t, J = 6.6 Hz, H15), 2.73 (4 H br s, NH_2, NH, OH), 2.29 (2 H, t, J = 7.2 Hz, H2), 1.71–1.58 (4 H, m, H3, H14), 1.50 (9 H, s, *tert*-butyl), 1.43–1.25 (20 H, m, H4–H13); **^{13}C NMR** (100 MHz, $CDCl_3$) δ 178.7 (C=O, C1), 154.3 (C=O, N<u>C</u>OO*t*-Bu), 152.7 (C=N, NH<u>C</u>NN), 85.1 (Cq, *tert*-butyl), 42.2 (CH_2, C15), 34.0 (CH_2, C2), 29.7 (CH_2, C14), 29.6, 29.5, 29.4, 29.3, 29.2, 29.1, 29.0, 29.0, 28.9 (CH_2, C4–C12), 28.0 (3 C, CH_3, *tert*-butyl), 26.5 (CH_2, C13), 24.7 (CH_2, C3); **MS** (ESI) *m/z* 400 [$(M+H)^+$, 100%]; **HRMS** (ESI, MH^+) Calcd. for $C_{21}H_{41}N_3O_4$, 400.3170; found 400.3169; **IR** (CH_2Cl_2): 3163, 2923, 2853, 1737, 1709, 1679 cm^{-1}.

Cyclic depsipeptide (17)

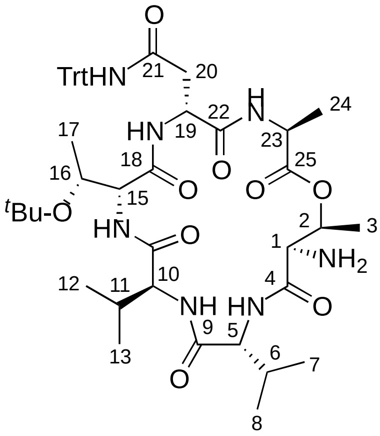

Compound **8** (0.18 g, 0.18 mmol) was dissolved in 20 mL of dry THF, and Pd/C catalyst (10% w/w, 0.1 g) was added. The reaction flask was evacuated and purged with H_2 three times, and the solution was left under stirring in an atmosphere of H_2 for 24 h. More Pd/C catalyst (10% w/w, 50 mg) was then added and the solution was left under stirring in an atmosphere of H_2 for another 24 h.

The solution was then filtered through a pad of Celite® and the solvent removed under reduced pressure. The residue was purified by preparative RP-HPLC under the standard conditions. The amine **8** was isolated after lyophilisation as an amorphous white solid (80 mg, 50%); **mp** 177–180 °C; $[\alpha]^{20}_{D}$ +24 (*c* 0.05, MeOH); ^{1}**H NMR** (400 MHz, CD_3OD) δ 8.63 (2 H, s, NHs), 8.52 (1 H, d, *J* = 8.3 Hz, NH), 8.13 (1 H, d, *J* = 6.9 Hz, NH), 7.61 (1 H, d, *J* = 8.8 Hz, NH), 7.30–7.21 (17 H, m, ArH, NH), 5.13 (1 H, dq, *J* = 2.9, 6.6 Hz, H2), 4.86 (1 H, m, H19), 4.57 (1 H, dd, *J* = 4.4, 7.83 Hz, H15), 4.35 (2 H, m, H16, H5), 4.09 (1 H, m, H10), 4.06 (1 H, d, *J* = 2.9 Hz, H1), 4.00 (1 H, m, H23), 2.88 (2 H, m, H20), 2.16 (1 H, m, H6), 1.94 (1 H, m, H11), 1.35 (3 H, d, *J* = 6.6 Hz, H24), 1.29 (3 H, d, *J* = 6.6 Hz, H3), 1.89 (9 H, s, *tert*-butyl), 1.07 (3 H, d, *J* = 6.6 Hz, H17), 1.05 (3 H, d, *J* = 6.6 Hz, H7), 1.02 (3 H, d, *J* = 6.06 Hz, H8), 0.96 (6 H, d, *J* = 6.6 Hz, H12, H13); ^{13}**C NMR** (100 MHz, CD_3OD) δ 175.8 (C=O, C14), 173.7 (C=O, C25), 172.9 (C=O, C22), 172.7 (C=O, C9), 171.6 (C=O, C21), 171.3 (C=O, C18), 166.7 (C=O, C4), 145.8 (Cq, Ar), 130.0 (CH, Ar), 128.9 (CH, Ar), 128.7 (CH, Ar), 128.1 (CH, Ar), 127.9 (CH, Ar), 75.3 (Cq, *tert*-butyl), 71.8 (Cq, Ar), 69.5 (CH, C2), 66.9 (CH, C16), 62.0 (CH, C10), 60.1 (CH, C5), 60.1 (CH, C15), 54.9 (CH, C1), 51.1 (CH, C23), 49.7 (CH, C19), 40.9 (CH_2, C20), 30.6 (CH, C6), 30.3 (CH, C11), 28.1 (3 C, CH_3, *tert*-butyl), 19.8 (CH_3, C8), 19.5 (CH_3, C7), 19.4 (CH_3, C12), 18.8 (CH_3, C13), 17.8 (CH_3, C17), 15.6 (CH_3, C24), 13.8 (CH_3, C3); **MS** (ESI) *m/z* 884 [$(M+H)^+$, 100%], 1768 (50); **HRMS** (ESI, MH^+) Calcd. for $C_{48}H_{66}N_7O_9$ 884.4917 found 884.4915.

Cyclic depsipeptide 22

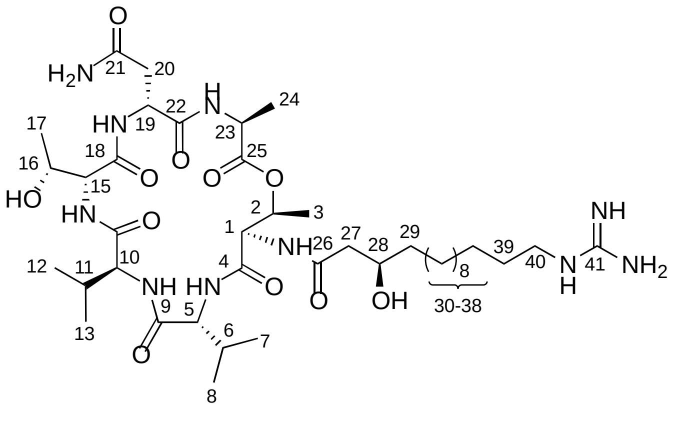

A solution of the acid **18** (19 mg, 35 µmol), HATU (18 mg, 47 µmol) and diisopropylethylamine (10 µL, 55 µmol) in DMF (0.5 mL) was added to a solution of the amine **17** (20 mg, 23 µmol) in DMF (0.5 mL). The mixture was shaken on an orbital shaker (175 rpm) for 16 h. The crude reaction mixture was then purified by RP-HPLC under the standard conditions. The protected compound was then treated with a solution of 90:5:5 TFA/H_2O/CH_2Cl_2 for 3 h. The

solvent was removed under reduced pressure and the residue azeotropically distilled with toluene (3 × 30 mL). The residue was then purified by semipreparative RP-HPLC (0–50% B over 40 min). This gave **22** after lyophilisation as a colourless solid (3.3 mg, 17%); **$[\alpha]^{20}_{D}$** +2 (*c* 0.05, MeOH); **^{1}H NMR** (400 MHz, DMSO-d_6) δ 8.64 (1 H, d, *J* = 8.0 Hz, C5NH), 8.52 (1 H, d, *J* = 4.9 Hz, C10NH), 8.49 (1 H, d, *J* = 4.2 Hz, C15NH), 7.69 (1 H, br s, C21NHH), 7.57 (1 H, br m, C41 NH), 7.42 (1 H, d, *J* = 6.0 Hz, C19NH), 7.40 (2 H, d, *J* = 5.0 Hz, C1NH, C40NH), 7.24 (1 H, br s, C21NHH), 6.77 (1 H, d, *J* = 5.0 Hz, C23NH), 5.14 (1 H, dq, *J* = 6.6 Hz, 3.4, H2), 5.05 (1 H, d, *J* = 5.18 Hz, C28OH), 5.03 (1 H, d, *J* = 4.0 Hz, C16OH), 4.45 (1 H, m, H19), 4.40 (2 H, m, H1, H5), 4.05 (1 H, m, H16), 3.99–3.92 (3 H, m, H23, H15, H10), 3.78 (1 H, m, H28), 3.06 (2 H, dd, *J* = 13.9, 6.6 Hz, H40), 2.72 (1 H, dd, *J* = 14.0, 6.6 Hz, H20a), 2.60 (1 H, dd, *J* = 14.4, 8.3 Hz, H20b), 2.47 (1 H, dd, *J* = 13.5, 5.1 Hz, H27a), 2.24 (1 H, dd, *J* = 13.5, 6.0 Hz, H27b), 1.88 (2 H, m, H6, H11), 1.44 (2 H, m, H39), 1.37 (2 H, m, H29), 1.23 (18 H, br s, H30–H38), 1.20 (3 H, d, *J* = 7.3 Hz, H24), 1.17 (3 H, d, *J* = 6.60 Hz, H3), 1.12 (3 H, d, *J* = 6.6 Hz, H17), 0.98 (3 H, d, *J* = 6.6 Hz, H13), 0.90 (3 H, d, *J* = 6.6 Hz, H12), 0.83 (3 H, d, *J* = 6.6 Hz, H7), 0.77 (3 H, d, *J* = 6.8 Hz, H8), (2 guanidine NH protons 7.50–6.70 ppm); **^{13}C NMR** (150 MHz, DMSO-d_6) δ 174.3 (C=O, C14), 172.2 (C=O, C22), 172.1 (C=O, C26), 171.9 (C=O, C9), 171.6 (C=O, C25), 170.4 (C=O, C21), 169.4 (C=O, C18), 168.4 (C=O, C4), 156.7 (C=N, C41), 71.1 (CH, C2), 67.6 (CH, C28), 65.5 (CH, C16), 60.6 (CH, C15), 59.7 (CH, C10), 56.9 (CH, C5), 56.1 (CH, C1), 51.2 (CH, C19), 48.2 (CH, C23), 42.8 (CH_2, C27), 40.7 (CH_2, C40), 38.1 (CH_2, C29), 36.7 (CH, C20), 31.8 (CH, C6), 29.1 (CH, C11), 29.0 (5 C, CH_2 C31–C35), 28.8 (CH_2, C37), 28.6 (CH_2, C36), 28.4 (CH_2, C39), 26.0 (CH_2, C38), 25.4 (CH_2, C30), 19.2 (CH_3, C17), 19.1 (CH_3, C13), 18.9 (CH_3, C8), 18.2 (CH_3, C7), 18.1 (CH_3, C12), 16.6 (CH_3, C24), 15.4 (CH_3, C3); **MS** (ESI) *m/z* 883 $[(M+H)^+$, 100%], 1019 (20); **HRMS** (ESI, MH^+) Calcd. for $C_{41}H_{74}N_{10}O_{11}$ 883.5611 found 883.5622.

Cyclic depsipeptide 23

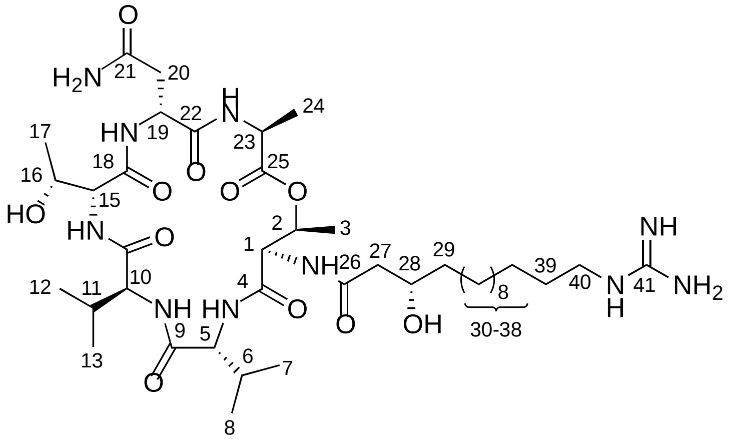

A solution of the acid **19** (11 mg, 20 µmol), HATU (15 mg, 39 µmol) and diisopropylethylamine (6.6 µL, 39 µmol) in DMF (0.5 mL) was added to a solution of the amine **17** (15 mg, 17 µmol) in DMF (0.5 mL). The mixture was shaken on an orbital shaker (175 rpm) for 16 h. The crude reaction mixture was then purified by RP-HPLC under the standard conditions. The protected compound was then treated with a solution of TFA/H_2O/CH_2Cl_2 (90:5:5) for 3 h. The solvent was removed under reduced pressure and the residue azeotropically distilled with toluene (3 × 30 mL). The residue was then purified by semipreparative RP-HPLC (0–50% B over 40 min). This gave **23** after lyophilisation as a colourless solid (2.4 mg, 16%); **$[\alpha]^{20}_{D}$** -2 (*c* 0.05, MeOH); **^{1}H NMR** (400 MHz, DMSO-d_6) δ 8.58 (1 H, d, *J* = 7.4 Hz, C10NH), 8.54 (1 H, d, *J* = 4.6 Hz, C15NH), 8.50 (1 H, d, *J* = 3.8 Hz, C1NH), 7.64 (1 H, br s, C21N<u>H</u>H), 7.53 (1 H, br m, C41NH), 7.44 (1 H, d, *J* =7.8 Hz, C19NH), 7.40 (1 H, m, C40NH), 7.30 (1 H, br s. C5NH), 7.23 (1 H, br s, C21NH<u>H</u>), 6.71 (1 H, d, *J* = 5.1 Hz, C23NH), 5 .13 (1 H, dq, *J* = 6.6, 3.3 Hz, H2), 5.03 (1 H, d, *J* = 3.7 Hz, C16OH), 4.85 (1 H, d, *J* = 4.0 Hz, C28OH), 4.47–4.40 (2 H, m, H19, H5), 4.33 (1 H, dd, *J* = 7.5, 3.3 Hz, H1), 4.07 (1 H, m, H16), 4.00–3.93 (2 H, m, H23, H15), 3.88 (1 H, dd, *J* = 8.3, 4.0 Hz, H10), 3.79 (1 H, m, H28), 3.07 (2 H, dd, *J* = 7.8, 14.7 Hz, H40), 2.69 (2 H, m, H20), 2.39 (1 H, dd, *J* = 13.2, 9.6 Hz, H27a), 2.16 (1 H, dd, *J* = 13.2, 3.3 Hz, H27b), 1.87 (2 H, m, H6, H11), 1.44 (4 H, m, H39, H29), 1.24 (18 H, br m, H30–H38), 1.21 (3 H, d, *J* = 6.6 Hz, H24), 1.20 (3 H, d, *J* = 7.1 Hz, H3), 1.11 (3 H, d, *J* = 6.6 Hz, H17), 0.99 (3 H, d, *J* = 6.6 Hz, H13), 0.90 (3 H, d, *J* = 6.6 Hz, H12), 0.83 (3 H, d, *J* = 6.6 Hz, H7), 0.76 (3 H, d, *J* = 6.6 Hz, H8), (2 guanidine NH protons 7.50–6.70 ppm); **^{13}C NMR** (150 MHz, DMSO-d_6) δ 174.9 (C=O, C14), 173.0 (C=O, C22), 172.7 (C=O, C26), 172.5 (C=O, C9), 172.2 (C=O, C25), 171.0 (C=O, C21), 170.0 (C=O, C18), 168.6 (C=O, C4), 157.2 (C=N, C41), 71.6 (CH, C2), 69.1 (CH, C28), 65.8 (CH, C16), 60.9 (CH, C15), 60.5 (CH, C10), 57.8 (CH, C5), 56.5 (CH, C1), 52.1 (CH, C19), 48.5 (CH, C23), 43.3 (CH_2, C27), 41.2

(CH_2, C40), 38.7 (CH_2, C29), 37.6 (CH, C20), 32.4 (CH, C6), 29.7 (CH, C11), 29.5(2) (4 C, CH_2 C31–C34), 29.5 (CH_2, C35), 29.1(3) (CH_2, C37), 29.1(0) (CH_2, C36), 29.0 (CH_2, C39), 26.5 (CH_2, C38), 25.2 (CH_2, C30), 19.8(1) (CH_3, C17), 19.8(0) (CH_3, C13), 19.4 (CH_3, C8), 18.8 (CH_3, C7), 18.4 (CH_3, C12), 17.1 (CH_3, C24), 15.9 (CH_3, C3); **MS** (ESI) *m/z* 883 [$(M+H)^+$, 100%], 1019 (20); **HRMS** (ESI, MH^+) Calcd. for $C_{41}H_{74}N_{10}O_{11}$ 883.5611 found 883.5589.

Dehydroxy LI-F04a Analogue 21

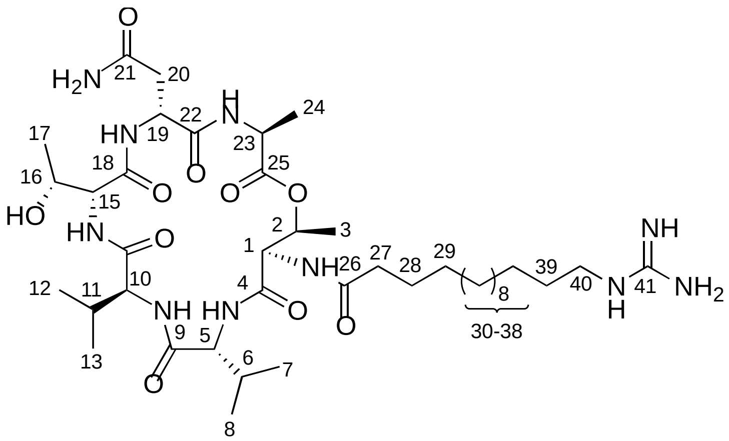

A solution of the acid **12** (18 mg, 36 µmol), HATU (16 mg, 40 µmol) and diisopropylethylamine (12 µL, 72 µmol) in DMF (0.5 mL) was added to a solution of the amine **7** (11 mg, 12 µmol) in DMF (0.5 mL). The mixture was shaken on an orbital shaker (175 rpm) for 16 h. The crude reaction mixture was then purified by RP-HPLC under the standard conditions. The protected compound was then treated with a solution of 90:5:5 TFA/H_2O/CH_2Cl_2 for 3 h. The solvent was removed under reduced pressure and the residue azeotropically distilled with toluene (3 × 30 mL). The residue was then purified by semipreparative RP-HPLC (0–50% B over 40 min). This gave **21** after lyophilisation as a colourless solid (3.2 mg, 30%); **$[\alpha]^{20}_D$** +12 (*c* 0.06, MeOH); **^{1}H NMR** (400 MHz, DMSO-d_6) δ 8.44–8.39 (3 H, m, C1NH, C15NH, C10NH), 8.20 (1 H, br s, C19NH), 7.48 (1 H, t, *J* = 5.4 Hz, C40NH), 7.45 (1 H, br s, C21NHH), 7.07 (1 H, d, *J* = 7.7 Hz, C23NH), 7.00–6.99 (2 H, m, C21NHH, C5NH), 5.30 (1 H, dq, *J* = 6.0, 2.0 Hz, H2), 4.95 (1 H, d, *J* = 4.5 Hz, C16OH), 4.49 (1 H, dd, *J* = 9.2, 7.4 Hz, H5), 4.37 (1 H, dd, *J* = 8.3, 2.0 Hz, H1), 4.28–4.20 (2 H, m, H19, H10), 4.10 (1 H, m, H23), 3.91 (2 H, m, H15, H16), 3.06 (2 H, dd, *J* = 14.6, 6.7 Hz, H40), 2.77 (1 H, dd, *J* = 15.0, 7.4 Hz, H20a), 2.45 (1 H, dd, *J* = 15.0, 6.4 Hz, H20b), 2.29 (2 H, m, H27), 2.01 (1 H, m, H11), 1.79 (1 H, m, H6), 1.51 (2 H, m, H28), 1.44 (2 H, m, H39), 1.23 (20 H, br s, H29–H38), 1.15 (3 H, d, *J* = 6.6 Hz, H3), 1.12 (3 H, d, *J* = 7.4 Hz, H24), 1.09 (3 H, d, *J* = 6.0 Hz, H17), 0.84 (12 H, d, *J* = 6.8 Hz, H13, H12), 0.81 (3 H, d, *J* = 6.8 Hz, H7), 0.74 (3 H, d, *J* =

6.8 Hz, H8), (3 guanidine NH protons 7.32–6.60 br s); ^{13}**C NMR** (100 MHz, DMSO-d_6) δ 173.6 (C=O, C14), 172.5 (C=O, C22), 172.2 (C=O, C26), 170.7 (C=O, C9), 170.5 (C=O, C25), 170.4 (C=O, C18), 169.6 (C=O, C21), 168.4 (C=O, C4), 156.6 (C=N, C41), 70.2 (CH, C2), 65.7 (CH, C16), 60.0 (CH, C15), 57.7 (CH, C10), 57.3 (CH, C5), 56.2 (CH, C1), 50.5 (CH, C19), 47.7 (CH, C23), 40.7 (CH_2, C40), 36.7 (CH_2, C20), 34.8 (CH_2, C27), 31.9 (CH, C6), 30.1 (CH, C11), 28.9 (6 C, CH_2, C29, C31–C34), 28.8 (CH_2, C35), 28.6 (CH_2, 37), 28.6 (CH_2, C36), 28.4 (CH_2, C39), 26.0 (CH_2, C38), 25.4 (CH_2, C30), 19.7 (CH_3, C17), 19.3 (CH_3, C13), 19.0 (CH_3, C8), 17.8 (CH_3, C7), 17.8 (CH_3, C12), 17.4 (CH_3, C24), 16.3 (CH_3, C3); **MS** (ESI) *m/z* 867 [(M+H)$^+$, 100%]; **HRMS** (ESI, MH^+) Calcd. for $C_{41}H_{74}N_{10}O_{10}$, 867.5662; found 867.5676.

Table S1: Temperature dependence of amide proton chemical shifts of **1**

Residue	δH 300K	δH 325K	Δ ppb/K	N–H bonded[3]
L-Thr-NH	8.25	8.10	6.0	–
D-Val-NH	7.27	7.17	4.2	–
L-Val-NH	8.39	8.25	5.6	–
D-*allo*-Thr-NH	8.44	8.27	6.8	–
D-Asn-NH	8.15	7.93	8.8	–
D-Asn-NH_2	7.04	6.92	4.8	–
D-Asn-NH_2	7.43	7.36	2.8	+
D-Ala-NH	7.17	7.26	–3.6	+

^{1}H NMR (400 MHz, DMSO-d_6)

References

1. Cochrane, J. R.; McErlean, C. S. P.; Jolliffe, K. A. *Org. Lett.*, **2010**, *12*, 3394
2. Nesmeyanov, A. N.; Zakharkin, L. I.; Kost, T. A.; Friedlina, R. K. *Russ. Chem. Bull.* **1960**, *9*, 195.
3. Kessler, H. *Angew. Chem., Int. Ed.* **1982**, *21*, 512
