# Supplementary material for: A macrolactonization approach to the total synthesis of the antimicrobial cyclic depsipeptide LI-F04a and diastereoisomeric analogues
Source: Beilstein J Org Chem. 2012 Aug 21;8:1344–51. doi: 10.3762/bjoc.8.154 (PMC3458759; doi:10.3762/bjoc.8.154)

Supporting Information File 2
for

A macrolactonization approach to the total synthesis of the antimicrobial cyclic depsipeptide LI-F04a and diastereoisomeric analogues

James R. Cochrane, Dong Hee Yoon, Christopher S. P. McErlean and Katrina A. Jolliffe*

Address: School of Chemistry, The University of Sydney, 2006, NSW, Australia;

Tel: +61-2-93512297; Fax: +61-2-93513329

Email: Katrina A. Jolliffe - kate.jolliffe@sydney.edu.au

* Corresponding author

^{1}H, ^{13}C and 2D NMR data for all new compounds

Cbz-L-Thr-D-Val-L-Val-D-*allo*-Thr(ΨPro)-D-Asn(Trt)-D-Ala-OH (6)

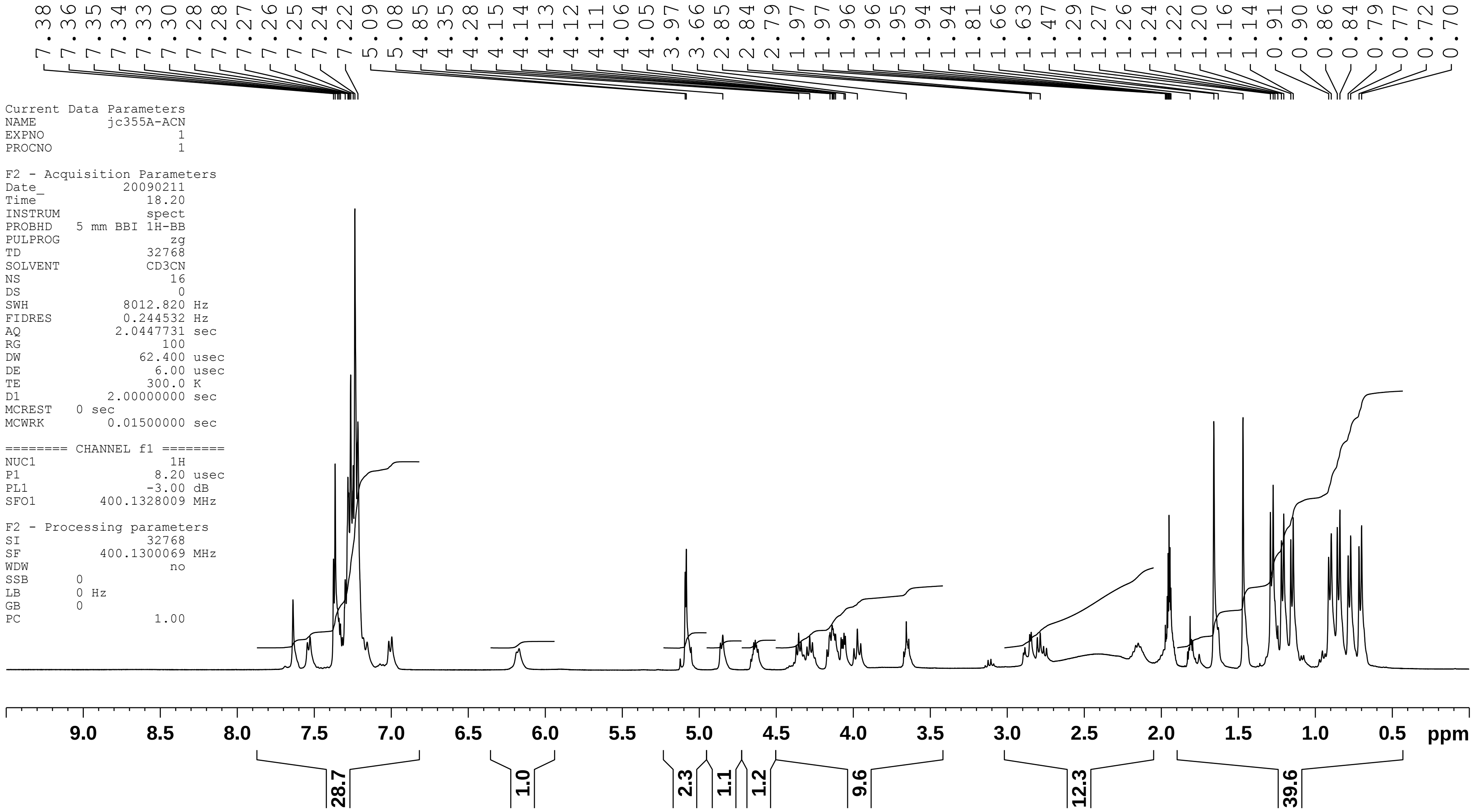

Cbz-L-Thr-D-Val-L-Val-D-*allo*-Thr(ΨPro)-D-Asn(Trt)-D-Ala-OH (6) COSY

```
Current Data Parameters
NAME          jc355A-ACN
EXPNO                  5
PROCNO                 1

F2 - Acquisition Parameters
Date_           20090212
Time                3.34
INSTRUM            spect
PROBHD   5 mm BBI 1H-BB
PULPROG      cosygpmfph
TD                  4000
SOLVENT             MeOH
NS                     4
DS                    16
SWH             3591.954 Hz
FIDRES          0.897988 Hz
AQ             0.5568500 sec
RG                  1024
DW               139.200 usec
DE                  6.00 usec
TE                 300.0 K
d0            0.00012840 sec
D1            2.50000000 sec
d13           0.00000400 sec
D16           0.00015000 sec
d20           0.00165400 sec
IN0           0.00027769 sec
MCREST   0 sec
MCWRK         1.25000000 sec
ST1CNT                 0

======== CHANNEL f1 ========
NUC1                  1H
P1                  8.20 usec
p2                 16.40 usec
PL1                -3.00 dB
SFO1         400.1318006 MHz

====== GRADIENT CHANNEL =====
GPNAM1          sine.100
GPNAM2          sine.100
GPX1     0 %
GPX2     0 %
GPY1     0 %
GPY2     0 %
GPZ1               10.00 %
GPZ2               20.00 %
P16              1500.00 usec

F1 - Acquisition parameters
TD                   256
SFO1            400.1318 MHz
FIDRES         14.067072 Hz
SW                 9.000 ppm
FnMODE       States-TPPI

F2 - Processing parameters
SI                  2048
SF           400.1300000 MHz
WDW                 SINE
SSB                    2
LB       0 Hz
GB       0
PC                  1.40

F1 - Processing parameters
SI                  4096
MC2          States-TPPI
SF           400.1300000 MHz
WDW
SSB                    2
LB       0 Hz
GB       0
```

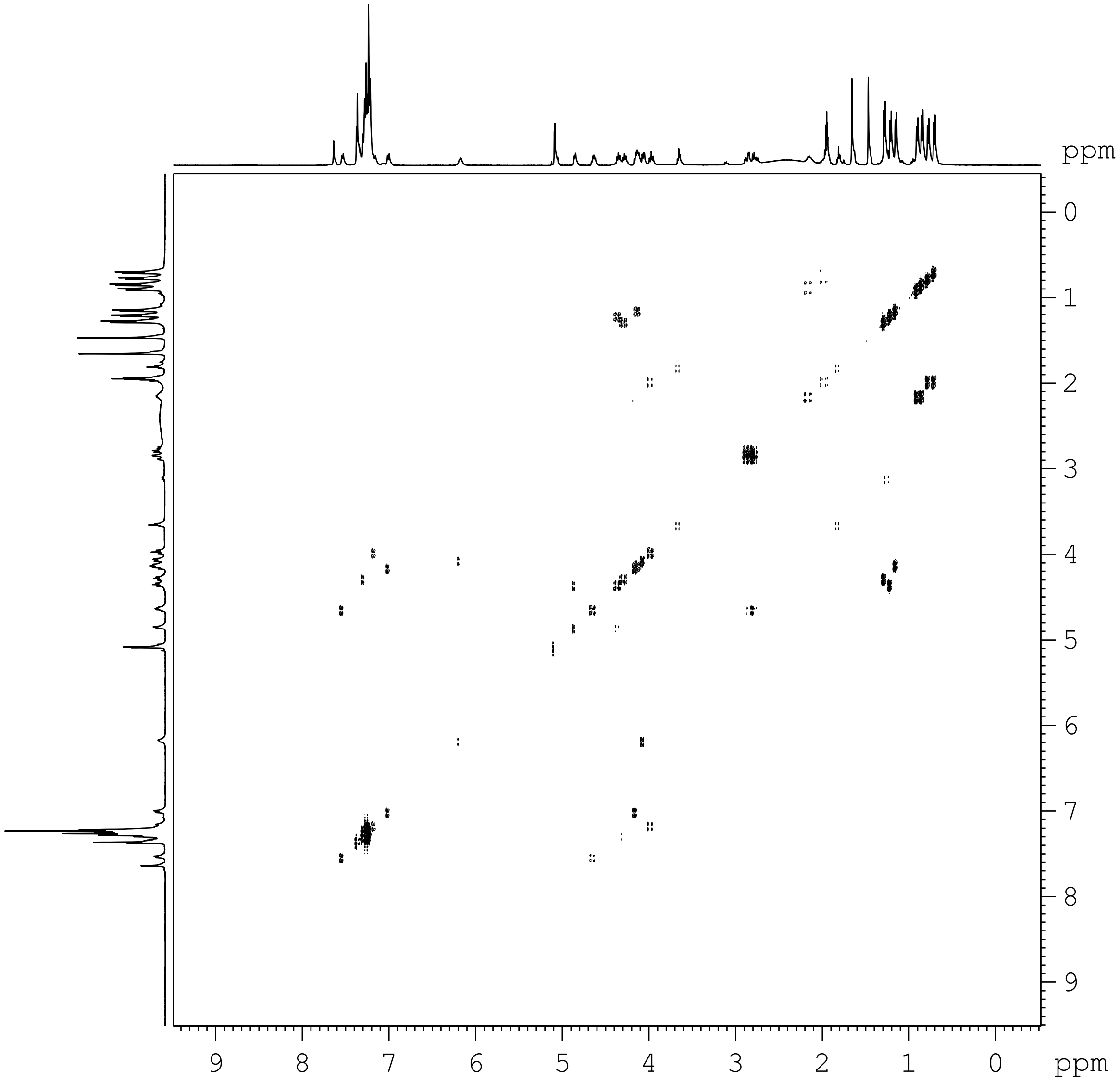

Cbz-L-Thr-D-Val-L-Val-D-*allo*-Thr(ΨPro)-D-Asn(Trt)-D-Ala-OH (6)

```
Current Data Parameters
NAME          jc355A-ACN
EXPNO                  2
PROCNO                 1

F2 - Acquisition Parameters
Date_           20090211
Time               18.24
INSTRUM            spect
PROBHD   5 mm BBI 1H-BB
PULPROG           zgdc30
TD                 65536
SOLVENT             C6D6
NS                  5120
DS                     0
SWH            26178.010 Hz
FIDRES          0.399445 Hz
AQ             1.2517875 sec
RG                  90.5
DW                19.100 usec
DE                  6.00 usec
TE                 300.0 K
D1            1.00000000 sec
d11           0.03000000 sec
MCREST   0 sec
MCWRK         0.01500000 sec

======== CHANNEL f1 ========
NUC1                 13C
P1                 12.60 usec
PL1                -6.00 dB
SFO1         100.6248025 MHz

======== CHANNEL f2 ========
CPDPRG2          waltz16
NUC2                  1H
PCPD2              90.00 usec
PL2                -3.00 dB
PL12               20.00 dB
SFO2         400.1320007 MHz

F2 - Processing parameters
SI                 32768
SF           100.6126690 MHz
WDW                   EM
SSB      0
LB                  2.00 Hz
GB       0
PC                  1.00
```

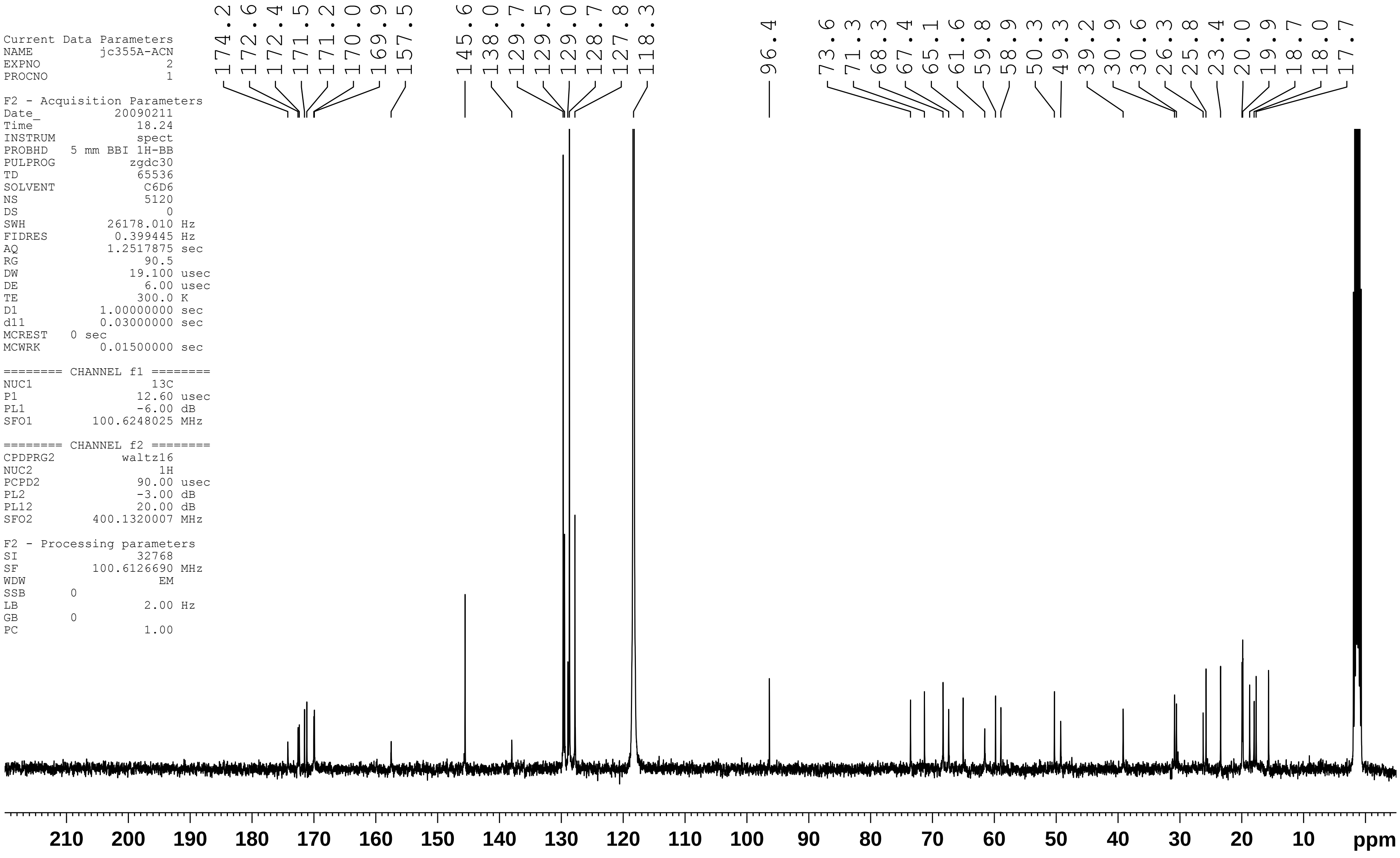

Cbz-L-Thr-D-Val-L-Val-D-*allo*-Thr(ΨPro)-D-Asn(Trt)-D-Ala-OH (6) HSQC

```
Current Data Parameters
NAME          jc355A-ACN
EXPNO                  3
PROCNO                 1

F2 - Acquisition Parameters
Date_           20090211
Time               21.39
INSTRUM            spect
PROBHD   5 mm BBI 1H-BB
PULPROG       invietgpsi
TD                  4096
SOLVENT            CDCl3
NS                    16
DS                     0
SWH             3591.954 Hz
FIDRES          0.876942 Hz
AQ             0.5702132 sec
RG                  1024
DW               139.200 usec
DE                  6.00 usec
TE                 300.0 K
CNST2        140.0000000
d0            0.00000300 sec
D1            2.00000000 sec
d4            0.00178571 sec
d11           0.03000000 sec
d13           0.00000400 sec
D16           0.00015000 sec
D24           0.00178500 sec
DELTA         0.00172240 sec
DELTA1        0.00165800 sec
IN0           0.00002259 sec
MCREST   0 sec
MCWRK         0.33333400 sec
ST1CNT                 0

======== CHANNEL f1 ========
NUC1                  1H
P1                  8.20 usec
p2                 16.40 usec
P28                 0.50 usec
PL1                -3.00 dB
SFO1         400.1318006 MHz

======== CHANNEL f2 ========
CPDPRG2             garp
NUC2                 13C
P3                 12.00 usec
p4                 24.00 usec
PCPD2              70.00 usec
PL2                -6.00 dB
PL12               10.70 dB
SFO2         100.6227903 MHz

====== GRADIENT CHANNEL =====
GPNAM1          sine.100
GPNAM2          sine.100
GPX1     0 %
GPX2     0 %
GPY1     0 %
GPY2     0 %
GPZ1               80.00 %
GPZ2               20.10 %
P16              1500.00 usec

F1 - Acquisition parameters
TD                   256
SFO1            100.6228 MHz
FIDRES         86.469284 Hz
SW               219.991 ppm
FnMODE     Echo-Antiecho

F2 - Processing parameters
SI                  1024
SF           400.1299808 MHz
WDW                QSINE
SSB      0
LB       0 Hz
GB       0
PC                  1.00

F1 - Processing parameters
SI                  1024
MC2        echo-antiecho
SF           100.6126591 MHz
WDW
SSB      0
LB       0 Hz
GB       0
```

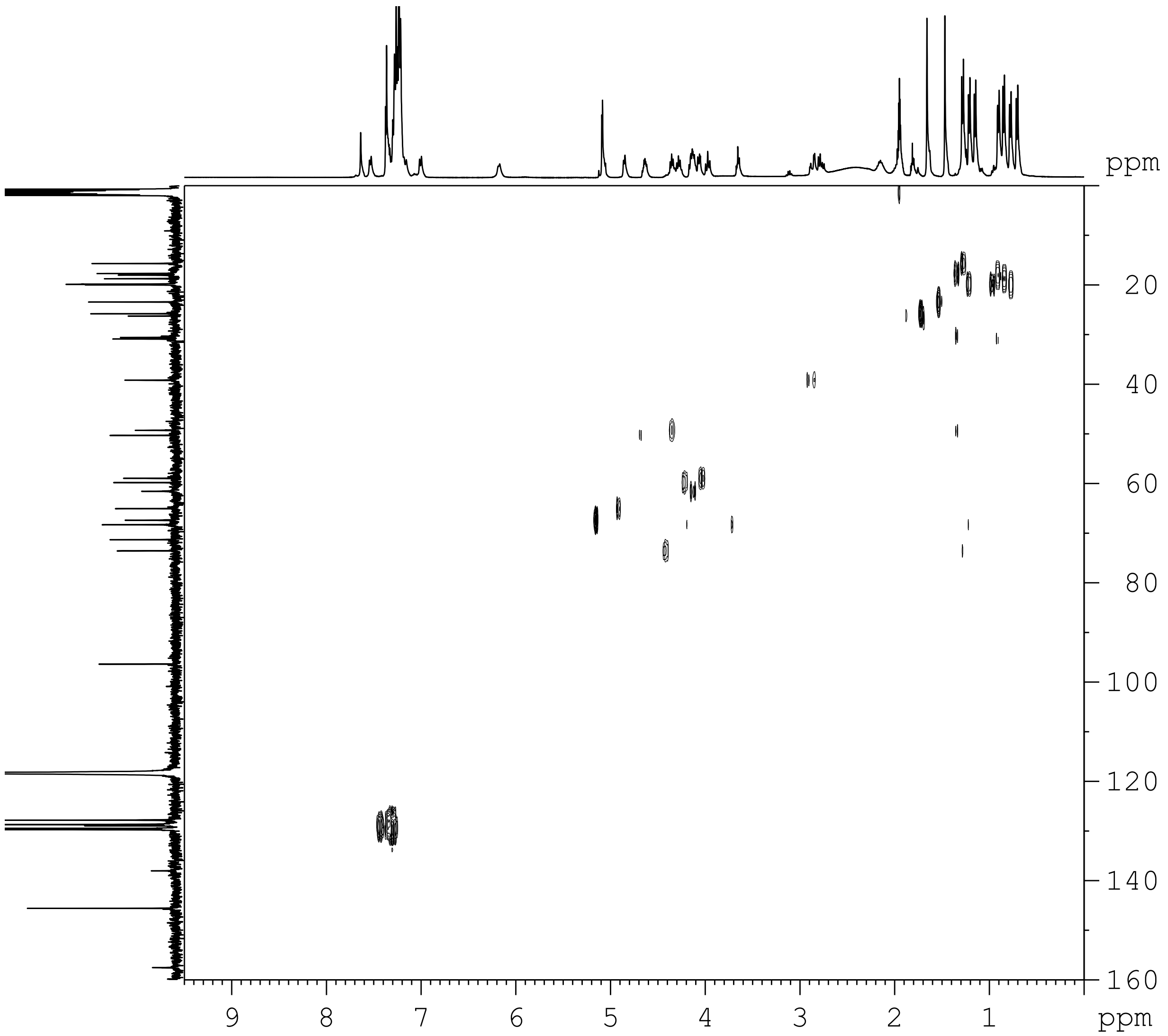

Cbz-L-Thr-D-Val-L-Val-D-*allo*-Thr(ΨPro)-D-Asn(Trt)-D-Ala-OH (6) HMBC

```
Current Data Parameters
NAME          jc355A-ACN
EXPNO                  1
PROCNO                 1

F2 - Acquisition Parameters
Date_          20090211
Time              18.20
INSTRUM           spect
PROBHD   5 mm BBI 1H-BB
PULPROG              zg
TD                32768
SOLVENT           CD3CN
NS                   16
DS                    0
SWH            8012.820 Hz
FIDRES         0.244532 Hz
AQ            2.0447731 sec
RG                  100
DW               62.400 usec
DE                 6.00 usec
TE                300.0 K
D1           2.00000000 sec
MCREST   0 sec
MCWRK        0.01500000 sec

======== CHANNEL f1 ========
NUC1                 1H
P1                 8.20 usec
PL1               -3.00 dB
SFO1        400.1328009 MHz

F2 - Processing parameters
SI                32768
SF          400.1300069 MHz
WDW                  no
SSB      0
LB       0 Hz
GB       0
PC                 1.00
```

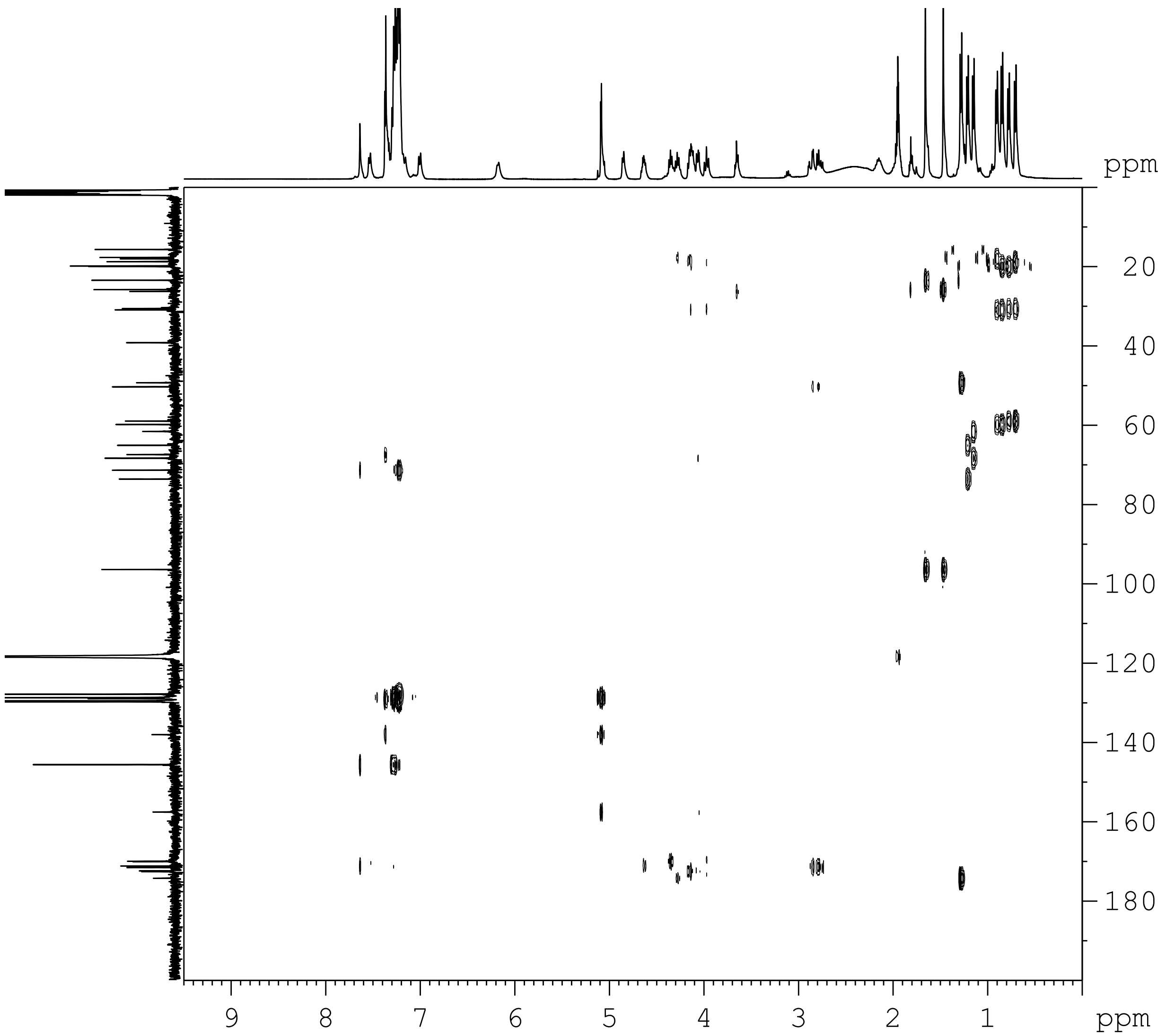

Cyclic depsipeptide (11)

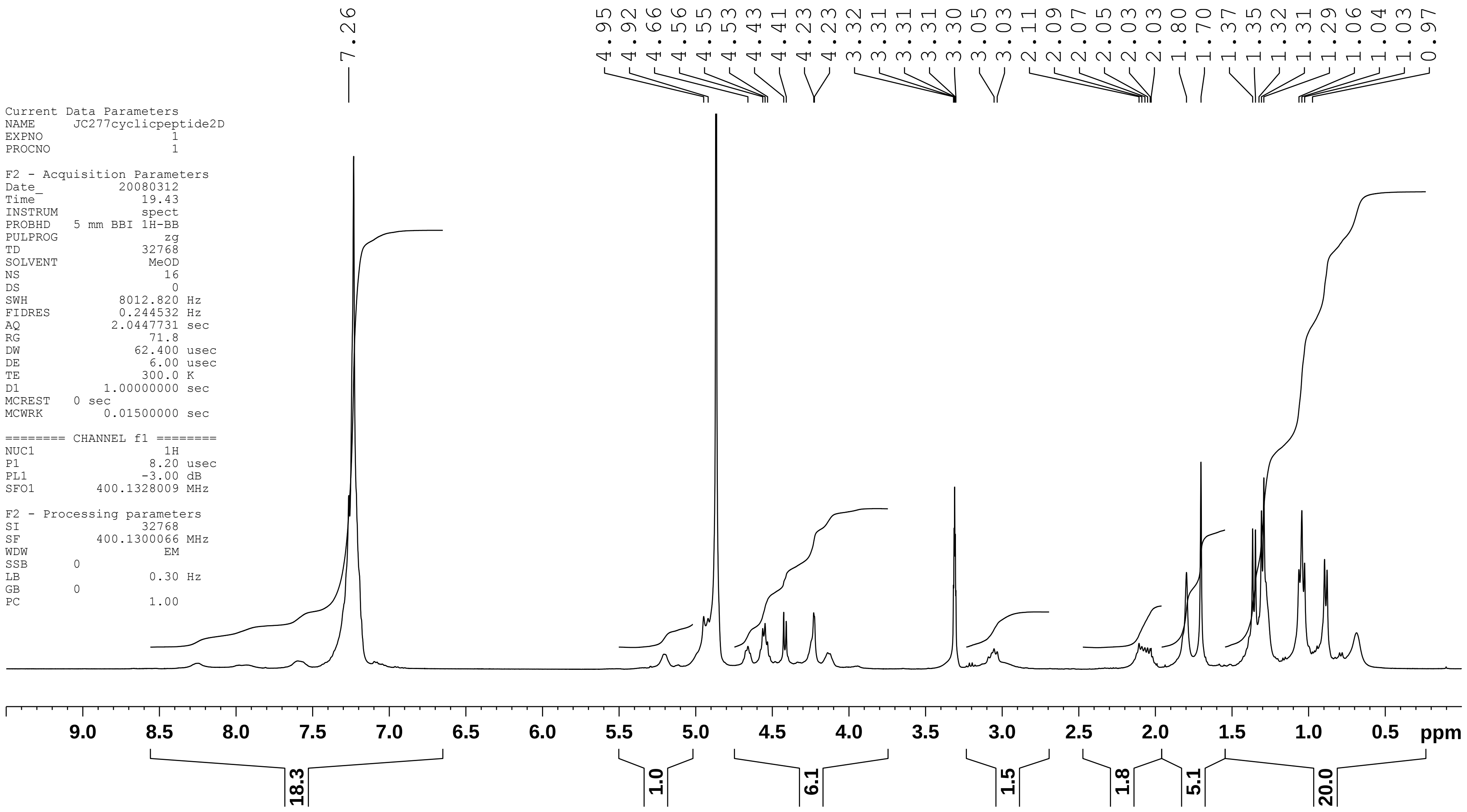

Cyclic depsipeptide (11) COSY

```
Current Data Parameters
NAME     JC277cyclicpeptide2D
EXPNO                  2
PROCNO                 1

F2 - Acquisition Parameters
Date_          20080312
Time              19.43
INSTRUM           spect
PROBHD   5 mm BBI 1H-BB
PULPROG        cosygpqf
TD                 2048
SOLVENT            MeOH
NS                    4
DS                    0
SWH            8012.820 Hz
FIDRES         3.912510 Hz
AQ            0.1278452 sec
RG                 90.5
DW               62.400 usec
DE                 6.00 usec
TE                300.0 K
d0           0.00000300 sec
D1           2.00000000 sec
d13          0.00000400 sec
D16          0.00015000 sec
IN0          0.00012480 sec
MCREST   0 sec
MCWRK        2.00000000 sec

======== CHANNEL f1 ========
NUC1                 1H
P0                 8.20 usec
P1                 8.20 usec
PL1               -3.00 dB
SFO1        400.1320007 MHz

====== GRADIENT CHANNEL =====
GPNAM1         sine.100
GPNAM2         sine.100
GPX1     0 %
GPX2     0 %
GPY1     0 %
GPY2     0 %
GPZ1              10.00 %
GPZ2              10.00 %
P16             1500.00 usec

F1 - Acquisition parameters
TD                  256
SFO1            400.132 MHz
FIDRES        31.300079 Hz
SW               20.025 ppm
FnMODE               QF

F2 - Processing parameters
SI                 4096
SF          400.1300066 MHz
WDW                SINE
SSB      0
LB       0 Hz
GB       0
PC                 1.00

F1 - Processing parameters
SI                  256
MC2                  QF
SF          400.1300000 MHz
WDW              States
SSB      0
LB       0 Hz
GB       0
```

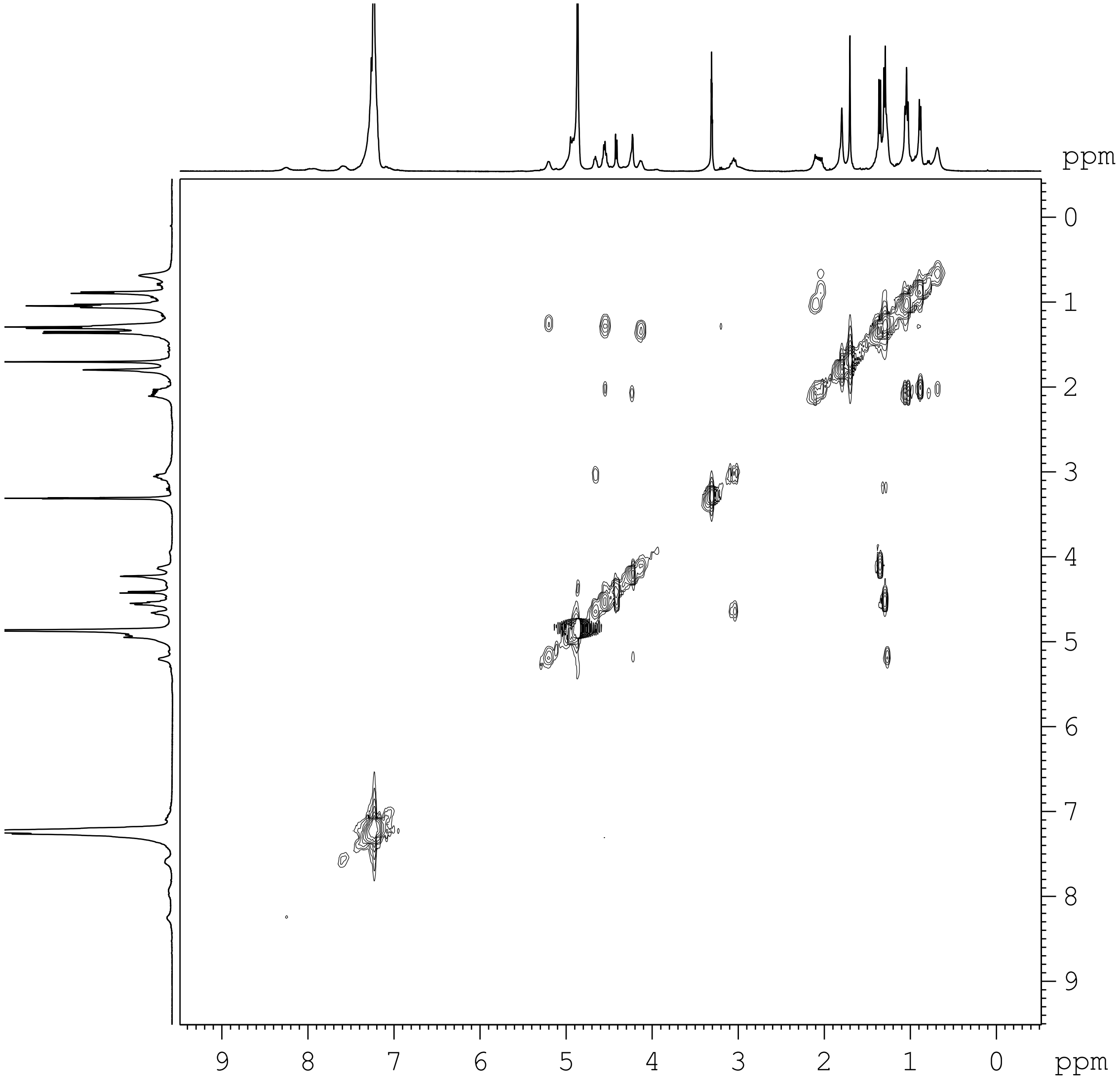

Cyclic depsipeptide (11)

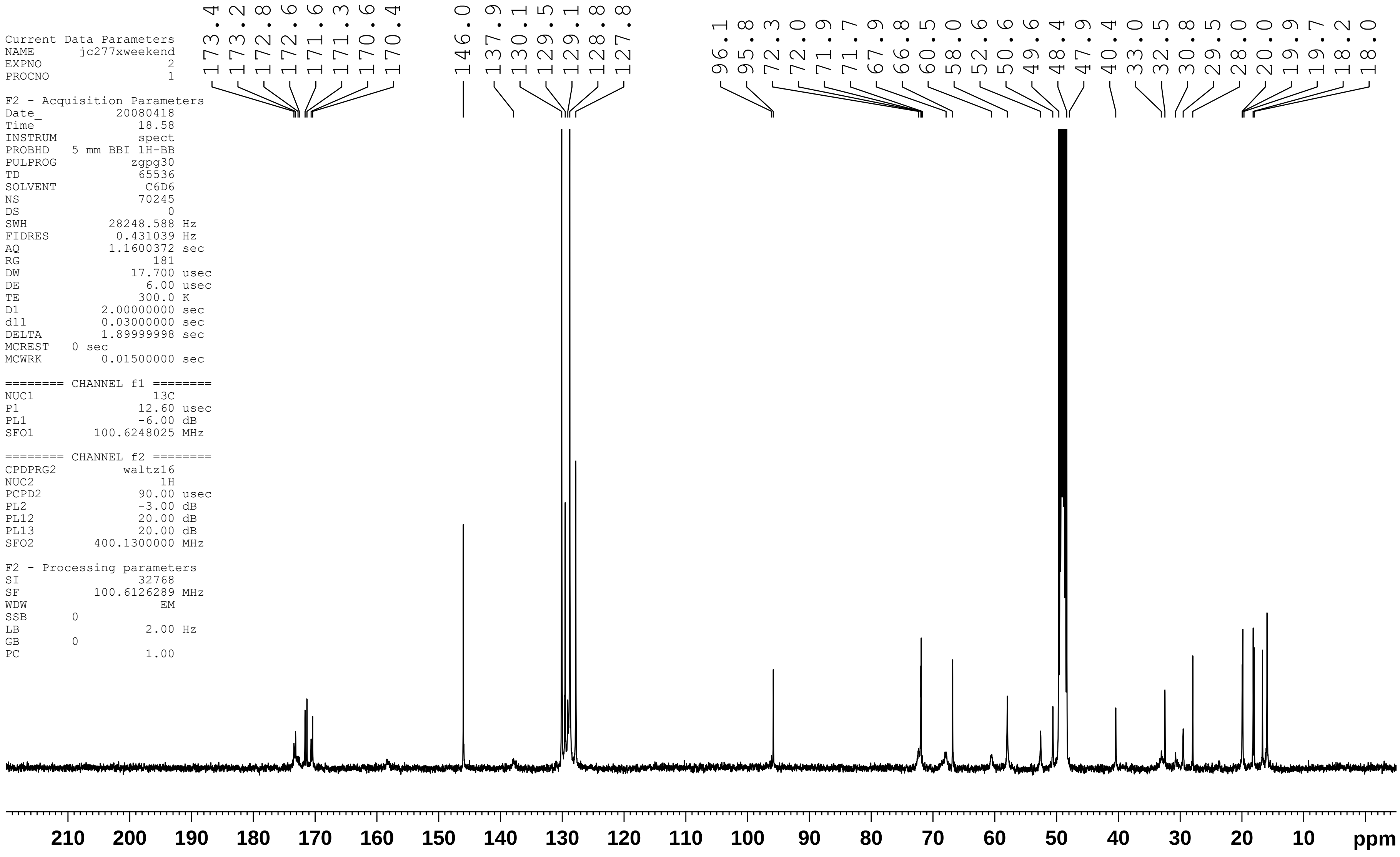

Cyclic depsipeptide (11) HSQC

```
Current Data Parameters
NAME     JC277cyclicpeptide2D
EXPNO                  1
PROCNO                 1

F2 - Acquisition Parameters
Date_          20080312
Time              19.43
INSTRUM           spect
PROBHD   5 mm BBI 1H-BB
PULPROG              zg
TD                32768
SOLVENT            MeOD
NS                   16
DS                    0
SWH            8012.820 Hz
FIDRES         0.244532 Hz
AQ            2.0447731 sec
RG                 71.8
DW               62.400 usec
DE                 6.00 usec
TE                300.0 K
D1           1.00000000 sec
MCREST   0 sec
MCWRK        0.01500000 sec

======== CHANNEL f1 ========
NUC1                 1H
P1                 8.20 usec
PL1               -3.00 dB
SFO1        400.1328009 MHz

F2 - Processing parameters
SI                32768
SF          400.1300066 MHz
WDW                  EM
SSB      0
LB                 0.30 Hz
GB       0
PC                 1.00
```

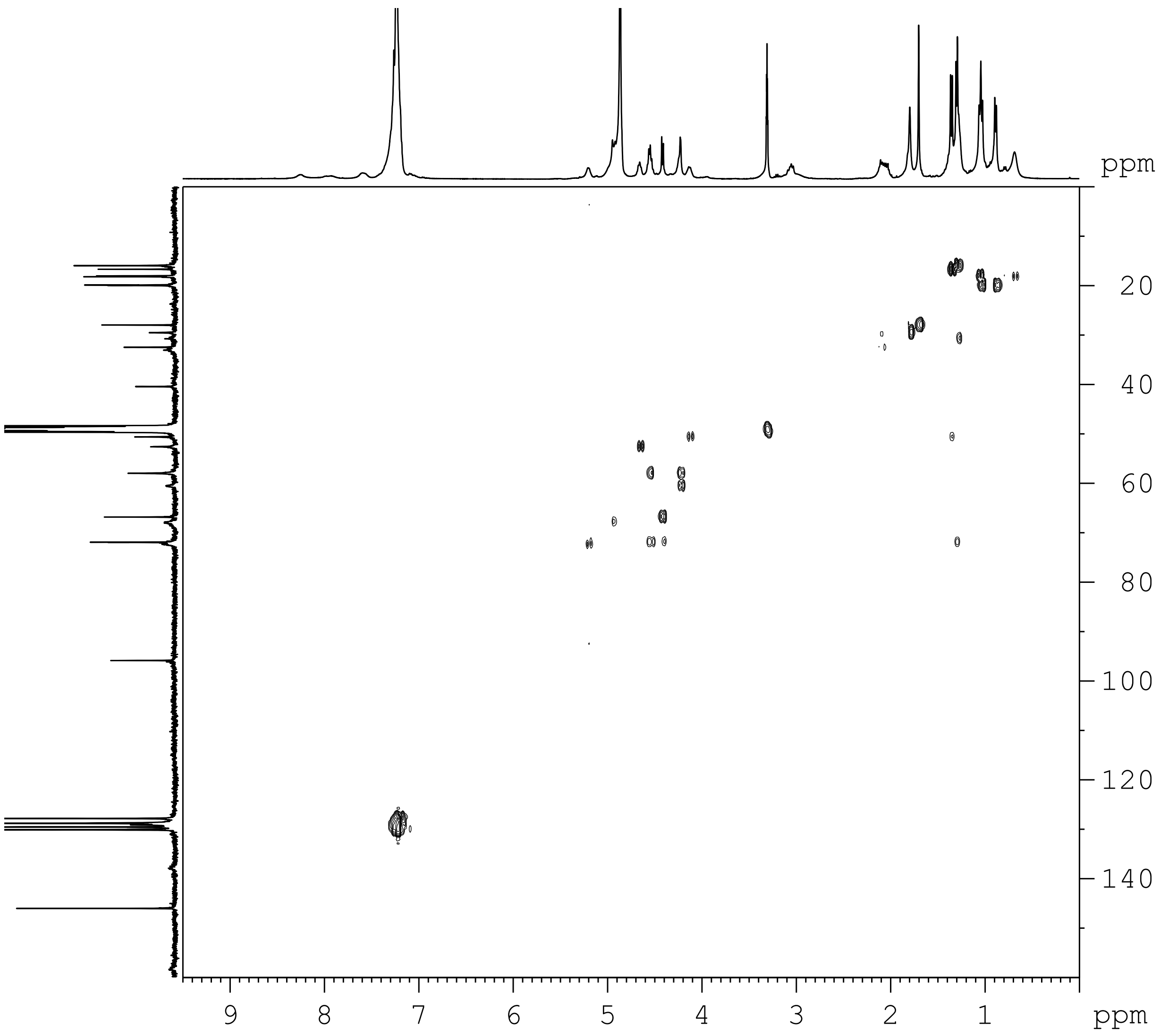

Cyclic depsipeptide (11) HMBC

```
Current Data Parameters
NAME          JC277hsqc
EXPNO                 4
PROCNO                1

F2 - Acquisition Parameters
Date_          20080511
Time              18.07
INSTRUM           spect
PROBHD   5 mm BBI 1H-BB
PULPROG  inv4gplplrndqf
TD                 2048
SOLVENT            MeOH
NS                   64
DS                   16
SWH            8012.820 Hz
FIDRES         3.912510 Hz
AQ            0.1278452 sec
RG                 8192
DW               62.400 usec
DE                 6.00 usec
TE                300.0 K
CNST2       140.0000000
d0           0.00000300 sec
D1           0.80000001 sec
d2           0.00357143 sec
D6           0.07200000 sec
d13          0.00000400 sec
D16          0.00015000 sec
IN0          0.00001988 sec
MCREST   0 sec
MCWRK        0.80000001 sec

======== CHANNEL f1 ========
NUC1                 1H
P1                 8.20 usec
p2                16.40 usec
PL1               -3.00 dB
SFO1        400.1320007 MHz

======== CHANNEL f2 ========
NUC2                13C
P3                12.00 usec
PL2               -6.00 dB
SFO2        100.6227903 MHz

====== GRADIENT CHANNEL =====
GPNAM1         sine.100
GPNAM2         sine.100
GPNAM3         sine.100
GPX1     0 %
GPX2     0 %
GPX3     0 %
GPY1     0 %
GPY2     0 %
GPY3     0 %
GPZ1              50.00 %
GPZ2              30.00 %
GPZ3              40.10 %
P16             1500.00 usec

F1 - Acquisition parameters
TD                  512
SFO1           100.6228 MHz
FIDRES        49.135220 Hz
SW              250.015 ppm
FnMODE               QF

F2 - Processing parameters
SI                 4096
SF          400.1300097 MHz
WDW                SINE
SSB                   2
LB       0 Hz
GB       0
PC                 1.40

F1 - Processing parameters
SI                 2048
MC2                  QF
SF          100.6126243 MHz
WDW              States
SSB                   2
LB       0 Hz
GB       0
```

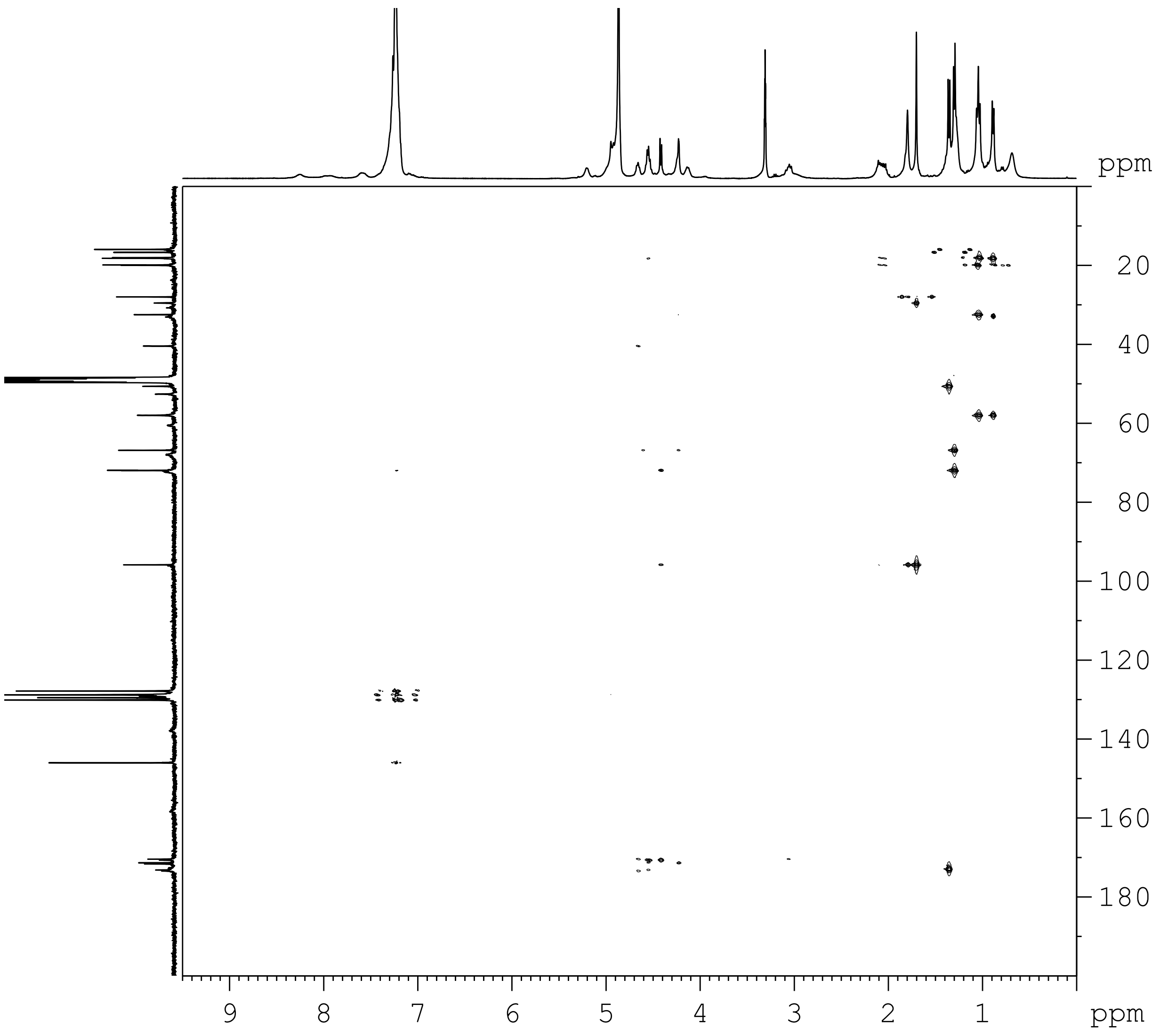

Cyclic depsipeptide (10)

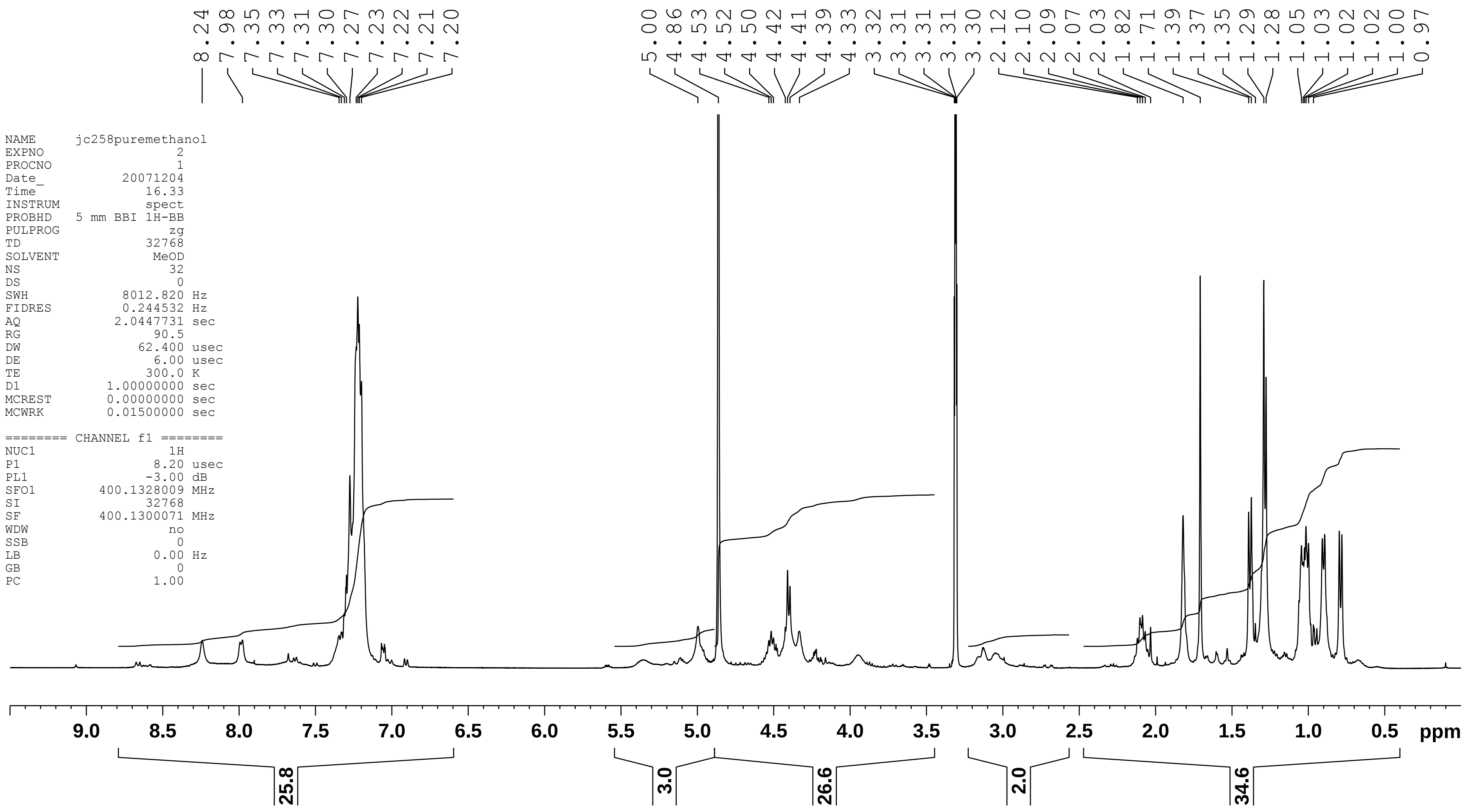

Cyclic depsipeptide (10) COSY

```
Current Data Parameters
NAME          jc258pure
EXPNO                 2
PROCNO                1

F2 - Acquisition Parameters
Date_          20071206
Time              19.20
INSTRUM           spect
PROBHD   5 mm BBI 1H-BB
PULPROG      cosygpmfph
TD                 2048
SOLVENT            MeOH
NS                   15
DS                    0
SWH            4006.410 Hz
FIDRES         1.956255 Hz
AQ            0.2556404 sec
RG               4597.6
DW              124.800 usec
DE                 6.00 usec
TE                300.0 K
d0           0.00011390 sec
D1           2.50000000 sec
d13          0.00000400 sec
D16          0.00015000 sec
d20          0.00165400 sec
IN0          0.00024960 sec
MCREST   0 sec
MCWRK        1.25000000 sec
ST1CNT                0

======== CHANNEL f1 ========
NUC1                 1H
P1                 8.56 usec
p2                17.12 usec
PL1               -3.00 dB
SFO1        400.1320007 MHz

====== GRADIENT CHANNEL =====
GPNAM1         sine.100
GPNAM2         sine.100
GPX1     0 %
GPX2     0 %
GPY1     0 %
GPY2     0 %
GPZ1              10.00 %
GPZ2              20.00 %
P16             1500.00 usec

F1 - Acquisition parameters
TD                  256
SFO1            400.132 MHz
FIDRES        15.650040 Hz
SW               10.013 ppm
FnMODE      States-TPPI

F2 - Processing parameters
SI                 2048
SF          400.1300035 MHz
WDW                SINE
SSB      0
LB       0 Hz
GB       0
PC                 1.00

F1 - Processing parameters
SI                  256
MC2         States-TPPI
SF          400.1299891 MHz
WDW
SSB      0
LB       0 Hz
GB       0
```

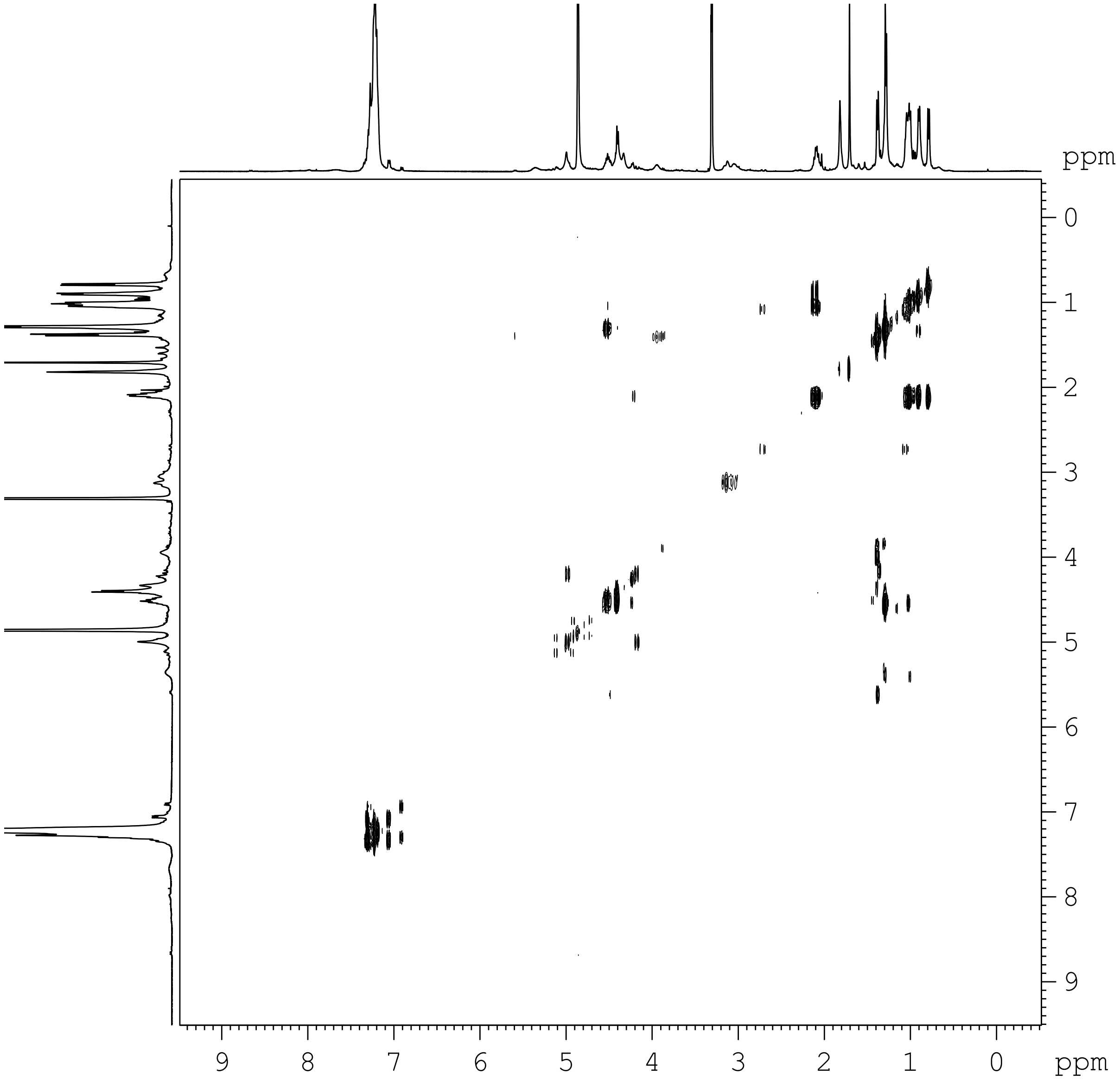

Cyclic depsipeptide (10) HMBC

```
Current Data Parameters
NAME      JC493pseudo1b
EXPNO                 2
PROCNO                1

F2 - Acquisition Parameters
Date_          20100728
Time              18.19
INSTRUM           spect
PROBHD   5 mm BBI 1H-BB
PULPROG  inv4gplplrndqf
TD                 4096
SOLVENT           CD3CN
NS                   64
DS                    8
SWH            4006.410 Hz
FIDRES         0.978127 Hz
AQ            0.5112308 sec
RG                 2048
DW              124.800 usec
DE                 6.00 usec
TE       0 K
CNST2       145.0000000
d0           0.00000300 sec
D1           1.50000000 sec
d2           0.00344828 sec
D6           0.06250000 sec
d13          0.00000400 sec
D16          0.00015000 sec
IN0          0.00002484 sec
MCREST   0 sec
MCWRK        1.50000000 sec

======== CHANNEL f1 ========
NUC1                 1H
P1                 6.07 usec
p2                12.14 usec
PL1               -3.00 dB
SFO1        400.2101790 MHz

======== CHANNEL f2 ========
NUC2                13C
P3                12.00 usec
PL2               -6.00 dB
SFO2        100.6424892 MHz

====== GRADIENT CHANNEL =====
GPNAM1         sine.100
GPNAM2         sine.100
GPNAM3         sine.100
GPX1     0 %
GPX2     0 %
GPX3     0 %
GPY1     0 %
GPY2     0 %
GPY3     0 %
GPZ1              50.00 %
GPZ2              30.00 %
GPZ3              40.10 %
P16             1500.00 usec

F1 - Acquisition parameters
TD                  512
SFO1           100.6425 MHz
FIDRES        39.318066 Hz
SW              200.023 ppm
FnMODE               QF

F2 - Processing parameters
SI                 2048
SF          400.2081726 MHz
WDW                SINE
SSB                   2
LB       0 Hz
GB       0
PC                 1.00

F1 - Processing parameters
SI                 1024
MC2                  QF
SF          100.6323106 MHz
WDW              States
SSB                   2
LB       0 Hz
GB       0
```

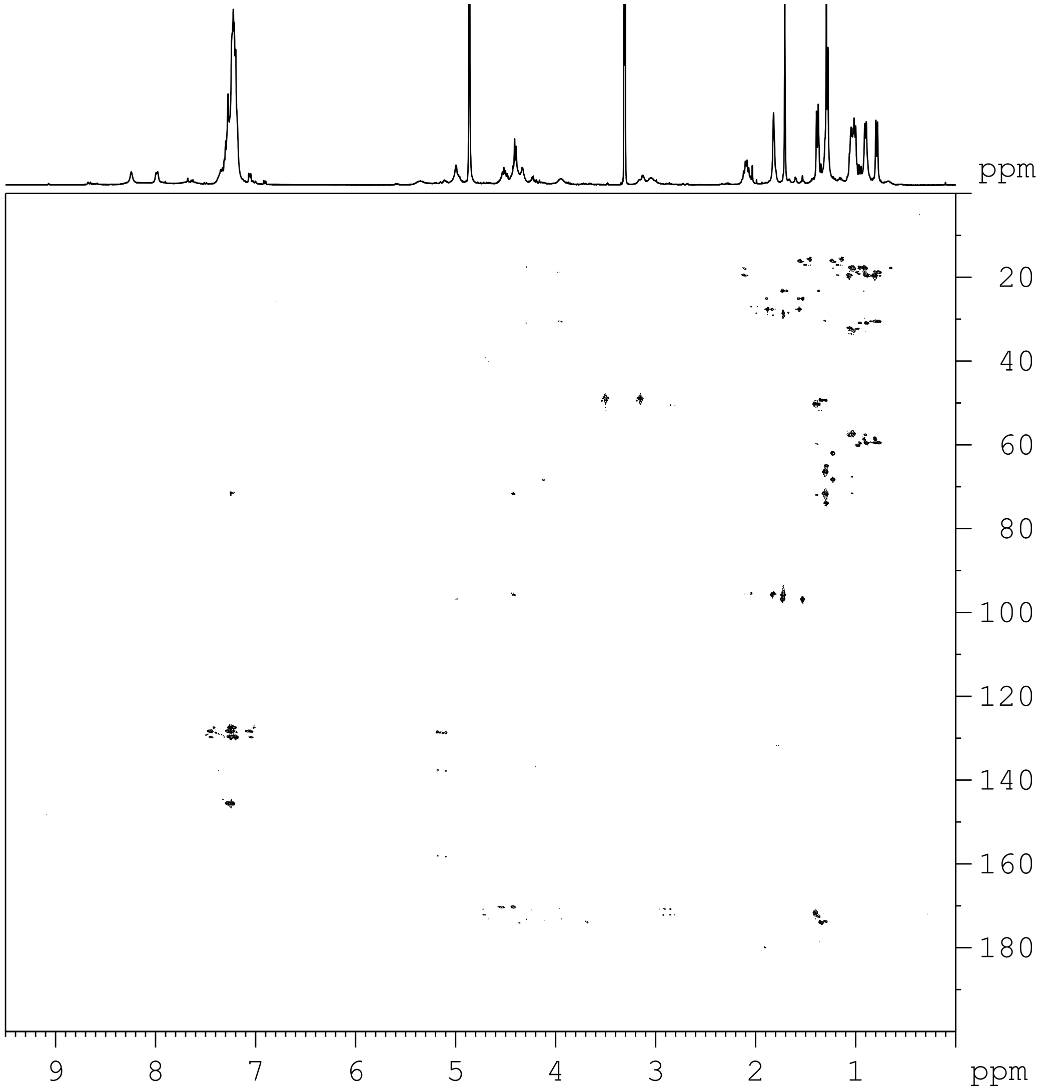

Cbz-L-Thr-D-Val-L-Val-D-*allo*-Thr(*tert*-butyl)-D-Asn(Trt)-L-Ala-OH (9)

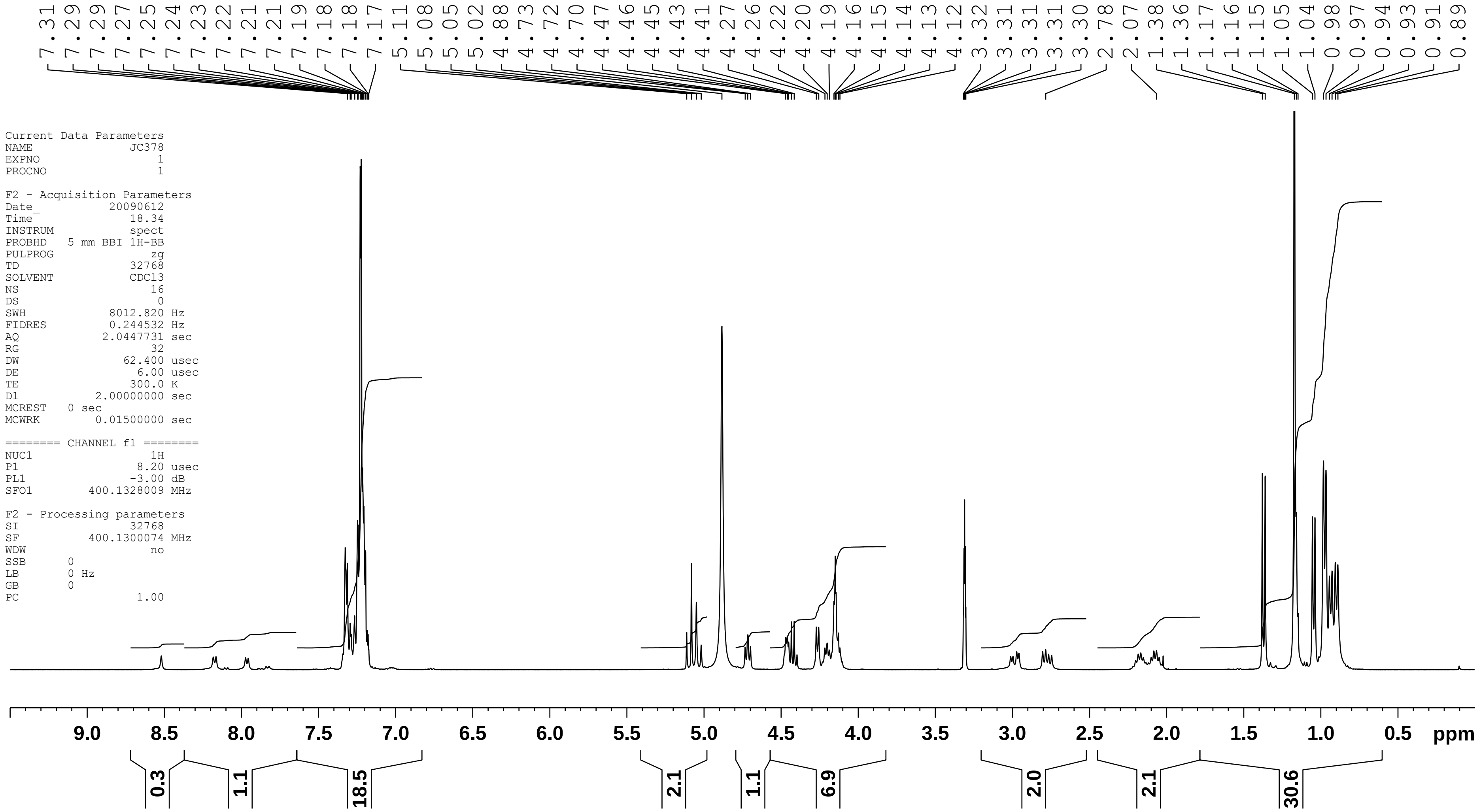

Cbz-L-Thr-D-Val-L-Val-D-*allo*-Thr(*tert*-butyl)-D-Asn(Trt)-L-Ala-OH (9) COSY

```
Current Data Parameters
NAME                JC378
EXPNO                   3
PROCNO                  1

F2 - Acquisition Parameters
Date_            20090612
Time                21.54
INSTRUM             spect
PROBHD   5 mm BBI 1H-BB
PULPROG      cosygpmfph
TD                   4096
SOLVENT              MeOH
NS                     16
DS                      8
SWH              4006.410 Hz
FIDRES           0.978127 Hz
AQ              0.5112308 sec
RG                1448.15
DW                124.800 usec
DE                   6.00 usec
TE                  300.0 K
d0             0.00011452 sec
D1             3.00000000 sec
d13            0.00000400 sec
D16            0.00015000 sec
d20            0.00165400 sec
IN0            0.00024991 sec
MCREST   0 sec
MCWRK          1.50000000 sec
ST1CNT                  0

======== CHANNEL f1 ========
NUC1                   1H
P1                   8.20 usec
p2                  16.40 usec
PL1                 -3.00 dB
SFO1          400.1318006 MHz

====== GRADIENT CHANNEL =====
GPNAM1           sine.100
GPNAM2           sine.100
GPX1     0 %
GPX2     0 %
GPY1     0 %
GPY2     0 %
GPZ1                10.00 %
GPZ2                20.00 %
P16               1500.00 usec

F1 - Acquisition parameters
TD                    256
SFO1             400.1318 MHz
FIDRES          15.630470 Hz
SW                 10.000 ppm
FnMODE        States-TPPI

F2 - Processing parameters
SI                   4096
SF            400.1300076 MHz
WDW                  SINE
SSB                     2
LB       0 Hz
GB       0
PC                   1.00

F1 - Processing parameters
SI                   1024
MC2           States-TPPI
SF            400.1300076 MHz
WDW
SSB                     2
LB       0 Hz
GB       0
```

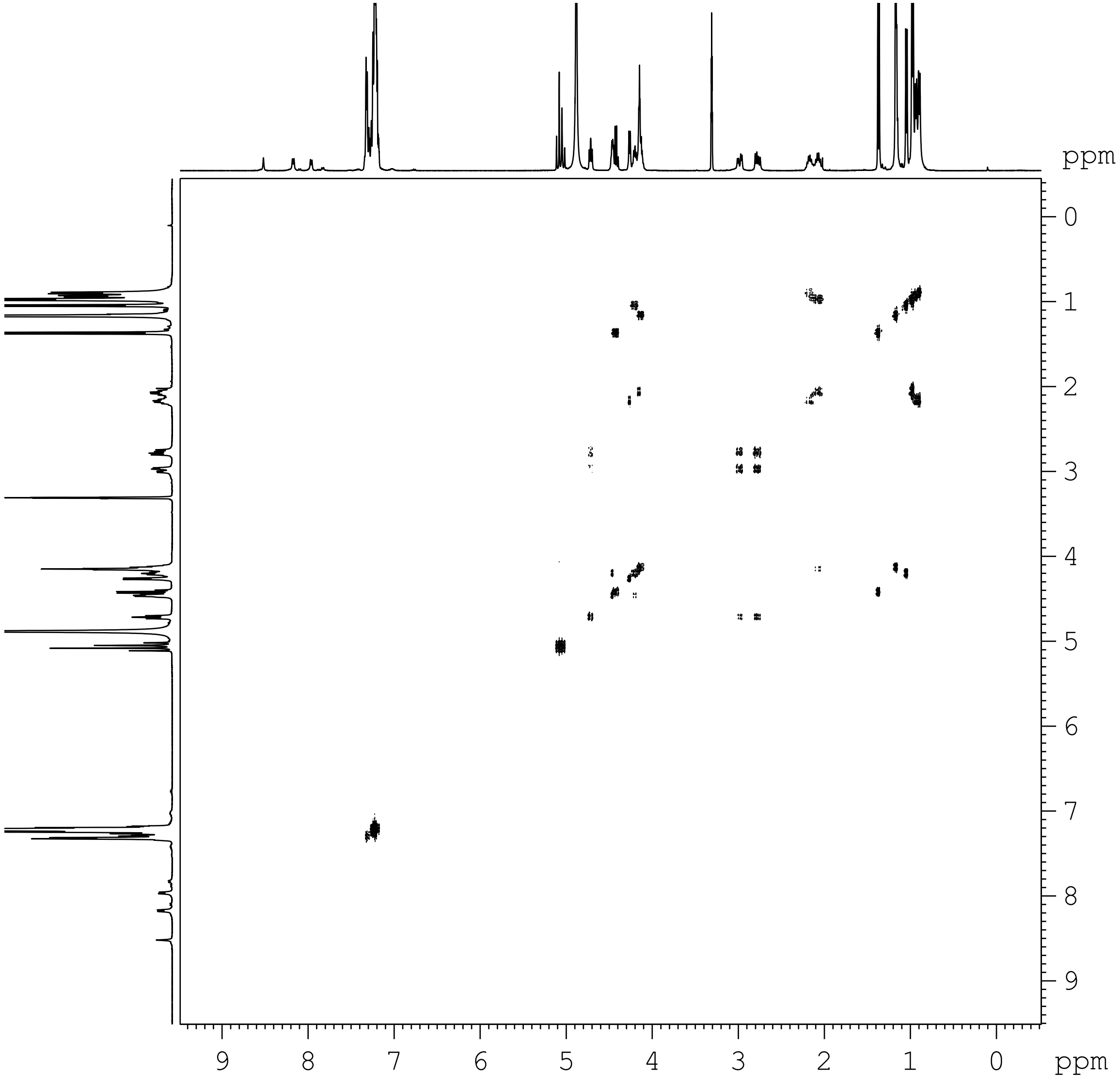

Cbz-L-Thr-D-Val-L-Val-D-*allo*-Thr(*tert*-butyl)-D-Asn(Trt)-L-Ala-OH (9)

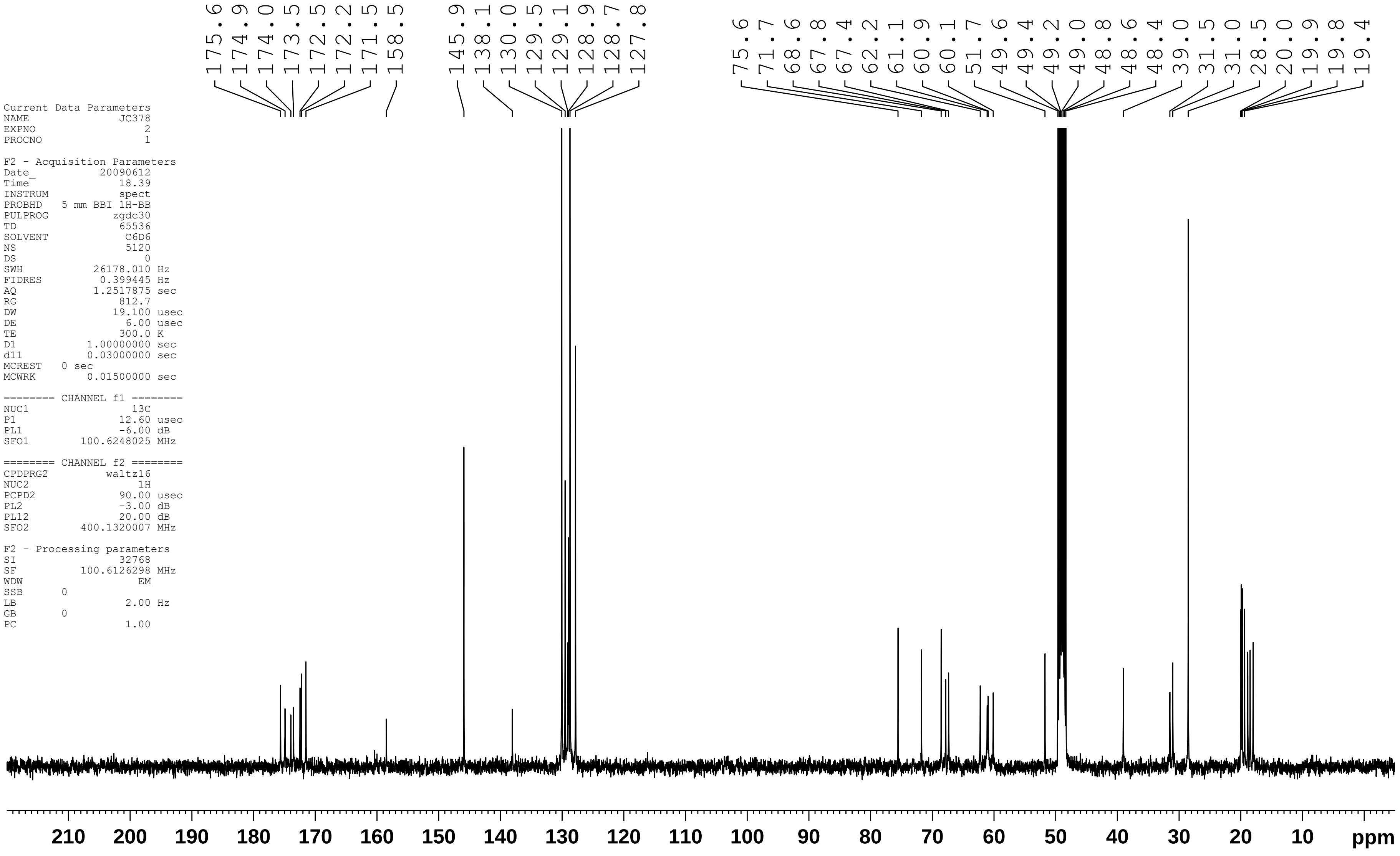

Cbz-L-Thr-D-Val-L-Val-D-*allo*-Thr(*tert*-butyl)-D-Asn(Trt)-L-Ala-OH (9) HSQC

```
Current Data Parameters
NAME               JC378
EXPNO                  5
PROCNO                 1

F2 - Acquisition Parameters
Date_           20090613
Time                6.53
INSTRUM            spect
PROBHD   5 mm BBI 1H-BB
PULPROG       invietgpsi
TD                  4096
SOLVENT            CDCl3
NS                    16
DS                     8
SWH             4006.410 Hz
FIDRES          0.978127 Hz
AQ             0.5112308 sec
RG                  2048
DW               124.800 usec
DE                  6.00 usec
TE                 300.0 K
CNST2        140.0000000
d0            0.00000300 sec
D1            3.00000000 sec
d4            0.00178571 sec
d11           0.03000000 sec
d13           0.00000400 sec
D16           0.00015000 sec
D24           0.00178500 sec
DELTA         0.00172240 sec
DELTA1        0.00165800 sec
IN0           0.00002259 sec
MCREST   0 sec
MCWRK         0.50000101 sec
ST1CNT                 0

======== CHANNEL f1 ========
NUC1                  1H
P1                  8.20 usec
p2                 16.40 usec
P28                 0.50 usec
PL1                -3.00 dB
SFO1         400.1318006 MHz

======== CHANNEL f2 ========
CPDPRG2             garp
NUC2                 13C
P3                 12.00 usec
p4                 24.00 usec
PCPD2              70.00 usec
PL2                -6.00 dB
PL12               10.70 dB
SFO2         100.6227903 MHz

====== GRADIENT CHANNEL =====
GPNAM1          sine.100
GPNAM2          sine.100
GPX1     0 %
GPX2     0 %
GPY1     0 %
GPY2     0 %
GPZ1               80.00 %
GPZ2               20.10 %
P16              1500.00 usec

F1 - Acquisition parameters
TD                   256
SFO1            100.6228 MHz
FIDRES         86.469284 Hz
SW               219.991 ppm
FnMODE    Echo-Antiecho

F2 - Processing parameters
SI                  2048
SF           400.1300049 MHz
WDW                 SINE
SSB                    2
LB       0 Hz
GB       0
PC                  1.40

F1 - Processing parameters
SI                   512
MC2       echo-antiecho
SF           100.6126295 MHz
WDW
SSB                    1
LB       0 Hz
GB       0
```

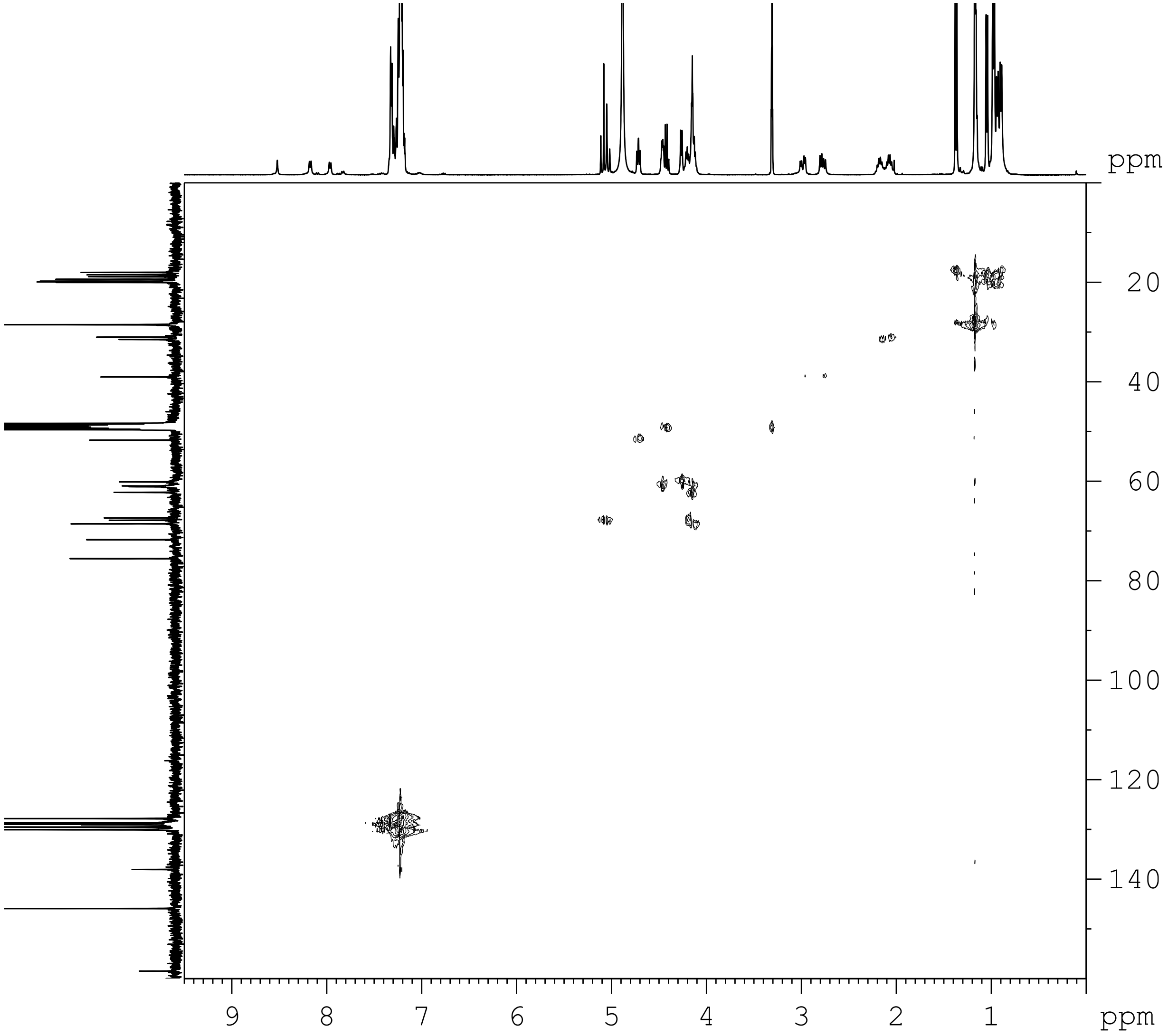

Cbz-L-Thr-D-Val-L-Val-D-*allo*-Thr(*tert*-butyl)-D-Asn(Trt)-L-Ala-OH (9) HMBC

```
Current Data Parameters
NAME                JC378
EXPNO                   6
PROCNO                  1

F2 - Acquisition Parameters
Date_            20090613
Time                10.56
INSTRUM             spect
PROBHD   5 mm BBI 1H-BB
PULPROG  inv4gplplrndqf
TD                   4096
SOLVENT              MeOH
NS                     32
DS                      8
SWH              4006.410 Hz
FIDRES           0.978127 Hz
AQ              0.5112308 sec
RG                   2048
DW                124.800 usec
DE                   6.00 usec
TE                  300.0 K
CNST2         140.0000000
d0             0.00000300 sec
D1             3.00000000 sec
d2             0.00357143 sec
D6             0.07200000 sec
d13            0.00000400 sec
D16            0.00015000 sec
IN0            0.00002259 sec
MCREST   0 sec
MCWRK          3.00000000 sec

======== CHANNEL f1 ========
NUC1                   1H
P1                   8.20 usec
p2                  16.40 usec
PL1                 -3.00 dB
SFO1          400.1318006 MHz

======== CHANNEL f2 ========
NUC2                  13C
P3                  12.00 usec
PL2                 -6.00 dB
SFO2          100.6227903 MHz

====== GRADIENT CHANNEL =====
GPNAM1           sine.100
GPNAM2           sine.100
GPNAM3           sine.100
GPX1     0 %
GPX2     0 %
GPX3     0 %
GPY1     0 %
GPY2     0 %
GPY3     0 %
GPZ1                50.00 %
GPZ2                30.00 %
GPZ3                40.10 %
P16               1500.00 usec

F1 - Acquisition parameters
TD                    129
SFO1             100.6228 MHz
FIDRES         171.597961 Hz
SW                219.991 ppm
FnMODE                 QF

F2 - Processing parameters
SI                   1024
SF            400.1300076 MHz
WDW                  SINE
SSB                     2
LB       0 Hz
GB       0
PC                   1.40

F1 - Processing parameters
SI                    512
MC2                    QF
SF            100.6126298 MHz
WDW                States
SSB                     2
LB       0 Hz
GB       0
```

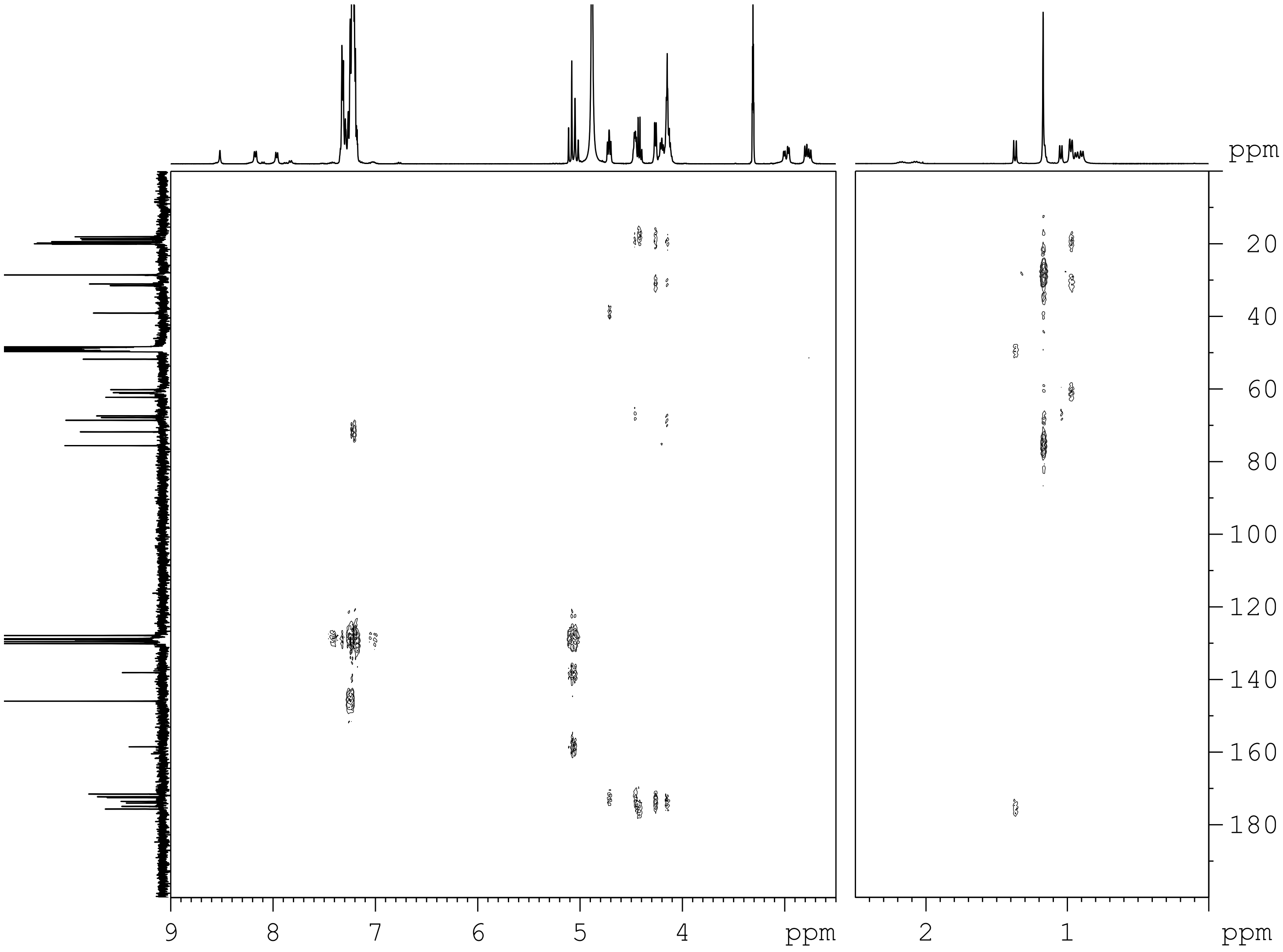

Cyclic depsipeptide (8)

```
Current Data Parameters
NAME              jc375
EXPNO                 1
PROCNO                1

F2 - Acquisition Parameters
Date_          20090529
Time              17.43
INSTRUM           spect
PROBHD   5 mm BBI 1H-BB
PULPROG              zg
TD                32768
SOLVENT            MeOD
NS                   32
DS                    0
SWH            8012.820 Hz
FIDRES         0.244532 Hz
AQ            2.0447731 sec
RG                 35.9
DW               62.400 usec
DE                 6.00 usec
TE                300.0 K
D1           2.00000000 sec
MCREST   0 sec
MCWRK        0.01500000 sec

======== CHANNEL f1 ========
NUC1                 1H
P1                 8.20 usec
PL1               -3.00 dB
SFO1        400.1328009 MHz

F2 - Processing parameters
SI                32768
SF          400.1300073 MHz
WDW                  no
SSB      0
LB       0 Hz
GB       0
PC                 1.00
```

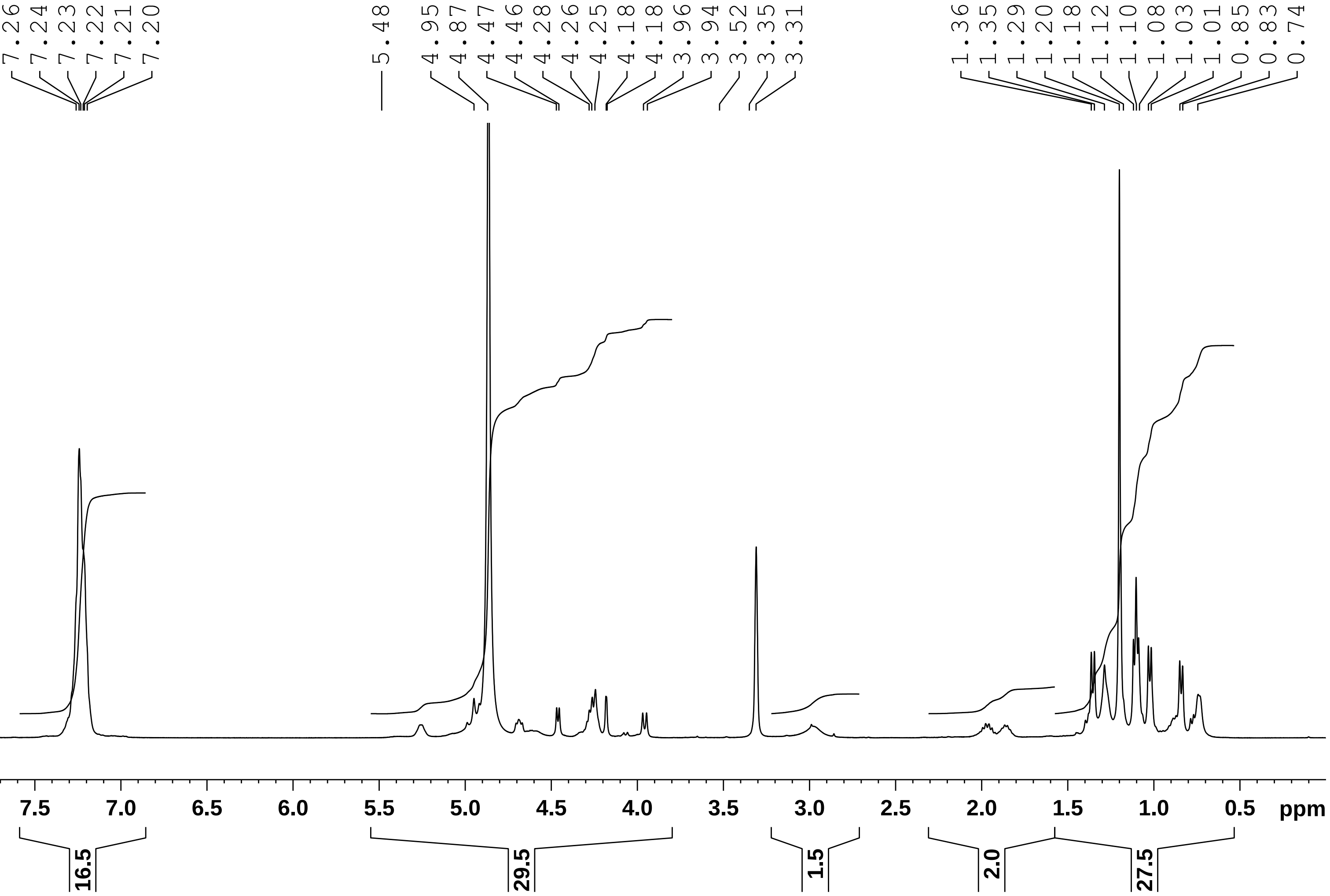

Cyclic depsipeptide (8) COSY

```
Current Data Parameters
NAME              jc375
EXPNO                 5
PROCNO                1

F2 - Acquisition Parameters
Date_          20090531
Time              16.54
INSTRUM           spect
PROBHD   5 mm BBI 1H-BB
PULPROG      cosygpmfph
TD                 4096
SOLVENT            MeOD
NS                   16
DS                    8
SWH            4006.410 Hz
FIDRES         0.978127 Hz
AQ            0.5112308 sec
RG               812.75
DW              124.800 usec
DE                 6.00 usec
TE                300.0 K
d0           0.00011174 sec
D1           2.50000000 sec
d13          0.00000400 sec
D16          0.00015000 sec
d20          0.00165400 sec
IN0          0.00024991 sec
MCREST   0 sec
MCWRK        1.25000000 sec
ST1CNT                0

======== CHANNEL f1 ========
NUC1                 1H
P1                10.38 usec
p2                20.76 usec
PL1               -3.00 dB
SFO1        400.1320007 MHz

====== GRADIENT CHANNEL =====
GPNAM1         sine.100
GPNAM2         sine.100
GPX1     0 %
GPX2     0 %
GPY1     0 %
GPY2     0 %
GPZ1              10.00 %
GPZ2              20.00 %
P16             1500.00 usec

F1 - Acquisition parameters
TD                  256
SFO1            400.132 MHz
FIDRES        15.630470 Hz
SW               10.000 ppm
FnMODE      States-TPPI

F2 - Processing parameters
SI                 4096
SF          400.1300051 MHz
WDW                SINE
SSB                   2
LB       0 Hz
GB       0
PC                 1.00

F1 - Processing parameters
SI                 1024
MC2         States-TPPI
SF          400.1300089 MHz
WDW
SSB                   2
LB       0 Hz
GB       0
```

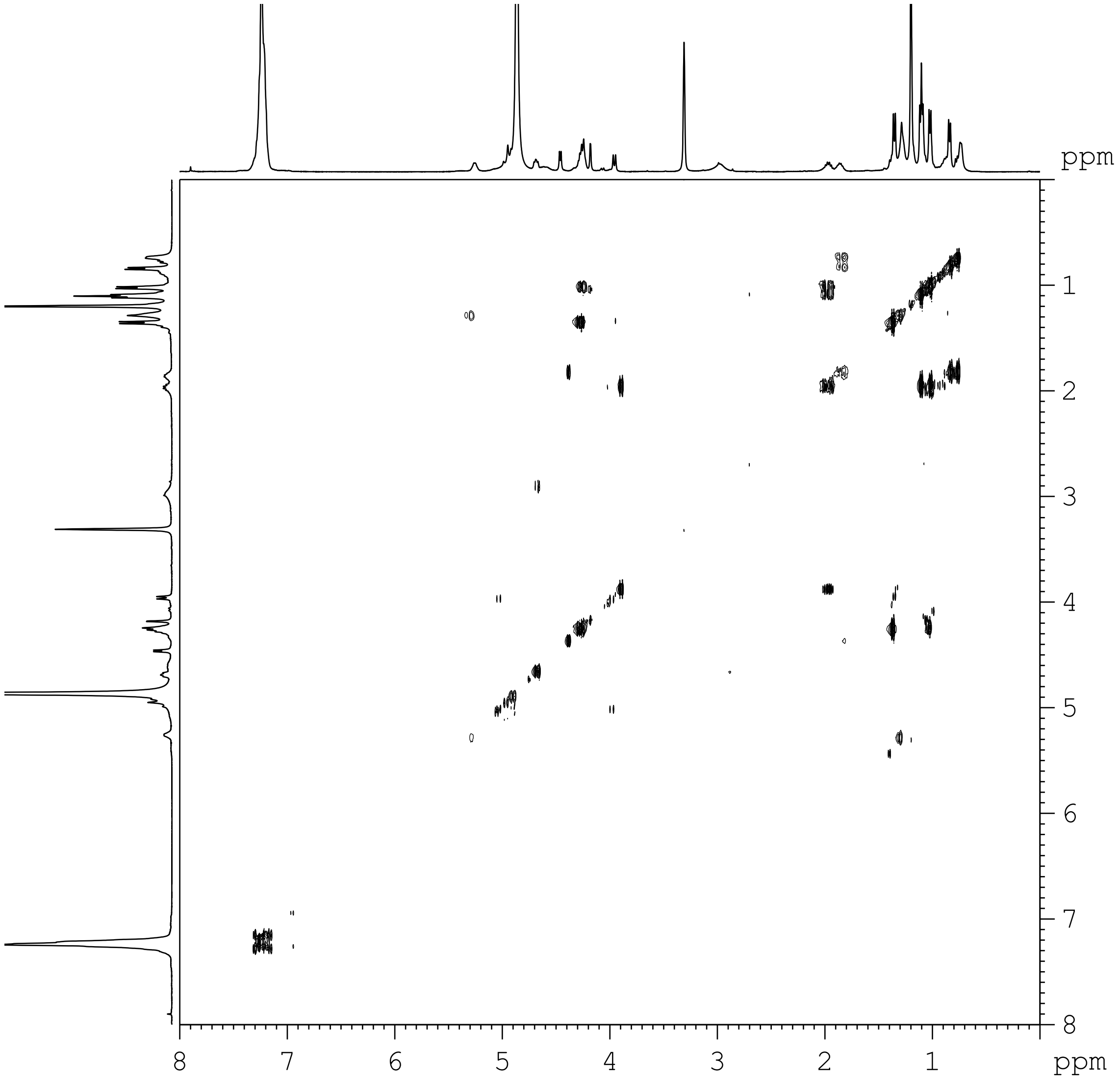

Cyclic depsipeptide (8)

```
Current Data Parameters
NAME              jc375
EXPNO                 2
PROCNO                1

F2 - Acquisition Parameters
Date_          20090529
Time              17.46
INSTRUM           spect
PROBHD   5 mm BBI 1H-BB
PULPROG         zgdc30
TD                65536
SOLVENT            C6D6
NS                20480
DS                    0
SWH           26178.010 Hz
FIDRES         0.399445 Hz
AQ            1.2517875 sec
RG                 90.5
DW               19.100 usec
DE                 6.00 usec
TE                300.0 K
D1           1.00000000 sec
d11          0.03000000 sec
MCREST   0 sec
MCWRK        0.01500000 sec

======== CHANNEL f1 ========
NUC1                13C
P1                12.60 usec
PL1               -6.00 dB
SFO1        100.6248025 MHz

======== CHANNEL f2 ========
CPDPRG2         waltz16
NUC2                 1H
PCPD2             90.00 usec
PL2               -3.00 dB
PL12              20.00 dB
SFO2        400.1320007 MHz

F2 - Processing parameters
SI                32768
SF          100.6126307 MHz
WDW                  EM
SSB      0
LB                 2.00 Hz
GB       0
PC                 1.00
```

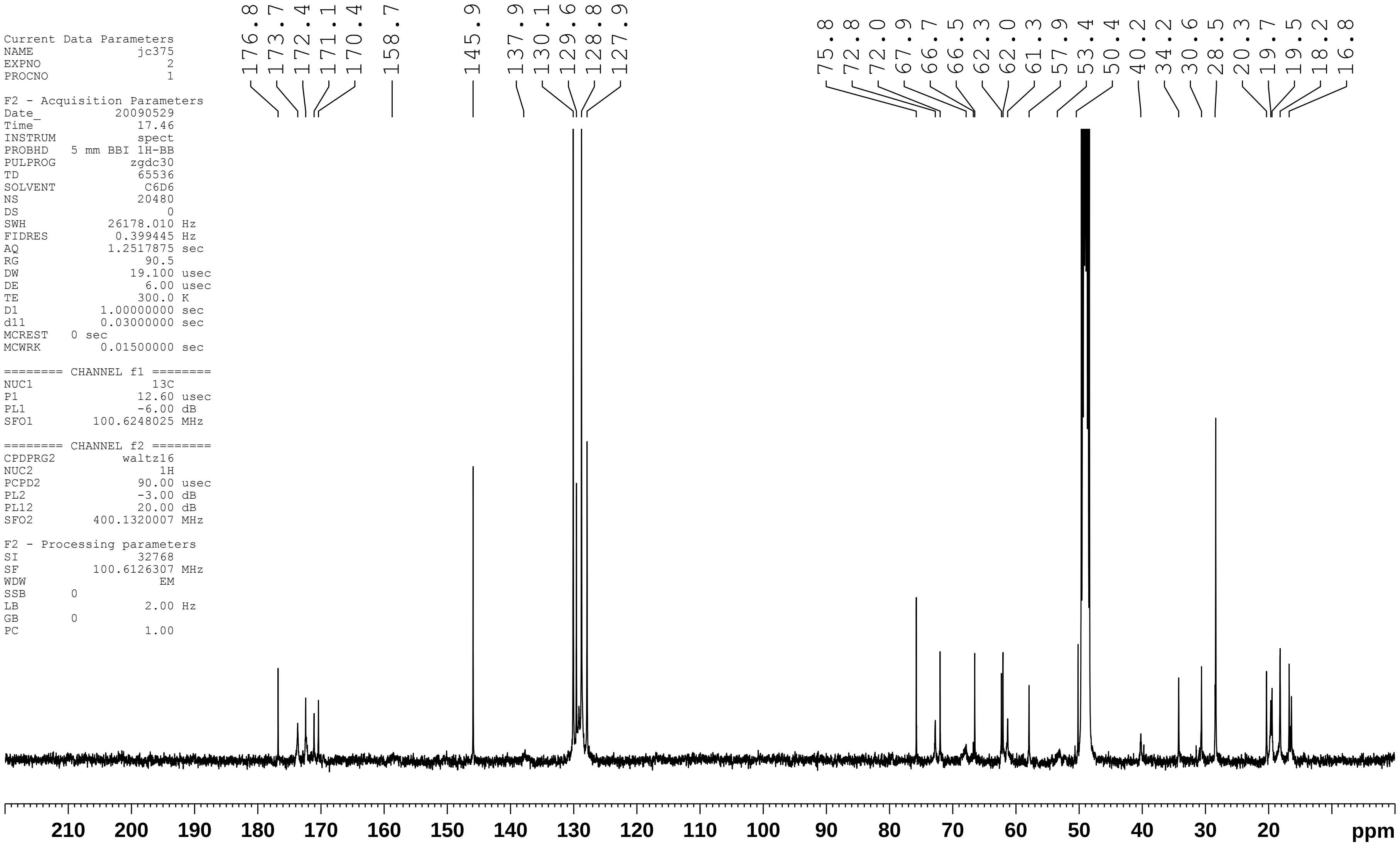

Cyclic depsipeptide (8) HSQC

```
Current Data Parameters
NAME               jc375
EXPNO                  7
PROCNO                 1

F2 - Acquisition Parameters
Date_           20090601
Time                0.45
INSTRUM            spect
PROBHD   5 mm BBI 1H-BB
PULPROG       invietgpsi
TD                  4096
SOLVENT            CDCl3
NS                    32
DS                     8
SWH             4006.410 Hz
FIDRES          0.978127 Hz
AQ             0.5112308 sec
RG                2298.8
DW               124.800 usec
DE                  6.00 usec
TE                 300.0 K
CNST2        140.0000000
d0            0.00000300 sec
D1            2.50000000 sec
d4            0.00178571 sec
d11           0.03000000 sec
d13           0.00000400 sec
D16           0.00015000 sec
D24           0.00178500 sec
DELTA         0.00172676 sec
DELTA1        0.00165800 sec
IN0           0.00002259 sec
MCREST   0 sec
MCWRK         0.41666749 sec
ST1CNT                 0

======== CHANNEL f1 ========
NUC1                  1H
P1                 10.38 usec
p2                 20.76 usec
P28                 0.50 usec
PL1                -3.00 dB
SFO1         400.1320007 MHz

======== CHANNEL f2 ========
CPDPRG2             garp
NUC2                 13C
P3                 12.00 usec
p4                 24.00 usec
PCPD2              70.00 usec
PL2                -6.00 dB
PL12               10.70 dB
SFO2         100.6227903 MHz

====== GRADIENT CHANNEL =====
GPNAM1          sine.100
GPNAM2          sine.100
GPX1     0 %
GPX2     0 %
GPY1     0 %
GPY2     0 %
GPZ1               80.00 %
GPZ2               20.10 %
P16              1500.00 usec

F1 - Acquisition parameters
TD                   256
SFO1            100.6228 MHz
FIDRES         86.469284 Hz
SW               219.991 ppm
FnMODE    Echo-Antiecho

F2 - Processing parameters
SI                  2048
SF           400.1300067 MHz
WDW                 SINE
SSB                    2
LB       0 Hz
GB       0
PC                  1.40

F1 - Processing parameters
SI                  1024
MC2       echo-antiecho
SF           100.6126976 MHz
WDW
SSB                    2
LB       0 Hz
GB       0
```

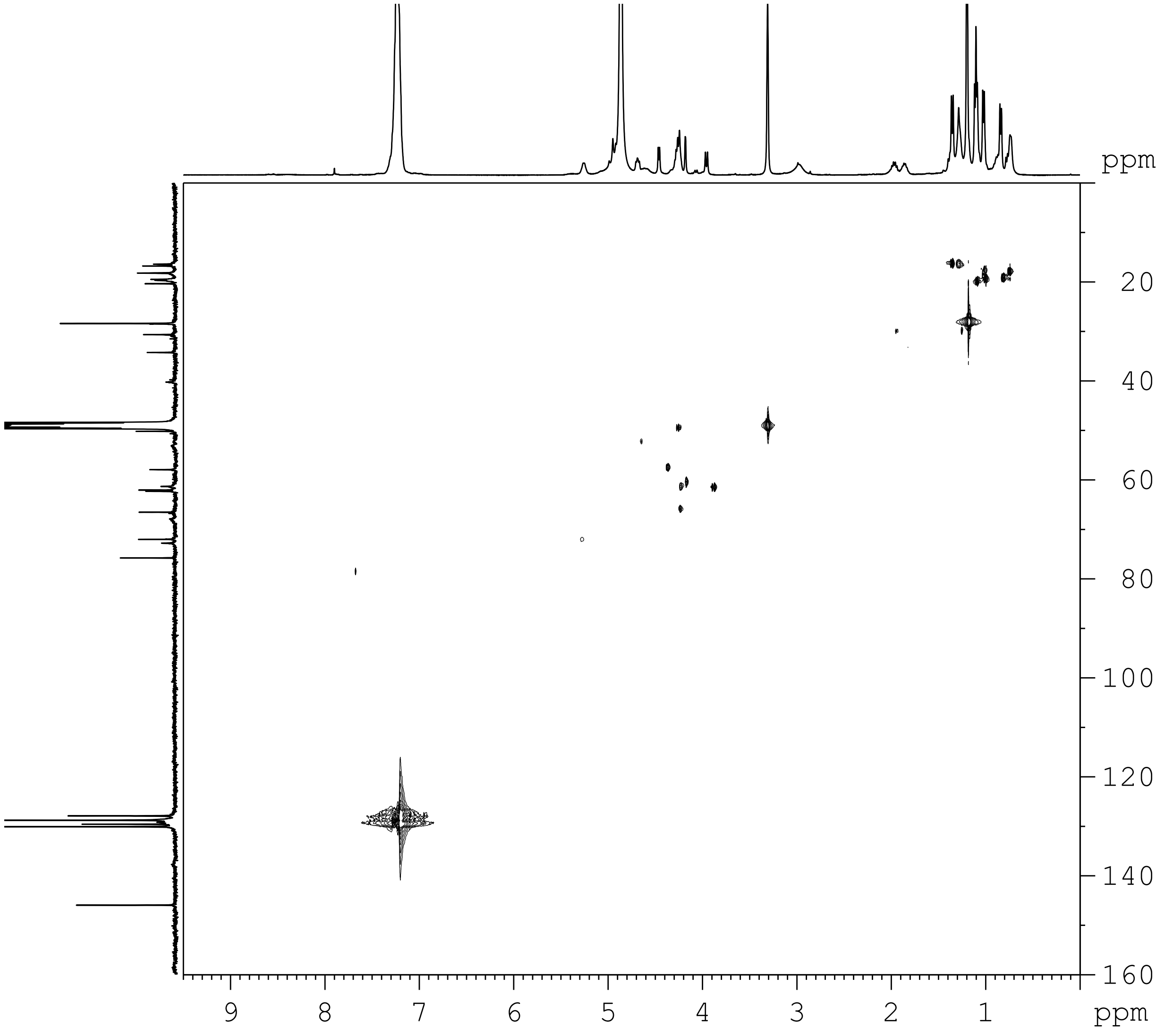

Cyclic depsipeptide (17)

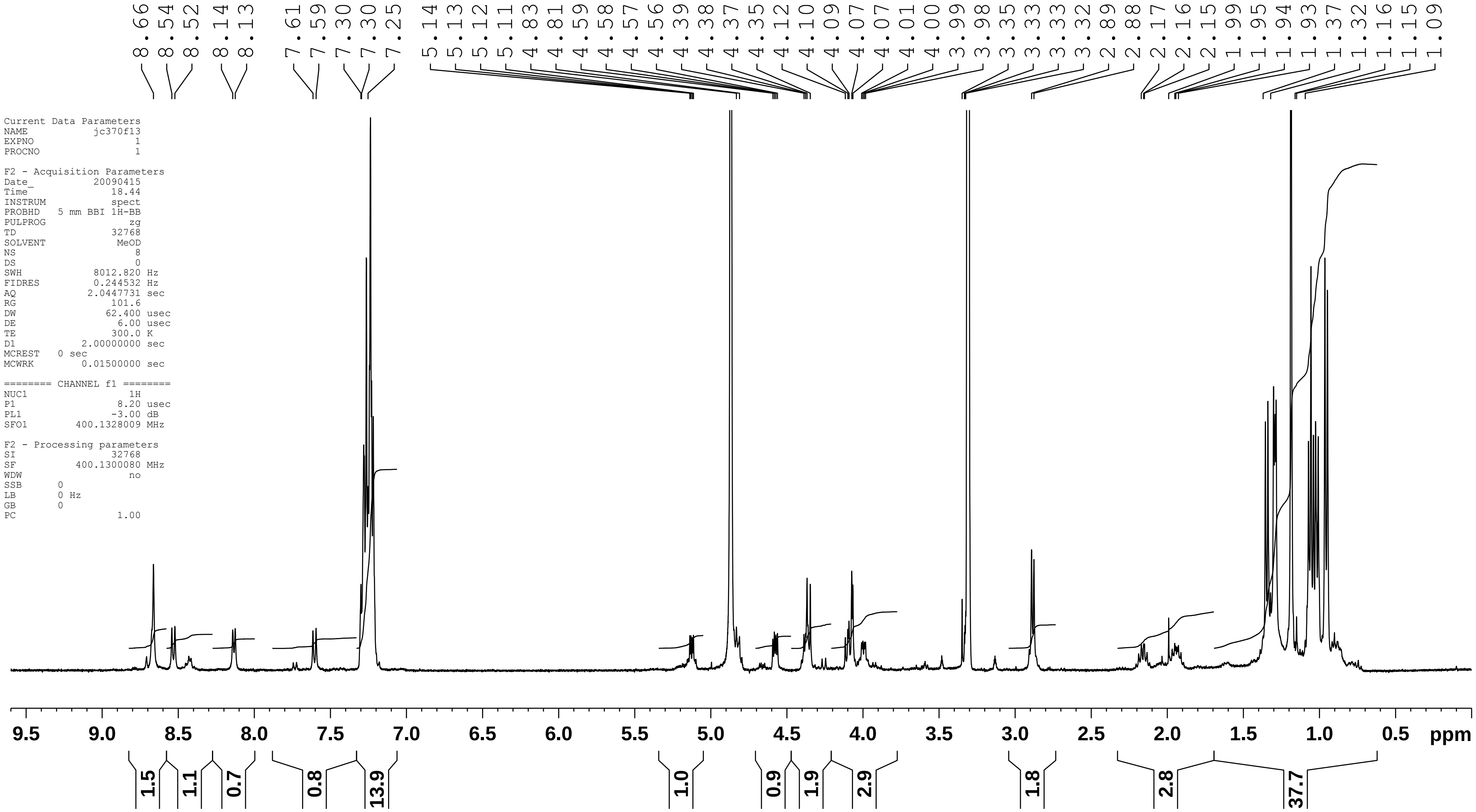

Cyclic depsipeptide (17) COSY

```
Current Data Parameters
NAME          JC377hsqc
EXPNO                 2
PROCNO                1

F2 - Acquisition Parameters
Date_          20090626
Time              18.37
INSTRUM           spect
PROBHD   5 mm BBI 1H-BB
PULPROG      cosygpmfph
TD                 4096
SOLVENT            MeOH
NS                   48
DS                    8
SWH            2394.636 Hz
FIDRES         0.584628 Hz
AQ            0.8552948 sec
RG              645.075
DW              208.800 usec
DE                 6.00 usec
TE                300.0 K
d0           0.00019712 sec
D1           1.29999995 sec
d13          0.00000400 sec
D16          0.00015000 sec
d20          0.00165400 sec
IN0          0.00041652 sec
MCREST   0 sec
MCWRK        0.64999998 sec
ST1CNT                0

======== CHANNEL f1 ========
NUC1                 1H
P1                 8.75 usec
p2                17.50 usec
PL1               -3.00 dB
SFO1        400.1312004 MHz

====== GRADIENT CHANNEL =====
GPNAM1         sine.100
GPNAM2         sine.100
GPX1     0 %
GPX2     0 %
GPY1     0 %
GPY2     0 %
GPZ1              10.00 %
GPZ2              20.00 %
P16             1500.00 usec

F1 - Acquisition parameters
TD                  256
SFO1           400.1312 MHz
FIDRES         9.378188 Hz
SW                6.000 ppm
FnMODE      States-TPPI

F2 - Processing parameters
SI                 2048
SF          400.1300078 MHz
WDW                SINE
SSB      0
LB       0 Hz
GB       0
PC                 1.00

F1 - Processing parameters
SI                  256
MC2         States-TPPI
SF          400.1300078 MHz
WDW
SSB      0
LB       0 Hz
GB       0
```

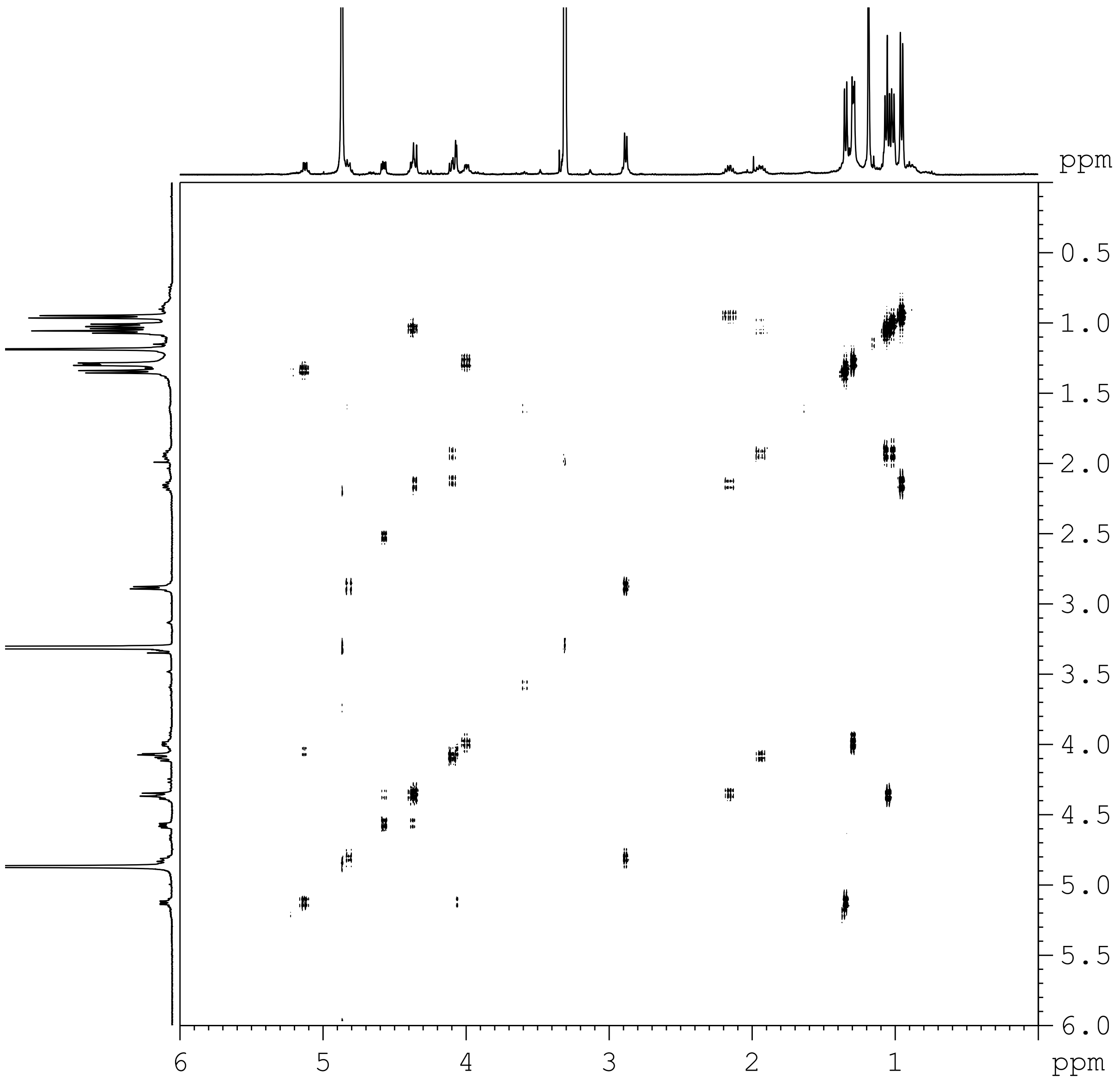

Cyclic depsipeptide (17) HSQC

```
Current Data Parameters
NAME          JC377hsqc
EXPNO                 3
PROCNO                1

F2 - Acquisition Parameters
Date_          20090627
Time               2.07
INSTRUM           spect
PROBHD   5 mm BBI 1H-BB
PULPROG      invietgpsi
TD                 4096
SOLVENT           CDCl3
NS                   64
DS                    8
SWH            4006.410 Hz
FIDRES         0.978127 Hz
AQ            0.5112308 sec
RG               1625.5
DW              124.800 usec
DE                 6.00 usec
TE                300.0 K
CNST2       140.0000000
d0           0.00000300 sec
D1           1.29999995 sec
d4           0.00178571 sec
d11          0.03000000 sec
d13          0.00000400 sec
D16          0.00015000 sec
D24          0.00178500 sec
DELTA        0.00172350 sec
DELTA1       0.00165800 sec
IN0          0.00003549 sec
MCREST   0 sec
MCWRK        0.21666709 sec
ST1CNT                0

======== CHANNEL f1 ========
NUC1                 1H
P1                 8.75 usec
p2                17.50 usec
P28                0.50 usec
PL1               -3.00 dB
SFO1        400.1318006 MHz

======== CHANNEL f2 ========
CPDPRG2            garp
NUC2                13C
P3                12.00 usec
p4                24.00 usec
PCPD2             70.00 usec
PL2               -6.00 dB
PL12              10.70 dB
SFO2        100.6197719 MHz

====== GRADIENT CHANNEL =====
GPNAM1         sine.100
GPNAM2         sine.100
GPX1     0 %
GPX2     0 %
GPY1     0 %
GPY2     0 %
GPZ1              80.00 %
GPZ2              20.10 %
P16             1500.00 usec

F1 - Acquisition parameters
TD                  512
SFO1           100.6198 MHz
FIDRES        27.518492 Hz
SW              140.027 ppm
FnMODE    Echo-Antiecho

F2 - Processing parameters
SI                 1024
SF          400.1300073 MHz
WDW                SINE
SSB                   1
LB       0 Hz
GB       0
PC                 1.40

F1 - Processing parameters
SI                  512
MC2       echo-antiecho
SF          100.6126510 MHz
WDW
SSB                   1
LB       0 Hz
GB       0
```

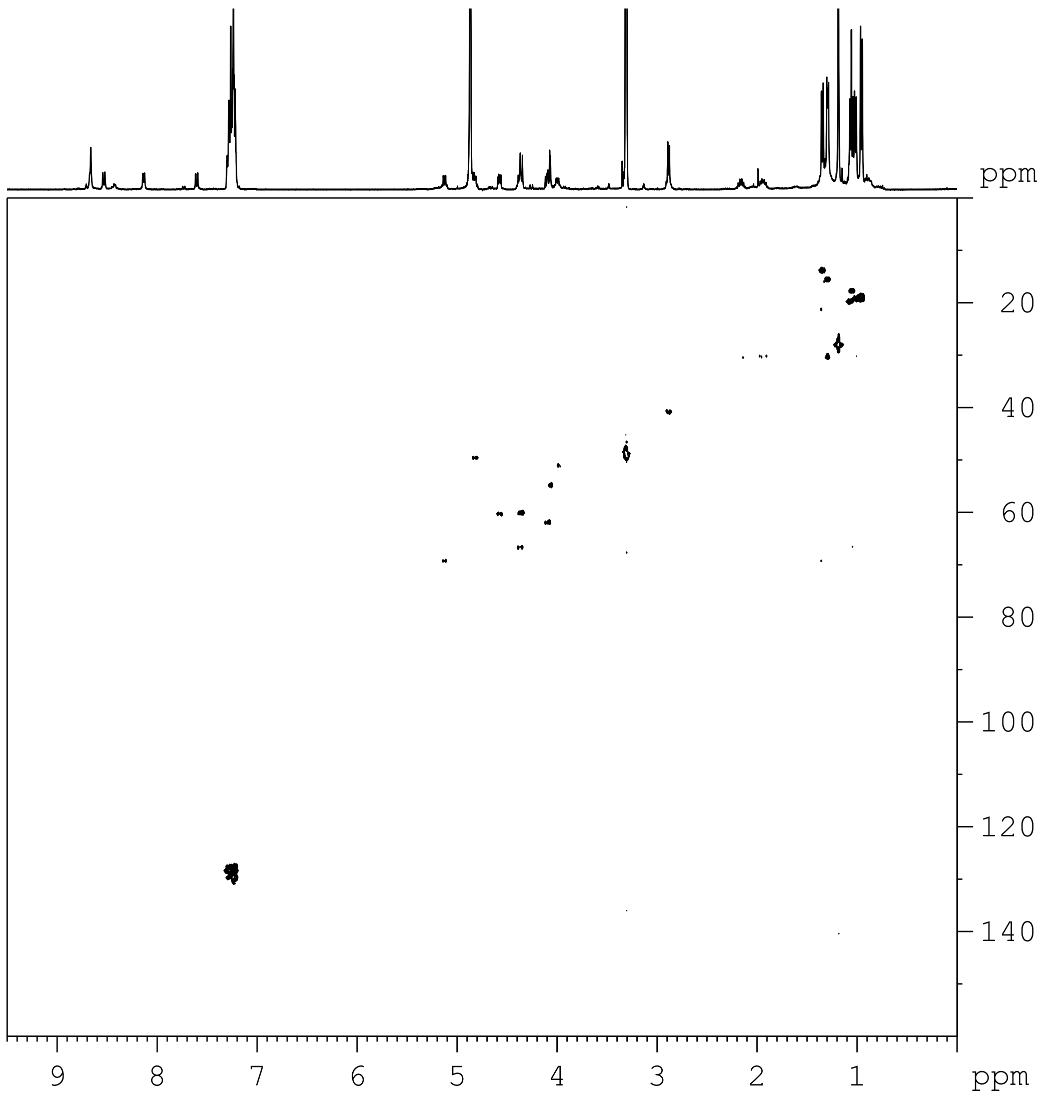

Cyclic depsipeptide (17) HMBC

```
Current Data Parameters
NAME          JC377hsqc
EXPNO                 4
PROCNO                1

F2 - Acquisition Parameters
Date_          20090627
Time              18.53
INSTRUM           spect
PROBHD   5 mm BBI 1H-BB
PULPROG  inv4gplplrndqf
TD                 4096
SOLVENT            MeOH
NS                   64
DS                   16
SWH            4006.410 Hz
FIDRES         0.978127 Hz
AQ            0.5112308 sec
RG                 2048
DW              124.800 usec
DE                 6.00 usec
TE                300.0 K
CNST2       140.0000000
d0           0.00000300 sec
D1           1.29999995 sec
d2           0.00357143 sec
D6           0.07200000 sec
d13          0.00000400 sec
D16          0.00015000 sec
IN0          0.00002485 sec
MCREST   0 sec
MCWRK        1.29999995 sec

======== CHANNEL f1 ========
NUC1                 1H
P1                 8.75 usec
p2                17.50 usec
PL1               -3.00 dB
SFO1        400.1318000 MHz

======== CHANNEL f2 ========
NUC2                13C
P3                12.00 usec
PL2               -6.00 dB
SFO2        100.6227903 MHz

====== GRADIENT CHANNEL =====
GPNAM1         sine.100
GPNAM2         sine.100
GPNAM3         sine.100
GPX1     0 %
GPX2     0 %
GPX3     0 %
GPY1     0 %
GPY2     0 %
GPY3     0 %
GPZ1              50.00 %
GPZ2              30.00 %
GPZ3              40.10 %
P16             1500.00 usec

F1 - Acquisition parameters
TD                  512
SFO1           100.6228 MHz
FIDRES        39.298290 Hz
SW              199.962 ppm
FnMODE               QF

F2 - Processing parameters
SI                 4096
SF          400.1300078 MHz
WDW                SINE
SSB                   2
LB       0 Hz
GB       0
PC                 1.40

F1 - Processing parameters
SI                 1024
MC2                  QF
SF          100.6126295 MHz
WDW              States
SSB                   2
LB       0 Hz
GB       0
```

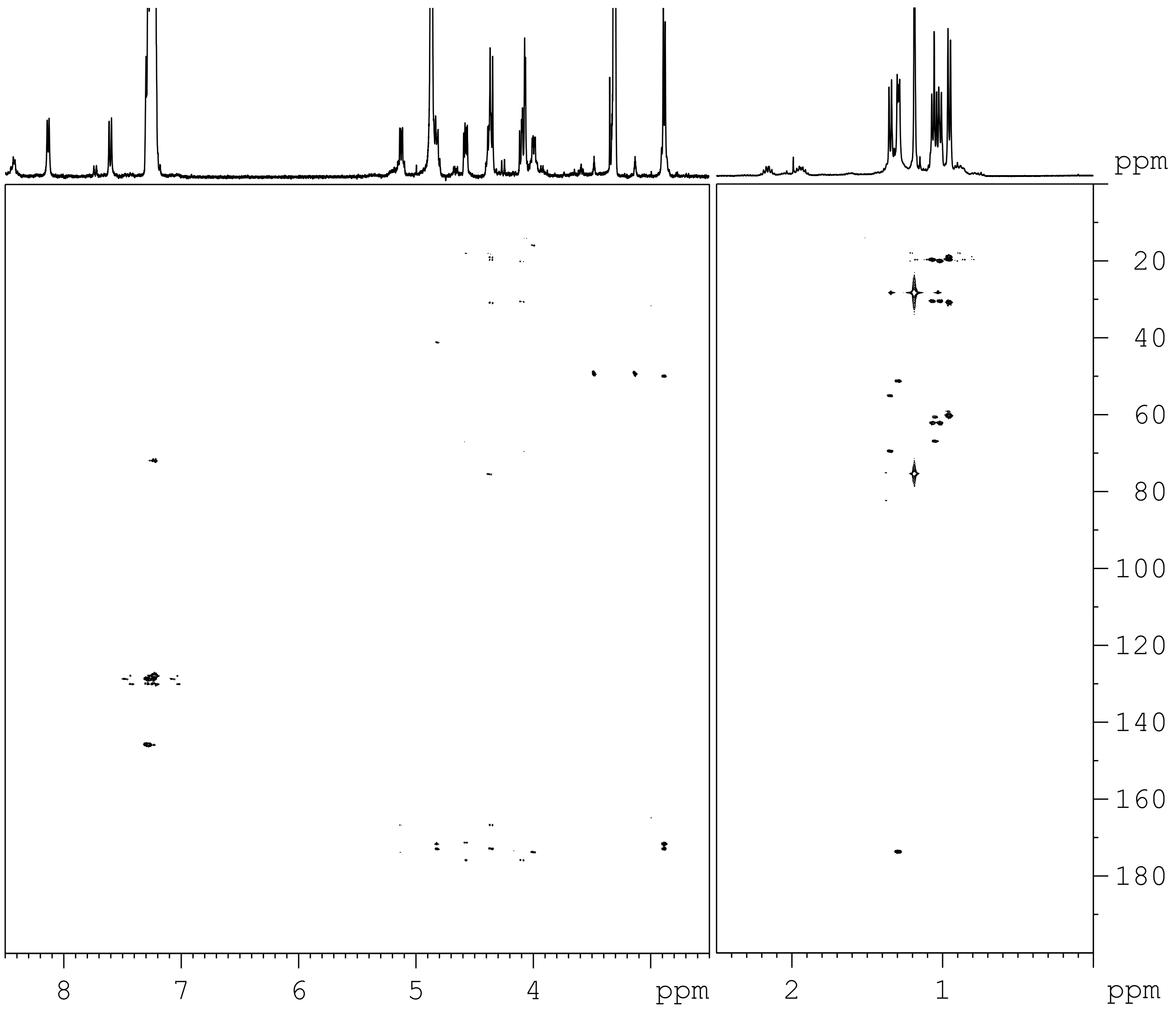

Cyclic depsipeptide (22)

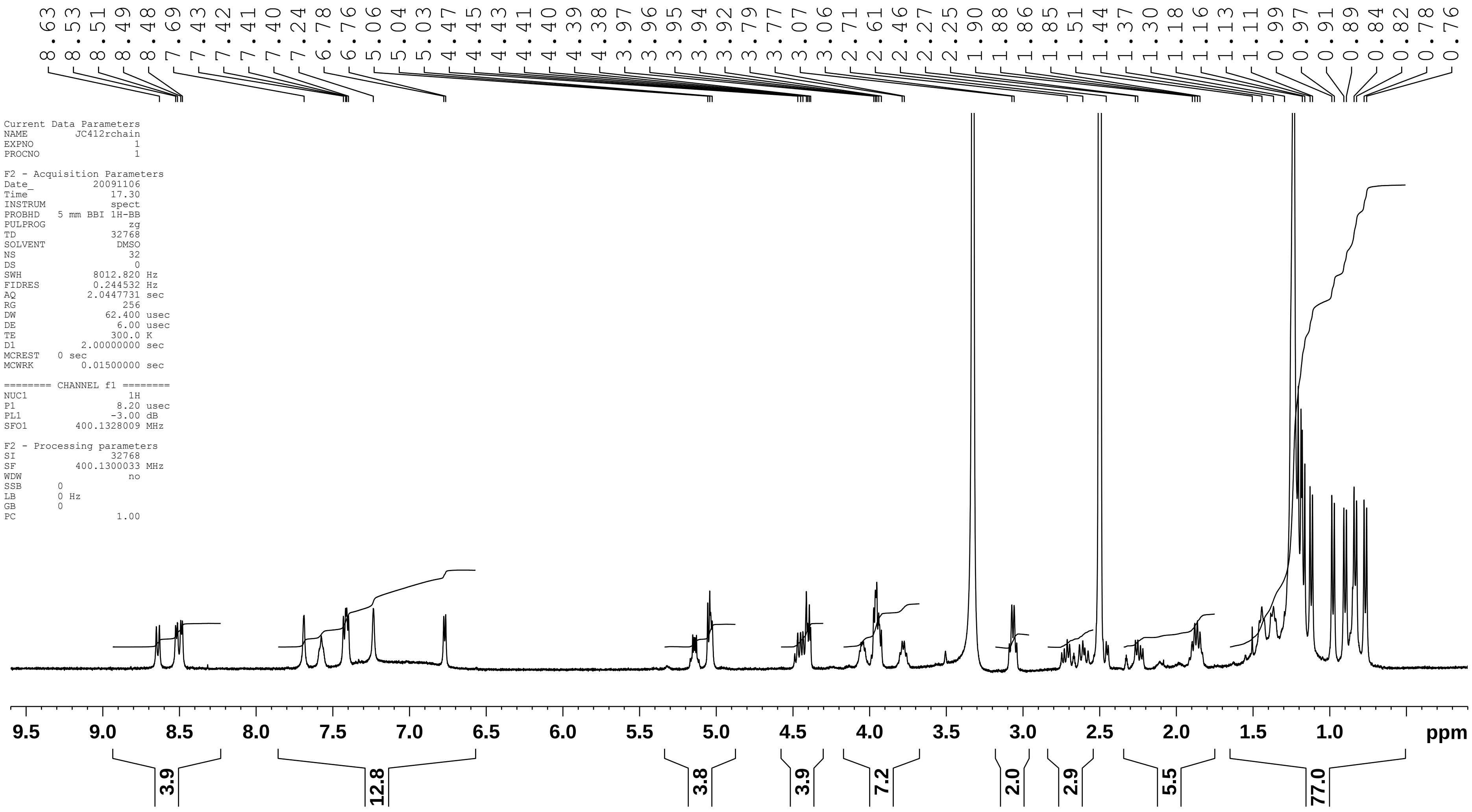

Cyclic depsipeptide (22) COSY

```
Current Data Parameters
NAME        JC398DMSO1b
EXPNO                 2
PROCNO                1

F2 - Acquisition Parameters
Date_          20090918
Time              18.36
INSTRUM           spect
PROBHD   5 mm BBI 1H-BB
PULPROG        cosygpqf
TD                 4096
SOLVENT           CDCl3
NS                  128
DS                   16
SWH            4006.410 Hz
FIDRES         0.978127 Hz
AQ            0.5112308 sec
RG                 1024
DW              124.800 usec
DE                 6.00 usec
TE                300.0 K
d0           0.00000300 sec
D1           2.00000000 sec
d13          0.00000400 sec
D16          0.00015000 sec
IN0          0.00024988 sec
MCREST   0 sec
MCWRK        2.00000000 sec

======== CHANNEL f1 ========
NUC1                 1H
P0                 5.30 usec
P1                 5.30 usec
PL1               -3.00 dB
SFO1        400.2099789 MHz

====== GRADIENT CHANNEL =====
GPNAM1         SINE.100
GPNAM2         SINE.100
GPX1     0 %
GPX2     0 %
GPY1     0 %
GPY2     0 %
GPZ1              10.00 %
GPZ2              10.00 %
P16             1500.00 usec

F1 - Acquisition parameters
TD                  128
SFO1             400.21 MHz
FIDRES        31.265633 Hz
SW               10.000 ppm
FnMODE               QF

F2 - Processing parameters
SI                 2048
SF          400.2081745 MHz
WDW                SINE
SSB      0
LB       0 Hz
GB       0
PC                 1.00

F1 - Processing parameters
SI                 1024
MC2                  QF
SF          400.2081743 MHz
WDW              States
SSB      0
LB       0 Hz
GB       0
```

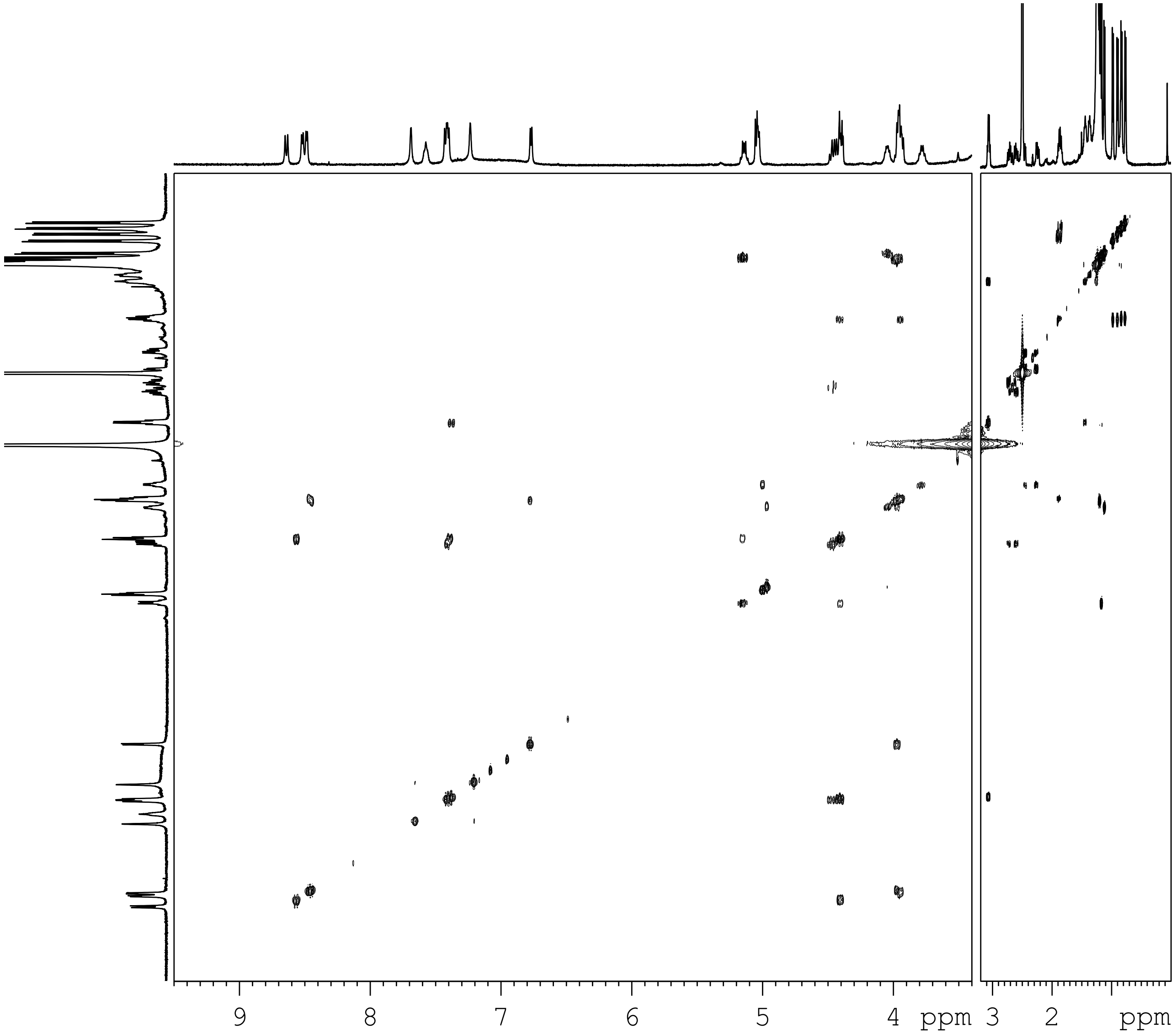

Cyclic depsipeptide (22)

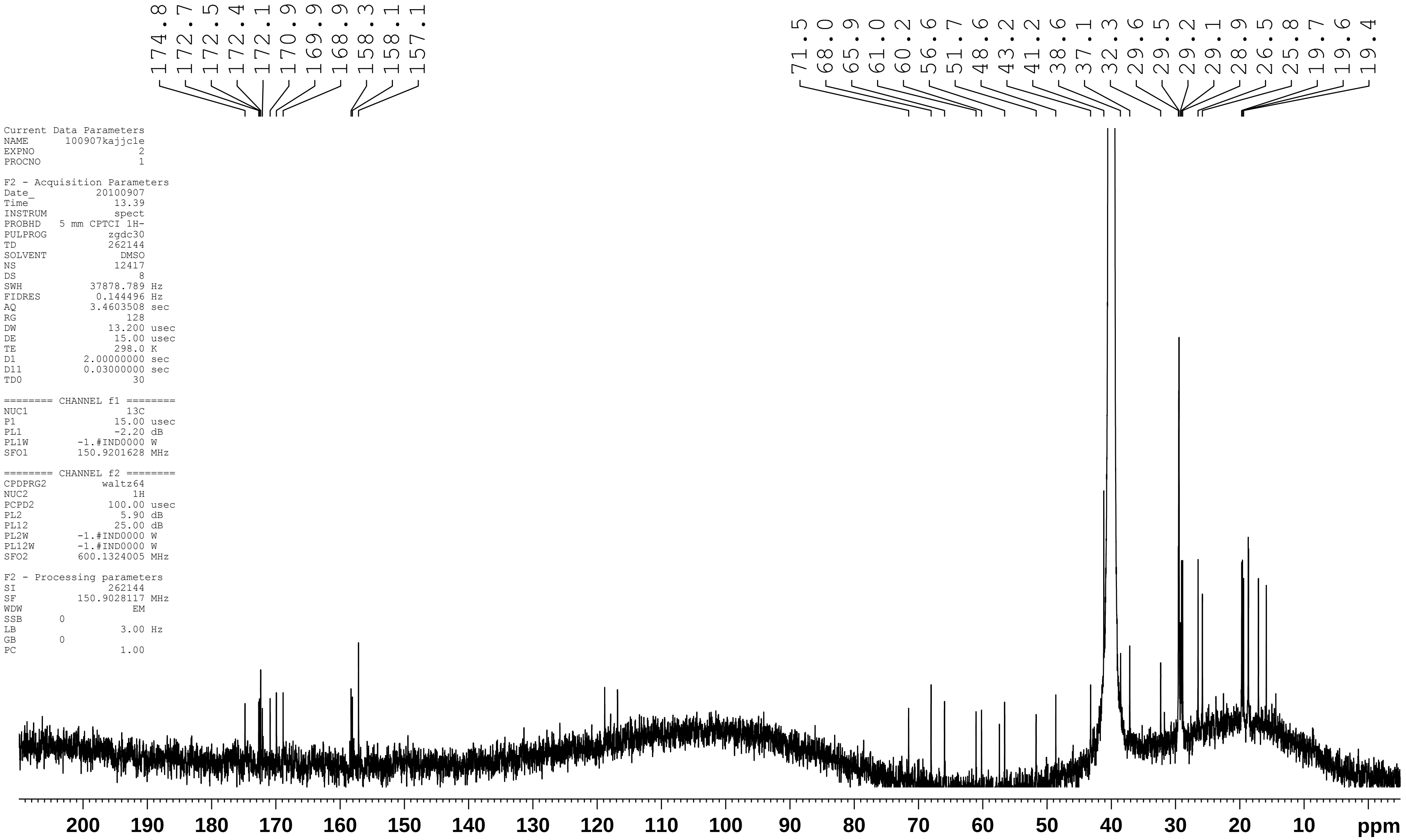

Cyclic depsipeptide (22) HSQC

```
Current Data Parameters
NAME     JC398403DMSOHMBC
EXPNO                   2
PROCNO                  1

F2 - Acquisition Parameters
Date_            20090929
Time                18.08
INSTRUM             spect
PROBHD   5 mm BBI 1H-BB
PULPROG         invietgpsi
TD                   2048
SOLVENT              DMSO
NS                    256
DS                      8
SWH              4006.410 Hz
FIDRES           1.956255 Hz
AQ              0.2556404 sec
RG                   4096
DW                124.800 usec
DE                   6.00 usec
TE                  300.0 K
CNST2         145.0000000
d0             0.00000300 sec
D1             2.00000000 sec
d4             0.00172414 sec
d11            0.03000000 sec
d13            0.00000400 sec
D16            0.00015000 sec
D24            0.00172000 sec
DELTA          0.00171660 sec
DELTA1         0.00165800 sec
IN0            0.00004140 sec
MCREST   0 sec
MCWRK          0.33333400 sec
ST1CNT                  0

======== CHANNEL f1 ========
NUC1                   1H
P1                   5.30 usec
p2                  10.60 usec
P28               2000.00 usec
PL1                 -3.00 dB
SFO1          400.2099789 MHz

======== CHANNEL f2 ========
CPDPRG2              garp
NUC2                  13C
P3                  12.00 usec
p4                  24.00 usec
PCPD2               80.00 usec
PL2                 -6.00 dB
PL12                10.48 dB
SFO2          100.6374576 MHz

====== GRADIENT CHANNEL =====
GPNAM1           sine.100
GPNAM2           sine.100
GPX1     0 %
GPX2     0 %
GPY1     0 %
GPY2     0 %
GPZ1                80.00 %
GPZ2                20.10 %
P16               1500.00 usec

F1 - Acquisition parameters
TD                    110
SFO1             100.6375 MHz
FIDRES         109.793587 Hz
SW                120.008 ppm
FnMODE     Echo-Antiecho

F2 - Processing parameters
SI                   2048
SF            400.2081751 MHz
WDW                  SINE
SSB                     2
LB       0 Hz
GB       0
PC                   1.00

F1 - Processing parameters
SI                    512
MC2         echo-antiecho
SF            100.6325017 MHz
WDW
SSB                     2
LB       0 Hz
GB       0
```

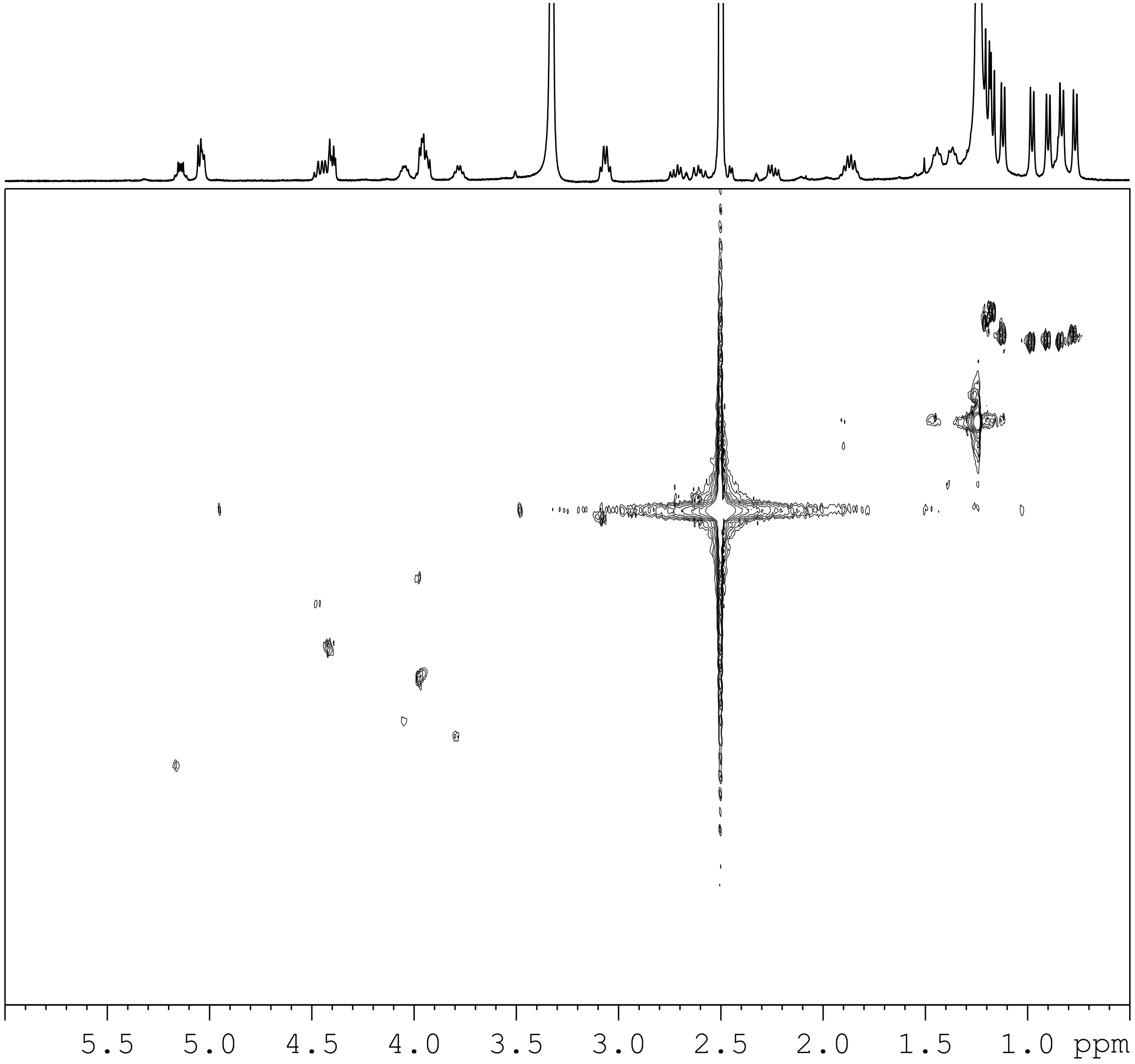

Cyclic depsipeptide (22) HMBC

```
Current Data Parameters
NAME     JC398403DMSOHMBC
EXPNO                   2
PROCNO                  1

F2 - Acquisition Parameters
Date_          20090929
Time              18.08
INSTRUM           spect
PROBHD   5 mm BBI 1H-BB
PULPROG      invietgpsi
TD                 2048
SOLVENT            DMSO
NS                  256
DS                    8
SWH            4006.410 Hz
FIDRES         1.956255 Hz
AQ            0.2556404 sec
RG                 4096
DW              124.800 usec
DE                 6.00 usec
TE                300.0 K
CNST2       145.0000000
d0           0.00000300 sec
D1           2.00000000 sec
d4           0.00172414 sec
d11          0.03000000 sec
d13          0.00000400 sec
D16          0.00015000 sec
D24          0.00172000 sec
DELTA        0.00171660 sec
DELTA1       0.00165800 sec
IN0          0.00004140 sec
MCREST   0 sec
MCWRK        0.33333400 sec
ST1CNT                0

======== CHANNEL f1 ========
NUC1                 1H
P1                 5.30 usec
p2                10.60 usec
P28             2000.00 usec
PL1               -3.00 dB
SFO1        400.2099789 MHz

======== CHANNEL f2 ========
CPDPRG2            garp
NUC2                13C
P3                12.00 usec
p4                24.00 usec
PCPD2             80.00 usec
PL2               -6.00 dB
PL12              10.48 dB
SFO2        100.6374576 MHz

====== GRADIENT CHANNEL =====
GPNAM1         sine.100
GPNAM2         sine.100
GPX1     0 %
GPX2     0 %
GPY1     0 %
GPY2     0 %
GPZ1              80.00 %
GPZ2              20.10 %
P16             1500.00 usec

F1 - Acquisition parameters
TD                  110
SFO1           100.6375 MHz
FIDRES       109.793587 Hz
SW              120.008 ppm
FnMODE    Echo-Antiecho

F2 - Processing parameters
SI                 2048
SF          400.2081751 MHz
WDW                SINE
SSB                   2
LB       0 Hz
GB       0
PC                 1.00

F1 - Processing parameters
SI                  512
MC2       echo-antiecho
SF          100.6325017 MHz
WDW
SSB                   2
LB       0 Hz
GB       0
```

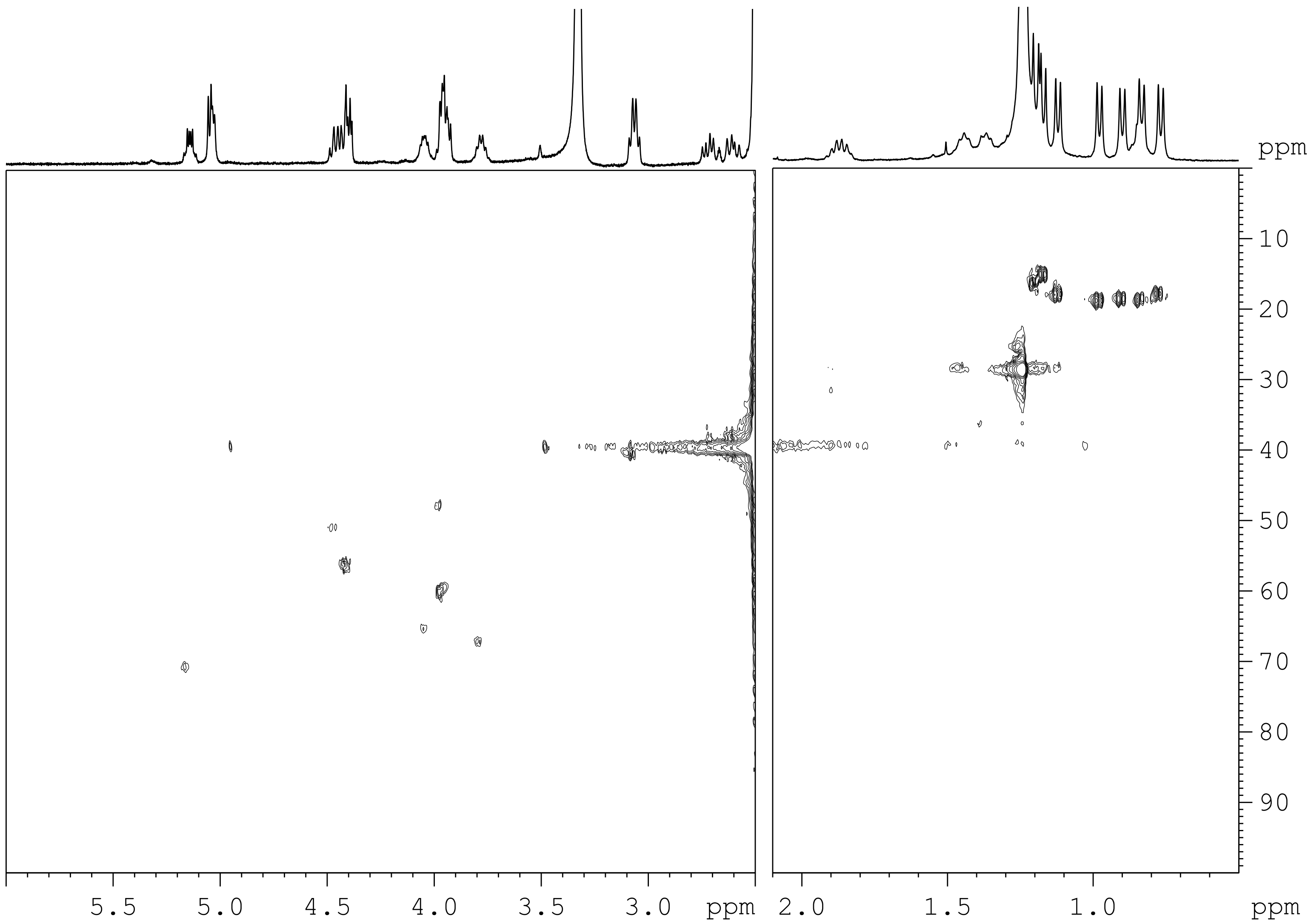

Cyclic depsipeptide (22) HMBC

```
Current Data Parameters
NAME     JC398403DMSOHMBC
EXPNO                   4
PROCNO                  1

F2 - Acquisition Parameters
Date_            20091002
Time                16.53
INSTRUM             spect
PROBHD   5 mm BBI 1H-BB
PULPROG  inv4gplplrndqf
TD                   4096
SOLVENT              DMSO
NS                    512
DS                      8
SWH              4006.410 Hz
FIDRES           0.978127 Hz
AQ              0.5112308 sec
RG                11585.3
DW                124.800 usec
DE                   6.00 usec
TE                  300.0 K
CNST2         145.0000000
d0             0.00000300 sec
D1             2.00000000 sec
d2             0.00344828 sec
D6             0.06250000 sec
d13            0.00000400 sec
D16            0.00015000 sec
IN0            0.00002259 sec
MCREST   0 sec
MCWRK          2.00000000 sec

======== CHANNEL f1 ========
NUC1                   1H
P1                   5.30 usec
p2                  10.60 usec
PL1                 -3.00 dB
SFO1          400.2099789 MHz

======== CHANNEL f2 ========
NUC2                  13C
P3                  12.00 usec
PL2                 -6.00 dB
SFO2          100.6424892 MHz

====== GRADIENT CHANNEL =====
GPNAM1           sine.100
GPNAM2           sine.100
GPNAM3           sine.100
GPX1     0 %
GPX2     0 %
GPX3     0 %
GPY1     0 %
GPY2     0 %
GPY3     0 %
GPZ1                50.00 %
GPZ2                30.00 %
GPZ3                40.10 %
P16               1500.00 usec

F1 - Acquisition parameters
TD                    240
SFO1             100.6425 MHz
FIDRES          92.233902 Hz
SW                219.948 ppm
FnMODE                 QF

F2 - Processing parameters
SI                   2048
SF            400.2081767 MHz
WDW                  SINE
SSB                     2
LB       0 Hz
GB       0
PC                   1.00

F1 - Processing parameters
SI                   1024
MC2                    QF
SF            100.6324924 MHz
WDW                States
SSB                     2
LB       0 Hz
GB       0
```

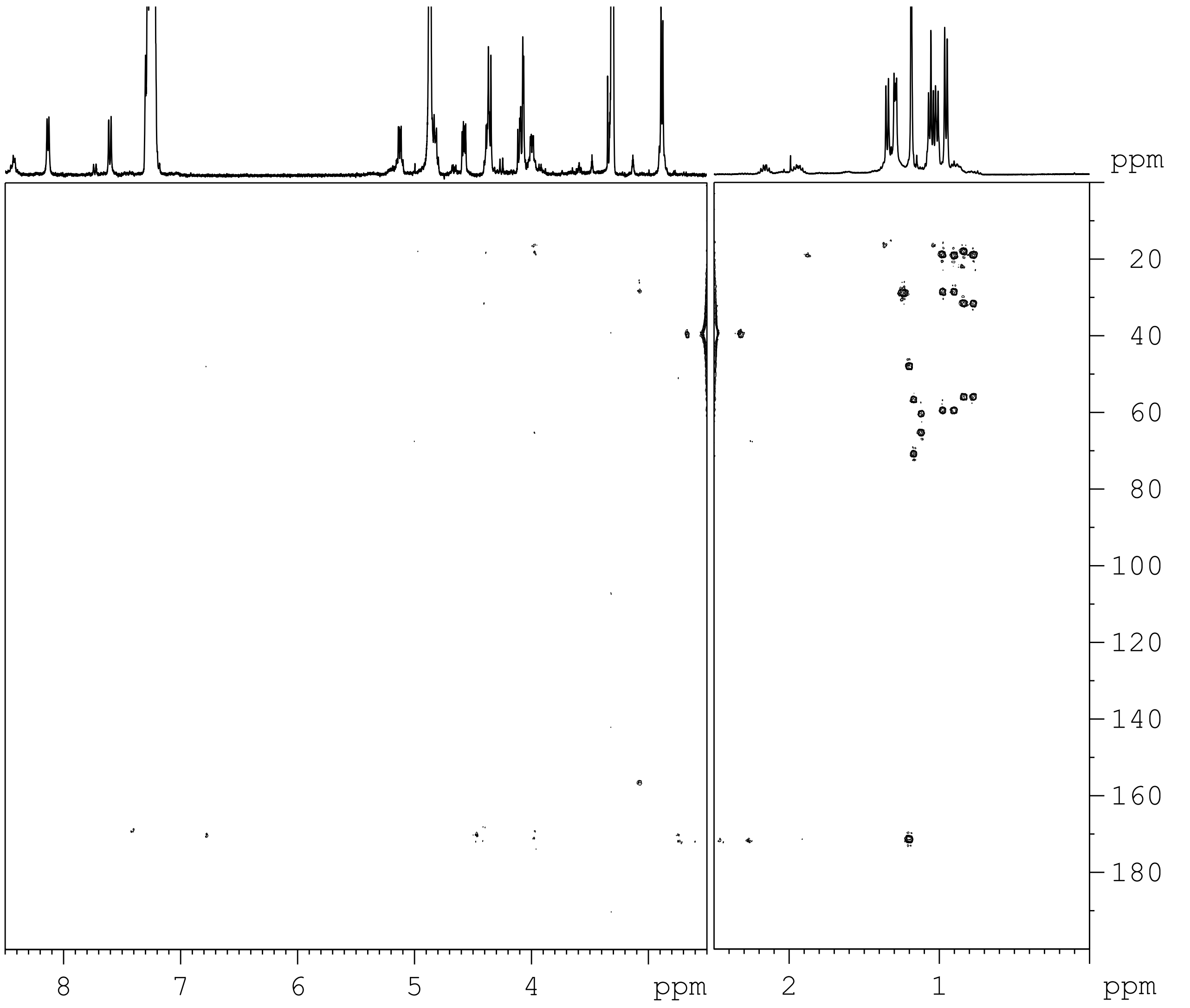

Cyclic depsipeptide (22) HMBC

```
Current Data Parameters
NAME     JC398403DMSOHMBC
EXPNO                   4
PROCNO                  1

F2 - Acquisition Parameters
Date_          20091002
Time              16.53
INSTRUM           spect
PROBHD   5 mm BBI 1H-BB
PULPROG  inv4gplplrndqf
TD                 4096
SOLVENT            DMSO
NS                  512
DS                    8
SWH            4006.410 Hz
FIDRES         0.978127 Hz
AQ            0.5112308 sec
RG              11585.3
DW              124.800 usec
DE                 6.00 usec
TE                300.0 K
CNST2       145.0000000
d0           0.00000300 sec
D1           2.00000000 sec
d2           0.00344828 sec
D6           0.06250000 sec
d13          0.00000400 sec
D16          0.00015000 sec
IN0          0.00002259 sec
MCREST   0 sec
MCWRK        2.00000000 sec

======== CHANNEL f1 ========
NUC1                 1H
P1                 5.30 usec
p2                10.60 usec
PL1               -3.00 dB
SFO1        400.2099789 MHz

======== CHANNEL f2 ========
NUC2                13C
P3                12.00 usec
PL2               -6.00 dB
SFO2        100.6424892 MHz

====== GRADIENT CHANNEL =====
GPNAM1         sine.100
GPNAM2         sine.100
GPNAM3         sine.100
GPX1      0 %
GPX2      0 %
GPX3      0 %
GPY1      0 %
GPY2      0 %
GPY3      0 %
GPZ1              50.00 %
GPZ2              30.00 %
GPZ3              40.10 %
P16             1500.00 usec

F1 - Acquisition parameters
TD                  240
SFO1           100.6425 MHz
FIDRES        92.233902 Hz
SW              219.948 ppm
FnMODE               QF

F2 - Processing parameters
SI                 2048
SF          400.2081767 MHz
WDW                SINE
SSB                   2
LB        0 Hz
GB        0
PC                 1.00

F1 - Processing parameters
SI                 1024
MC2                  QF
SF          100.6324924 MHz
WDW              States
SSB                   2
LB        0 Hz
GB        0
```

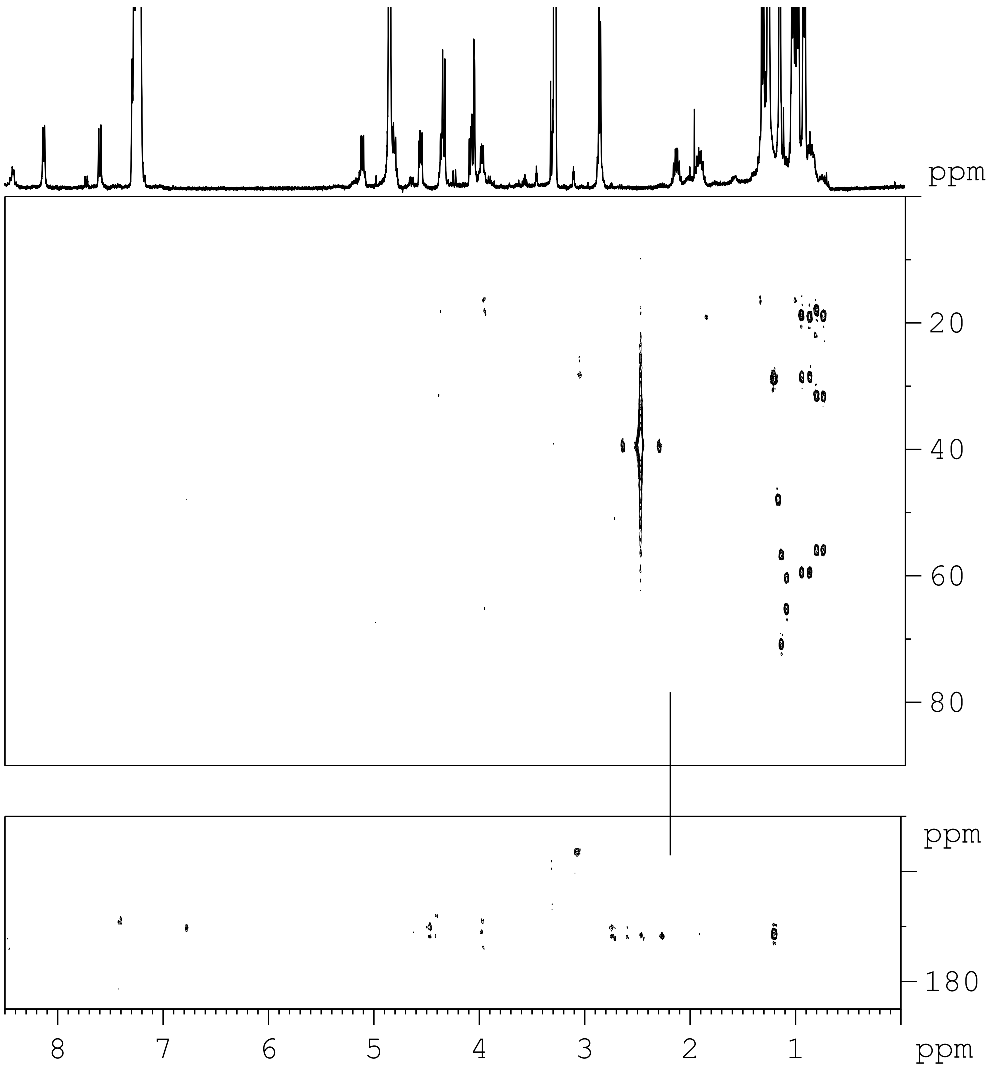

Cyclic depsipeptide (23)

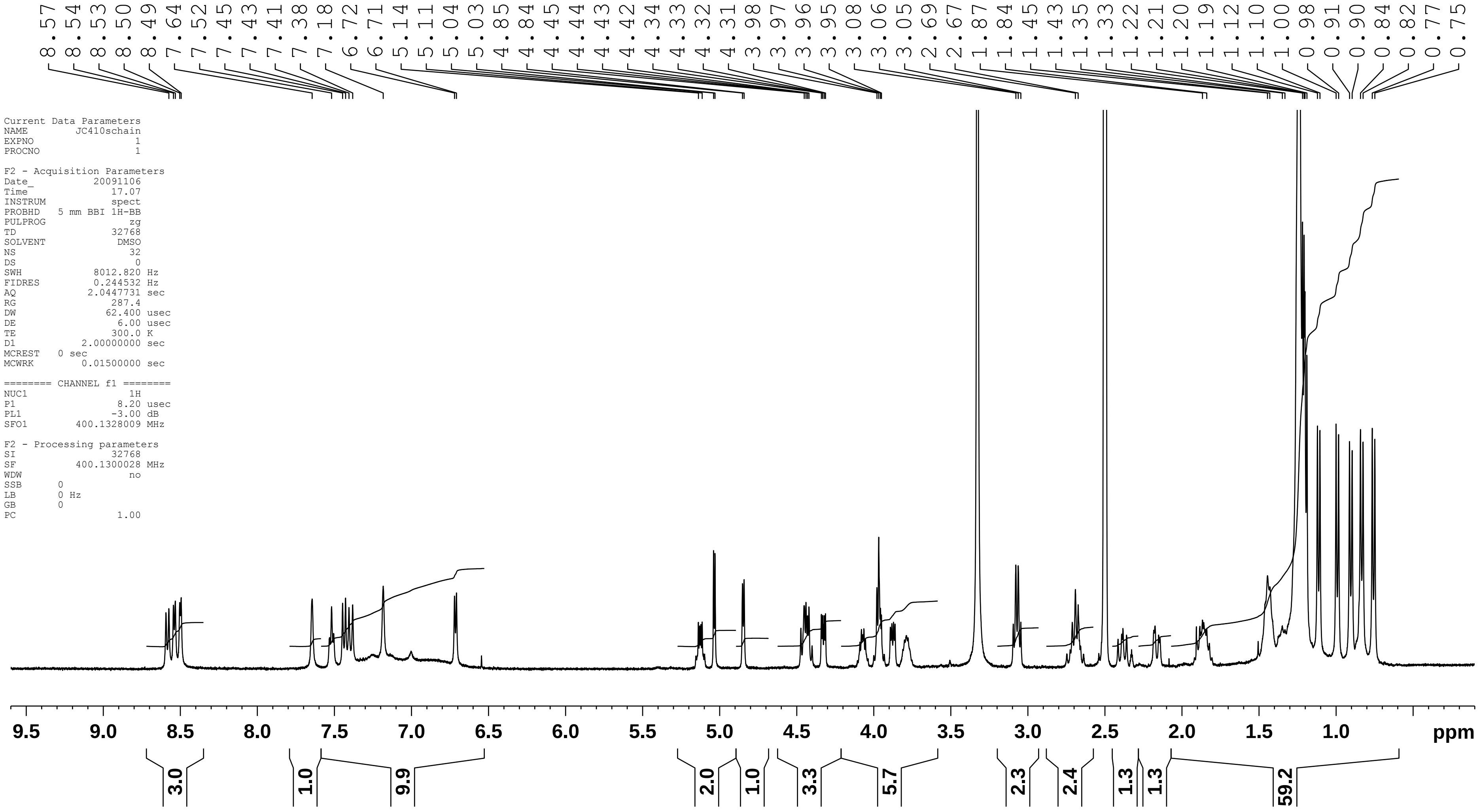

Cyclic depsipeptide (23) COSY

```
Current Data Parameters
NAME              EpimerS
EXPNO                   2
PROCNO                  1

F2 - Acquisition Parameters
Date_            20090619
Time                21.13
INSTRUM             spect
PROBHD   5 mm BBI 1H-BB
PULPROG       cosygpmfph
TD                   4096
SOLVENT              MeOH
NS                     64
DS                      8
SWH              4006.410 Hz
FIDRES           0.978127 Hz
AQ              0.5112308 sec
RG                 1625.5
DW                124.800 usec
DE                   6.00 usec
TE                  323.1 K
d0             0.00011452 sec
D1             2.00000000 sec
d13            0.00000400 sec
D16            0.00015000 sec
d20            0.00165400 sec
IN0            0.00024991 sec
MCREST   0 sec
MCWRK          1.00000000 sec
ST1CNT                  0

======== CHANNEL f1 ========
NUC1                   1H
P1                   8.20 usec
p2                  16.40 usec
PL1                 -3.00 dB
SFO1          400.1318006 MHz

====== GRADIENT CHANNEL =====
GPNAM1           sine.100
GPNAM2           sine.100
GPX1     0 %
GPX2     0 %
GPY1     0 %
GPY2     0 %
GPZ1                10.00 %
GPZ2                20.00 %
P16               1500.00 usec

F1 - Acquisition parameters
TD                    256
SFO1             400.1318 MHz
FIDRES          15.630470 Hz
SW                 10.000 ppm
FnMODE        States-TPPI

F2 - Processing parameters
SI                   4096
SF            400.1300130 MHz
WDW                  SINE
SSB                     2
LB       0 Hz
GB       0
PC                   1.00

F1 - Processing parameters
SI                   1024
MC2           States-TPPI
SF            400.1300096 MHz
WDW
SSB                     2
LB       0 Hz
GB       0
```

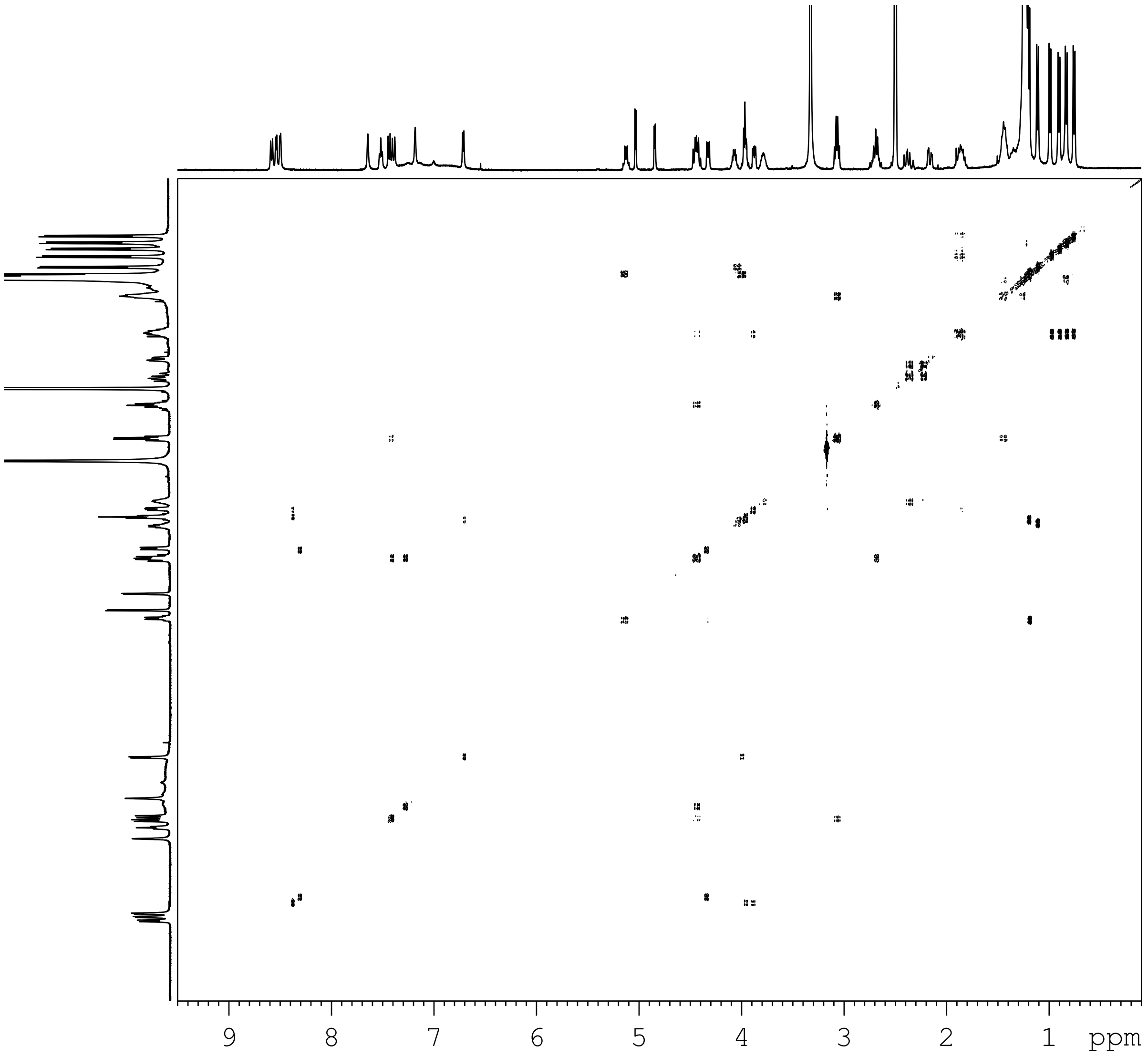

Cyclic depsipeptide (23)

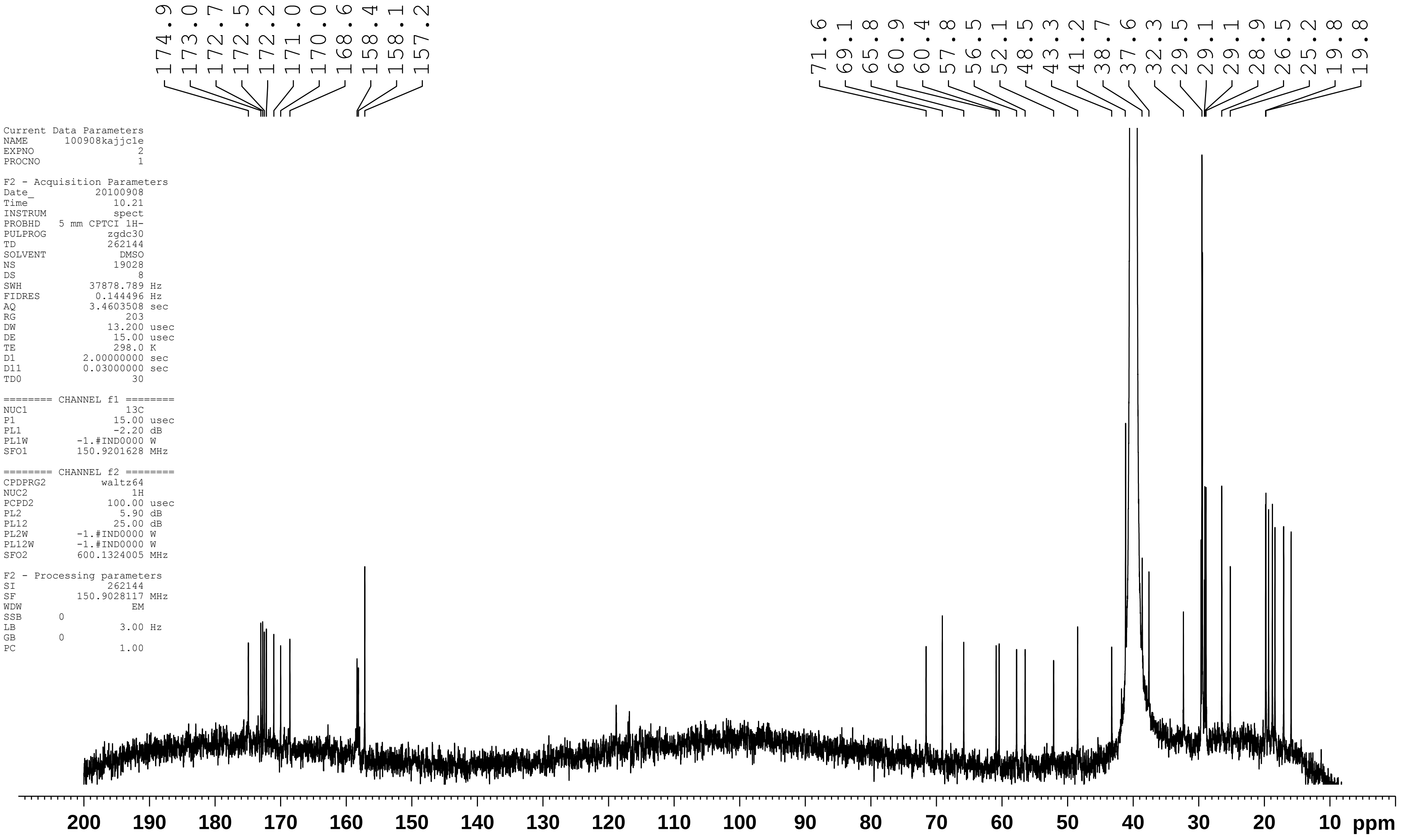


```
Current Data Parameters
NAME      100908kajjc1e
EXPNO                 2
PROCNO                1

F2 - Acquisition Parameters
Date_          20100908
Time              10.21
INSTRUM           spect
PROBHD   5 mm CPTCI 1H-
PULPROG         zgdc30
TD               262144
SOLVENT            DMSO
NS                19028
DS                    8
SWH           37878.789 Hz
FIDRES         0.144496 Hz
AQ            3.4603508 sec
RG                  203
DW               13.200 usec
DE                15.00 usec
TE                298.0 K
D1           2.00000000 sec
D11          0.03000000 sec
TD0                  30

======== CHANNEL f1 ========
NUC1                13C
P1                15.00 usec
PL1               -2.20 dB
PL1W        -1.#IND0000 W
SFO1        150.9201628 MHz

======== CHANNEL f2 ========
CPDPRG2          waltz64
NUC2                 1H
PCPD2            100.00 usec
PL2                5.90 dB
PL12              25.00 dB
PL2W        -1.#IND0000 W
PL12W       -1.#IND0000 W
SFO2        600.1324005 MHz

F2 - Processing parameters
SI               262144
SF          150.9028117 MHz
WDW                  EM
SSB      0
LB                 3.00 Hz
GB       0
PC                 1.00
```

Cyclic depsipeptide (23) HSQC

```
Current Data Parameters
NAME             EpimerS
EXPNO                  3
PROCNO                 1

F2 - Acquisition Parameters
Date_           20091009
Time               19.20
INSTRUM            spect
PROBHD   5 mm BBI 1H-BB
PULPROG       invietgpsi
TD                  4096
SOLVENT             DMSO
NS                  1024
DS                     8
SWH             4006.410 Hz
FIDRES          0.978127 Hz
AQ             0.5112308 sec
RG                  1024
DW               124.800 usec
DE                  6.00 usec
TE                 300.0 K
CNST2        140.0000000
d0            0.00000300 sec
D1            2.00000000 sec
d4            0.00178571 sec
d11           0.03000000 sec
d13           0.00000400 sec
D16           0.00015000 sec
D24           0.00178500 sec
DELTA         0.00172240 sec
DELTA1        0.00165800 sec
IN0           0.00004969 sec
MCREST   0 sec
MCWRK         0.33333400 sec
ST1CNT                 0

======== CHANNEL f1 ========
NUC1                  1H
P1                  8.20 usec
p2                 16.40 usec
P28                 0.50 usec
PL1                -3.00 dB
SFO1         400.1318006 MHz

======== CHANNEL f2 ========
CPDPRG2             garp
NUC2                 13C
P3                 12.00 usec
p4                 24.00 usec
PCPD2              70.00 usec
PL2                -6.00 dB
PL12               10.70 dB
SFO2         100.6172566 MHz

====== GRADIENT CHANNEL =====
GPNAM1          sine.100
GPNAM2          sine.100
GPX1     0 %
GPX2     0 %
GPY1     0 %
GPY2     0 %
GPZ1               80.00 %
GPZ2               20.10 %
P16              1500.00 usec

F1 - Acquisition parameters
TD                    63
SFO1            100.6173 MHz
FIDRES        159.728455 Hz
SW               100.012 ppm
FnMODE    Echo-Antiecho

F2 - Processing parameters
SI                  2048
SF           400.1299998 MHz
WDW                 SINE
SSB                    2
LB       0 Hz
GB       0
PC                  1.00

F1 - Processing parameters
SI                   512
MC2       echo-antiecho
SF           100.6128422 MHz
WDW
SSB                    2
LB       0 Hz
GB       0
```

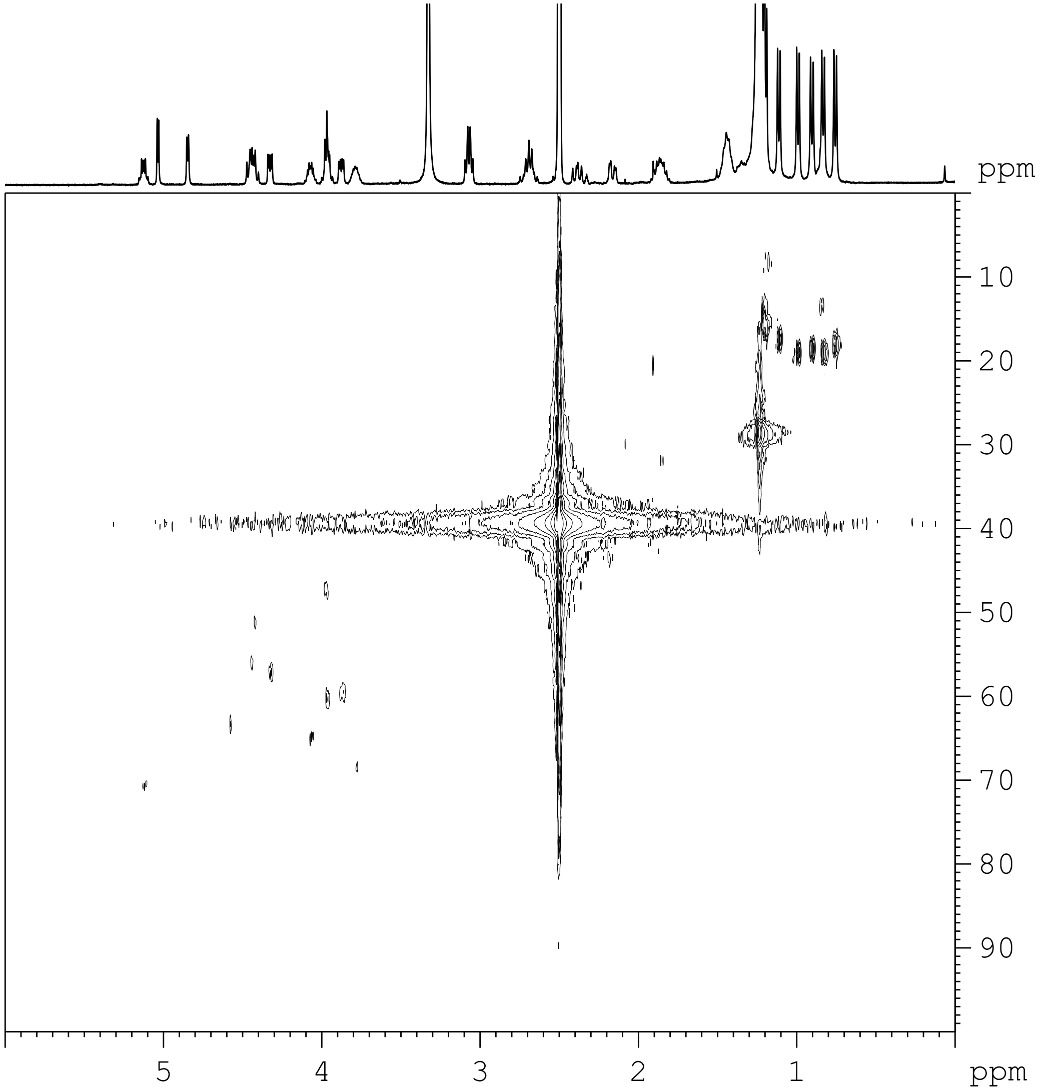

Cyclic depsipeptide (23) HMBC

```
Current Data Parameters
NAME          JC410schain
EXPNO                   4
PROCNO                  1

F2 - Acquisition Parameters
Date_            20091120
Time                20.23
INSTRUM             spect
PROBHD   5 mm BBI 1H-BB
PULPROG  inv4gplplrndqf
TD                   4096
SOLVENT              DMSO
NS                   1024
DS                      8
SWH              4006.410 Hz
FIDRES           0.978127 Hz
AQ              0.5112308 sec
RG                   2048
DW                124.800 usec
DE                   6.00 usec
TE                  300.0 K
CNST2         140.0000000
d0             0.00000300 sec
D1             1.20000005 sec
d2             0.00357143 sec
D6             0.10000000 sec
d13            0.00000400 sec
D16            0.00015000 sec
IN0            0.00002485 sec
MCREST   0 sec
MCWRK          1.20000005 sec

======== CHANNEL f1 ========
NUC1                   1H
P1                   8.38 usec
p2                  16.76 usec
PL1                 -3.00 dB
SFO1          400.1320007 MHz

======== CHANNEL f2 ========
NUC2                  13C
P3                  12.60 usec
PL2                 -6.00 dB
SFO2          100.6227903 MHz

====== GRADIENT CHANNEL =====
GPNAM1           sine.100
GPNAM2           sine.100
GPNAM3           sine.100
GPX1     0 %
GPX2     0 %
GPX3     0 %
GPY1     0 %
GPY2     0 %
GPY3     0 %
GPZ1                50.00 %
GPZ2                30.00 %
GPZ3                40.10 %
P16               1500.00 usec

F1 - Acquisition parameters
TD                     77
SFO1             100.6228 MHz
FIDRES         261.308105 Hz
SW                199.962 ppm
FnMODE                 QF

F2 - Processing parameters
SI                   2048
SF            400.1300000 MHz
WDW                  SINE
SSB      0
LB       0 Hz
GB       0
PC                   1.00

F1 - Processing parameters
SI                   2048
MC2                    QF
SF            100.6135390 MHz
WDW           States-TPPI
SSB      0
LB       0 Hz
GB       0
```

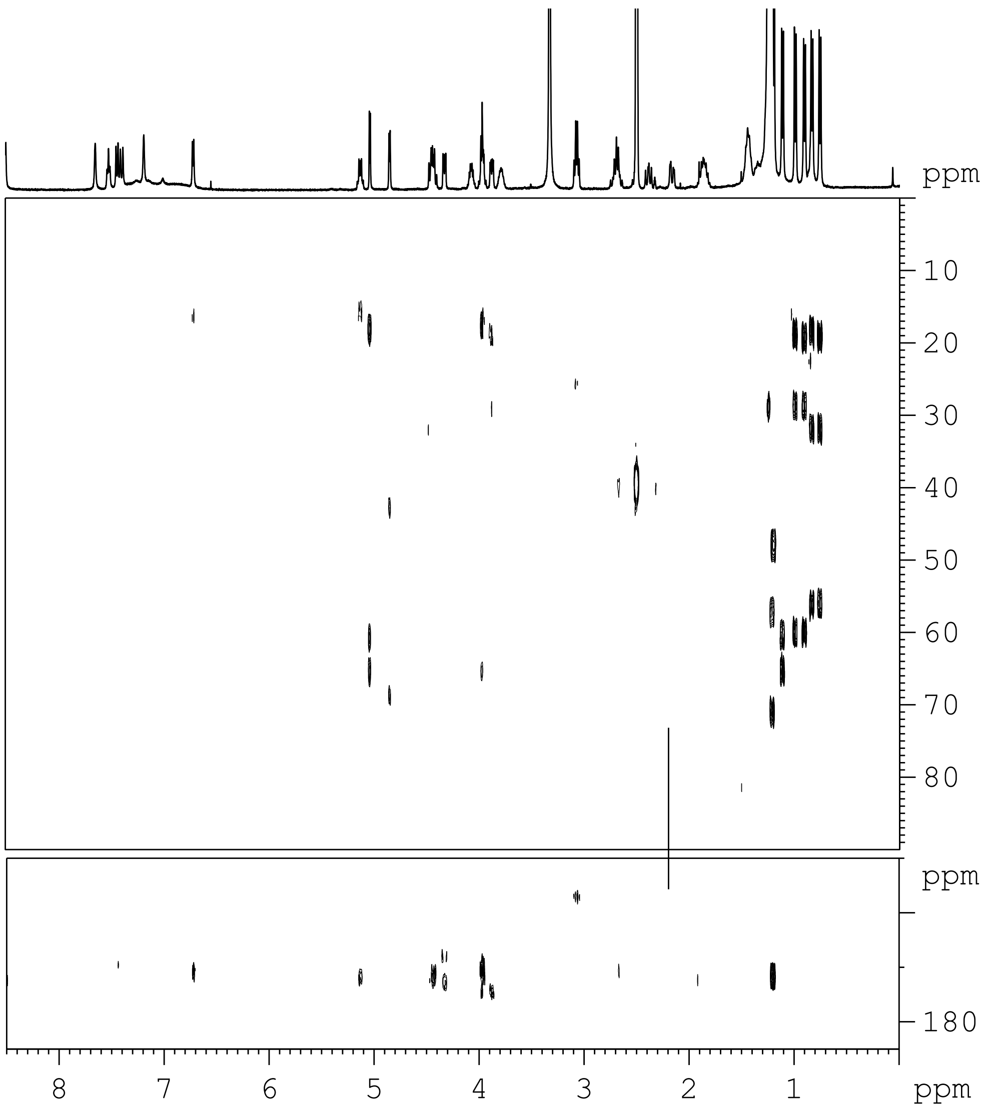

1-Methyl-15-[bis(*tert*-Butoxycarbonyl)guanidino] pentadecanoate (15)

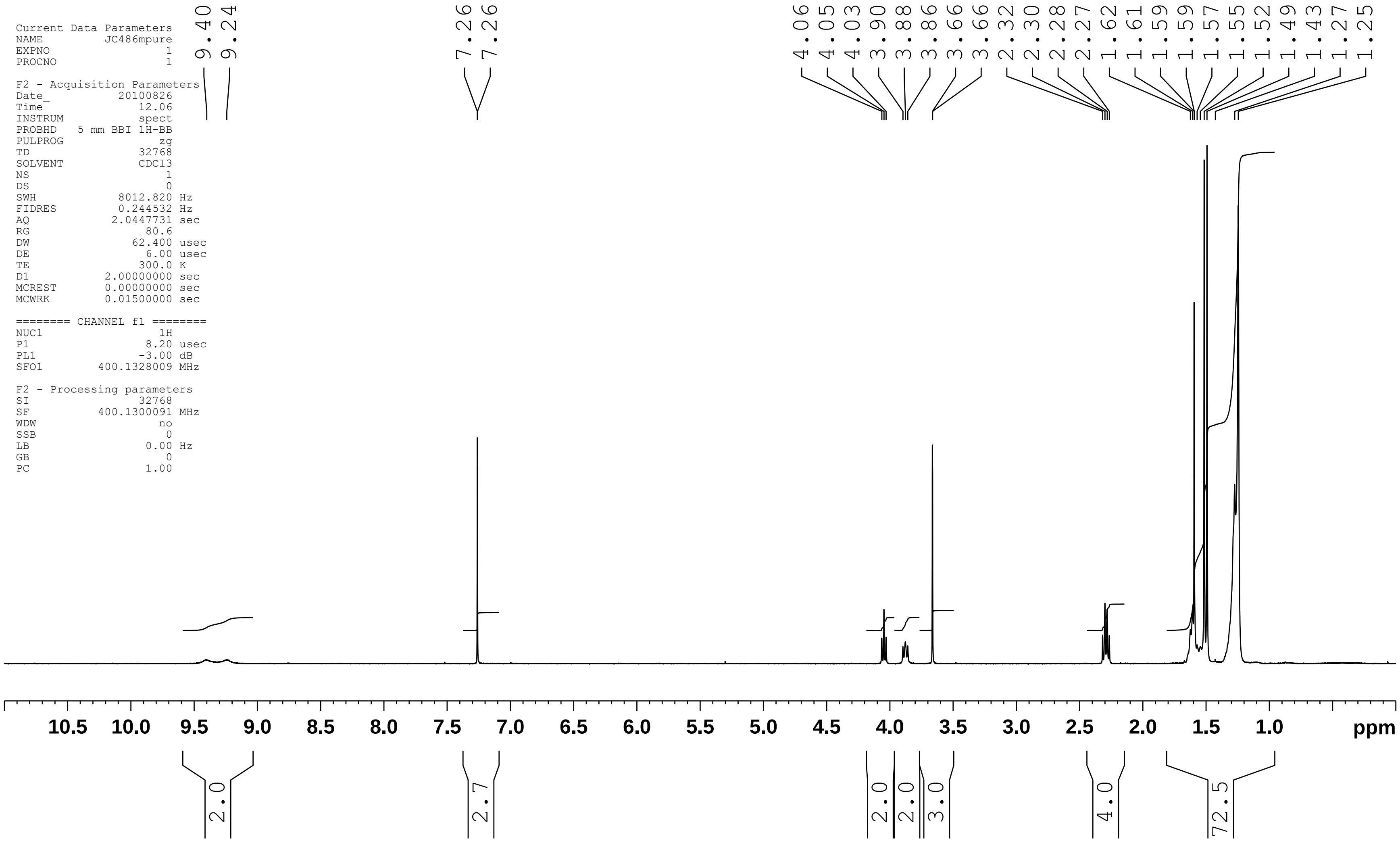

1-Methyl-15-[bis(*tert*-Butoxycarbonyl)guanidino] pentadecanoate (15)

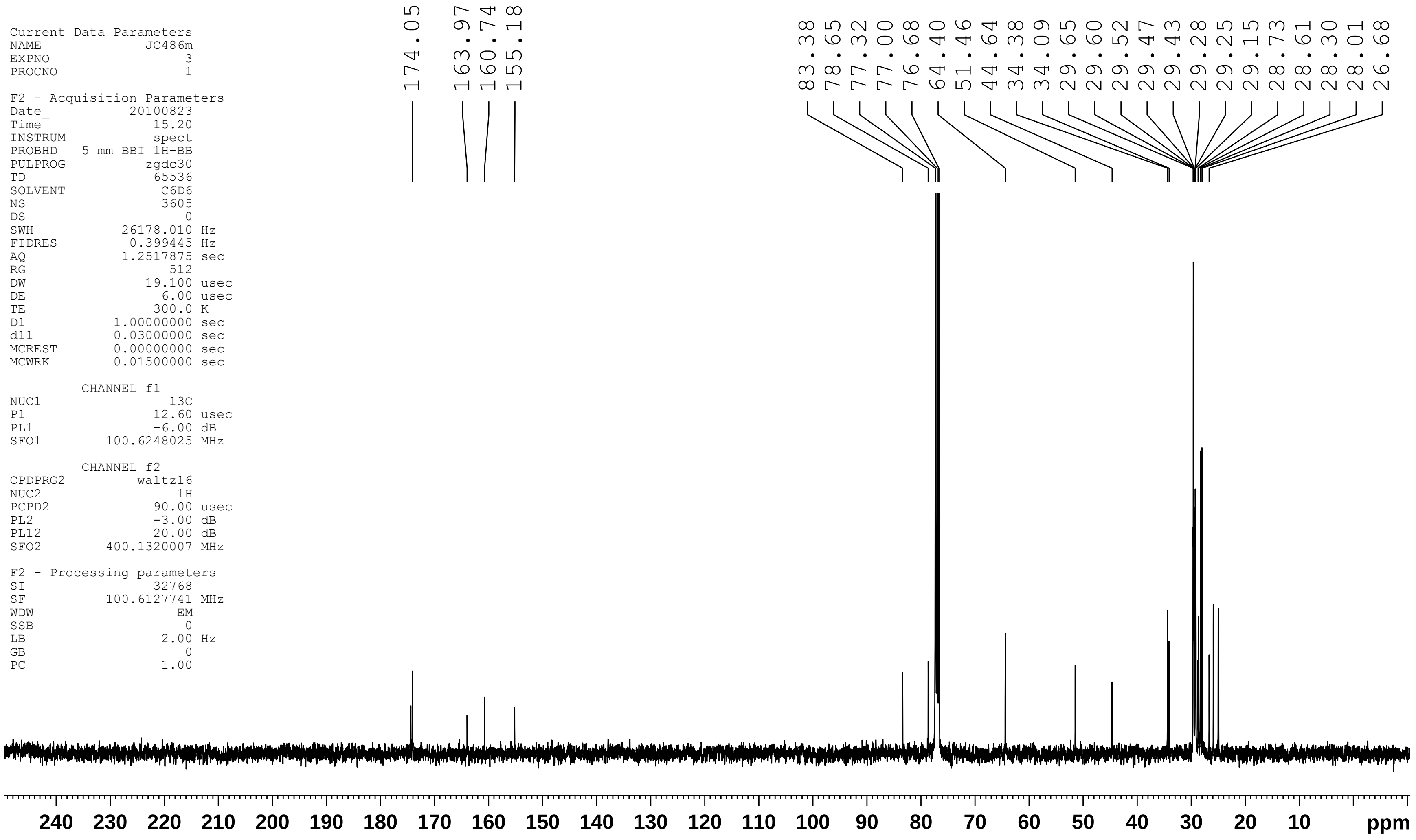

15-(*tert*-Butoxycarbonylguanidino) pentadecanoic acid (12)

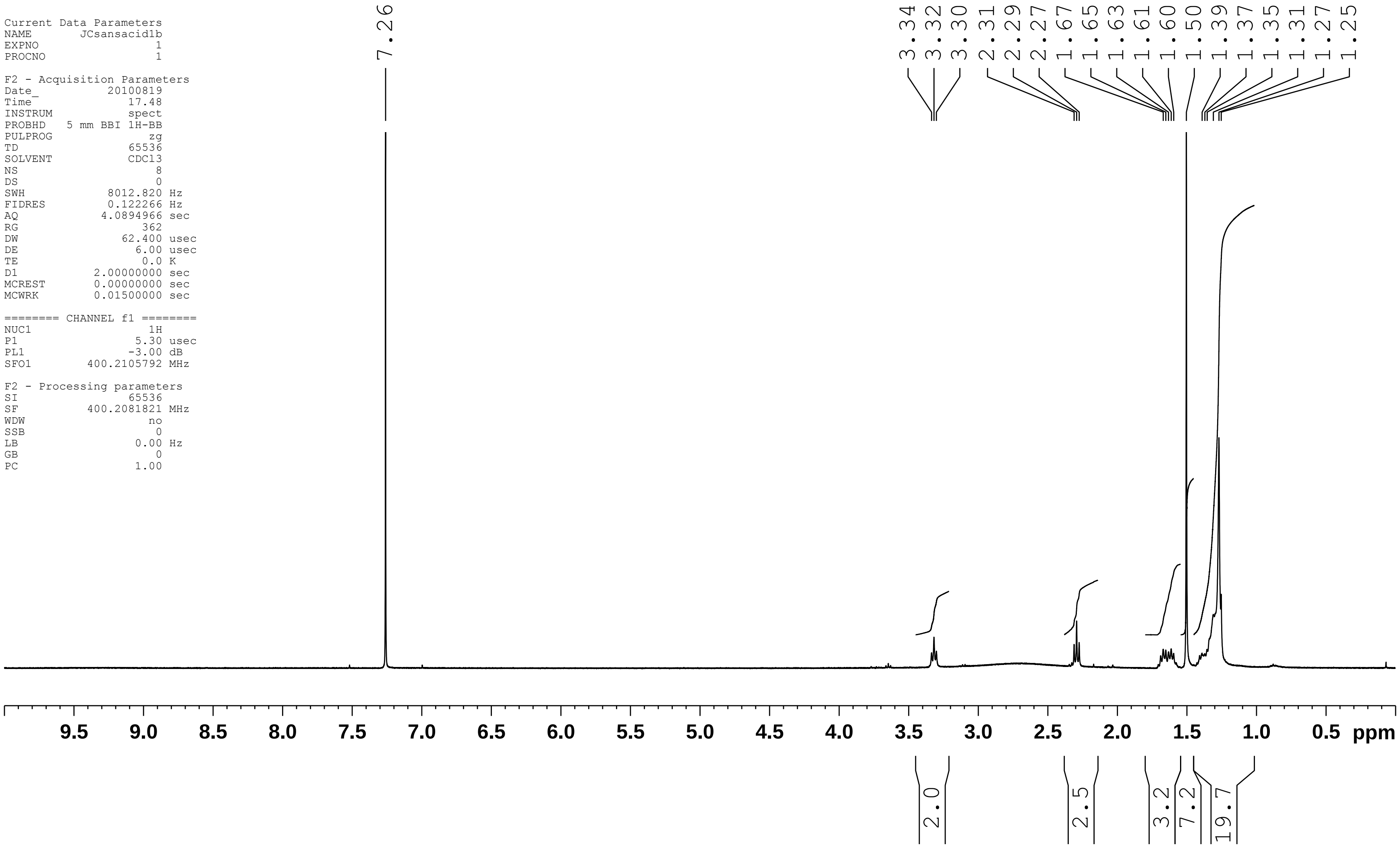

15-(tert-Butoxycarbonylguanidino) pentadecanoic acid (12)

```
Current Data Parameters
NAME     JCfinalacidsans3OH
EXPNO                 2
PROCNO                1

F2 - Acquisition Parameters
Date_          20100611
Time              17.59
INSTRUM           spect
PROBHD   5 mm BBI 1H-BB
PULPROG          zgdc30
TD                65536
SOLVENT            C6D6
NS                20480
DS                    0
SWH           26178.010 Hz
FIDRES         0.399445 Hz
AQ            1.2517875 sec
RG                 90.5
DW               19.100 usec
DE                 6.00 usec
TE                300.0 K
D1           1.00000000 sec
d11          0.03000000 sec
MCREST       0.00000000 sec
MCWRK        0.01500000 sec

======== CHANNEL f1 ========
NUC1                13C
P1                12.60 usec
PL1               -6.00 dB
SFO1        100.6248025 MHz

======== CHANNEL f2 ========
CPDPRG2         waltz16
NUC2                 1H
PCPD2             90.00 usec
PL2               -3.00 dB
PL12              20.00 dB
SFO2        400.1320007 MHz

F2 - Processing parameters
SI                32768
SF          100.6127709 MHz
WDW                  EM
SSB                   0
LB                 2.00 Hz
GB                    0
PC                 1.00
```

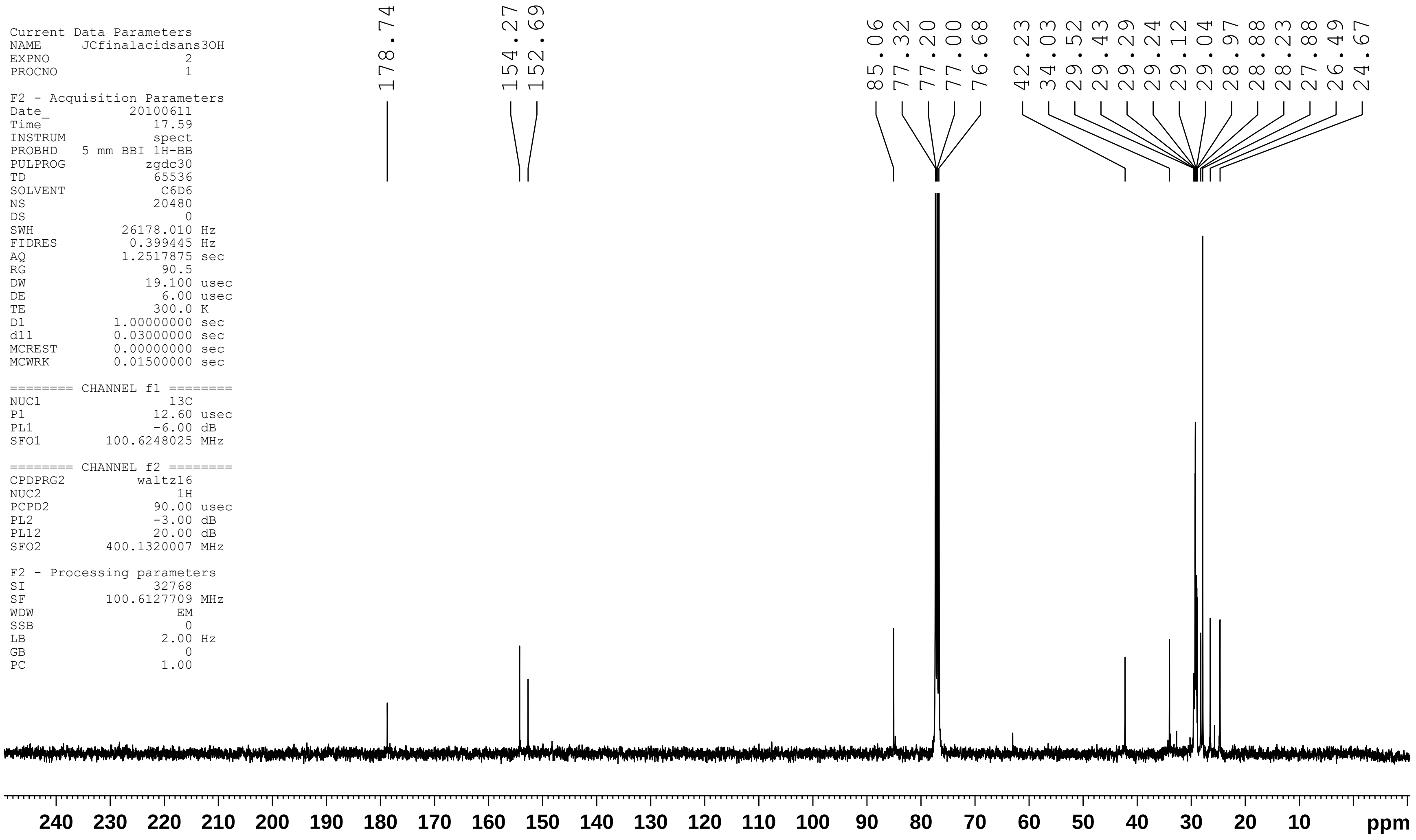

Dehydroxy LI-F04a analogue (21)

```
Current Data Parameters
NAME              JC497D
EXPNO                  1
PROCNO                 1

F2 - Acquisition Parameters
Date_           20100708
Time               19.31
INSTRUM            spect
PROBHD   5 mm BBI 1H-BB
PULPROG               zg
TD                 32768
SOLVENT            CDCl3
NS                    32
DS                     0
SWH             8012.820 Hz
FIDRES          0.244532 Hz
AQ             2.0447731 sec
RG                 161.3
DW                62.400 usec
DE                  6.00 usec
TE                 300.0 K
D1            2.00000000 sec
MCREST   0 sec
MCWRK         0.01500000 sec

======== CHANNEL f1 ========
NUC1                  1H
P1                  8.20 usec
PL1                -3.00 dB
SFO1         400.1328009 MHz

F2 - Processing parameters
SI                 32768
SF           400.1300029 MHz
WDW                   no
SSB      0
LB       0 Hz
GB       0
PC                  1.00
```

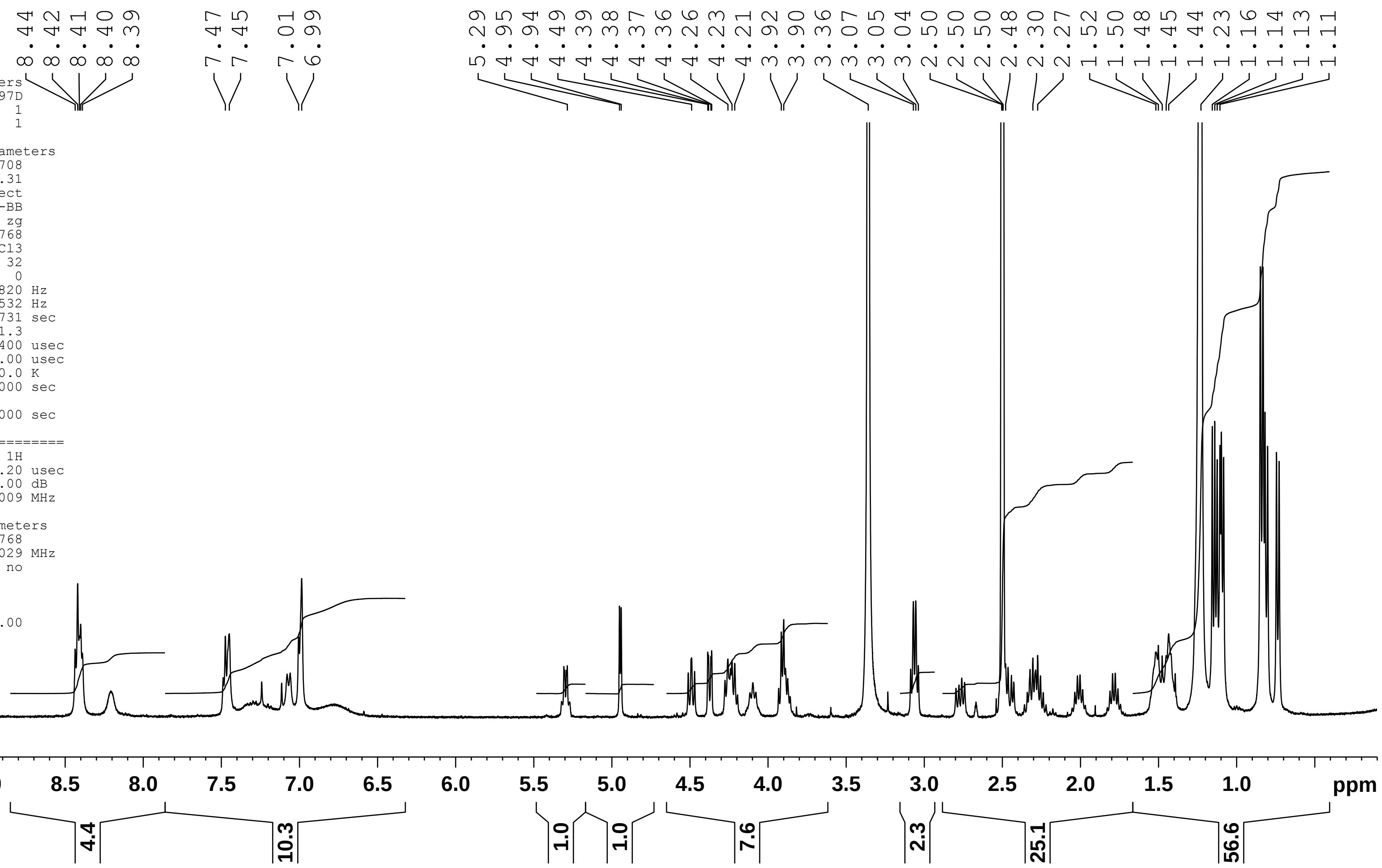

Dehydroxy LI-F04a analogue (21) COSY

```
Current Data Parameters
NAME              JC497D
EXPNO                  2
PROCNO                 1

F2 - Acquisition Parameters
Date_          20100708
Time              19.32
INSTRUM           spect
PROBHD   5 mm BBI 1H-BB
PULPROG        cosygpqf
TD                 2048
SOLVENT            MeOH
NS                   32
DS                    4
SWH            4006.410 Hz
FIDRES         1.956255 Hz
AQ            0.2556404 sec
RG              114.025
DW              124.800 usec
DE                 6.00 usec
TE                300.0 K
d0           0.00000300 sec
D1           1.10000002 sec
d13          0.00000400 sec
D16          0.00015000 sec
IN0          0.00024991 sec
MCREST   0 sec
MCWRK        1.10000002 sec

======== CHANNEL f1 ========
NUC1                 1H
P0                 4.10 usec
P1                 8.20 usec
PL1               -3.00 dB
SFO1        400.1320007 MHz

====== GRADIENT CHANNEL =====
GPNAM1         sine.100
GPNAM2         sine.100
GPX1     0 %
GPX2     0 %
GPY1     0 %
GPY2     0 %
GPZ1              10.00 %
GPZ2              10.00 %
P16             1500.00 usec

F1 - Acquisition parameters
TD                  512
SFO1            400.132 MHz
FIDRES         7.815235 Hz
SW               10.000 ppm
FnMODE               QF

F2 - Processing parameters
SI                 1024
SF          400.1300007 MHz
WDW               QSINE
SSB      0
LB       0 Hz
GB       0
PC                 1.00

F1 - Processing parameters
SI                  256
MC2                  QF
SF          400.1299929 MHz
WDW         States-TPPI
SSB      0
LB       0 Hz
GB       0
```

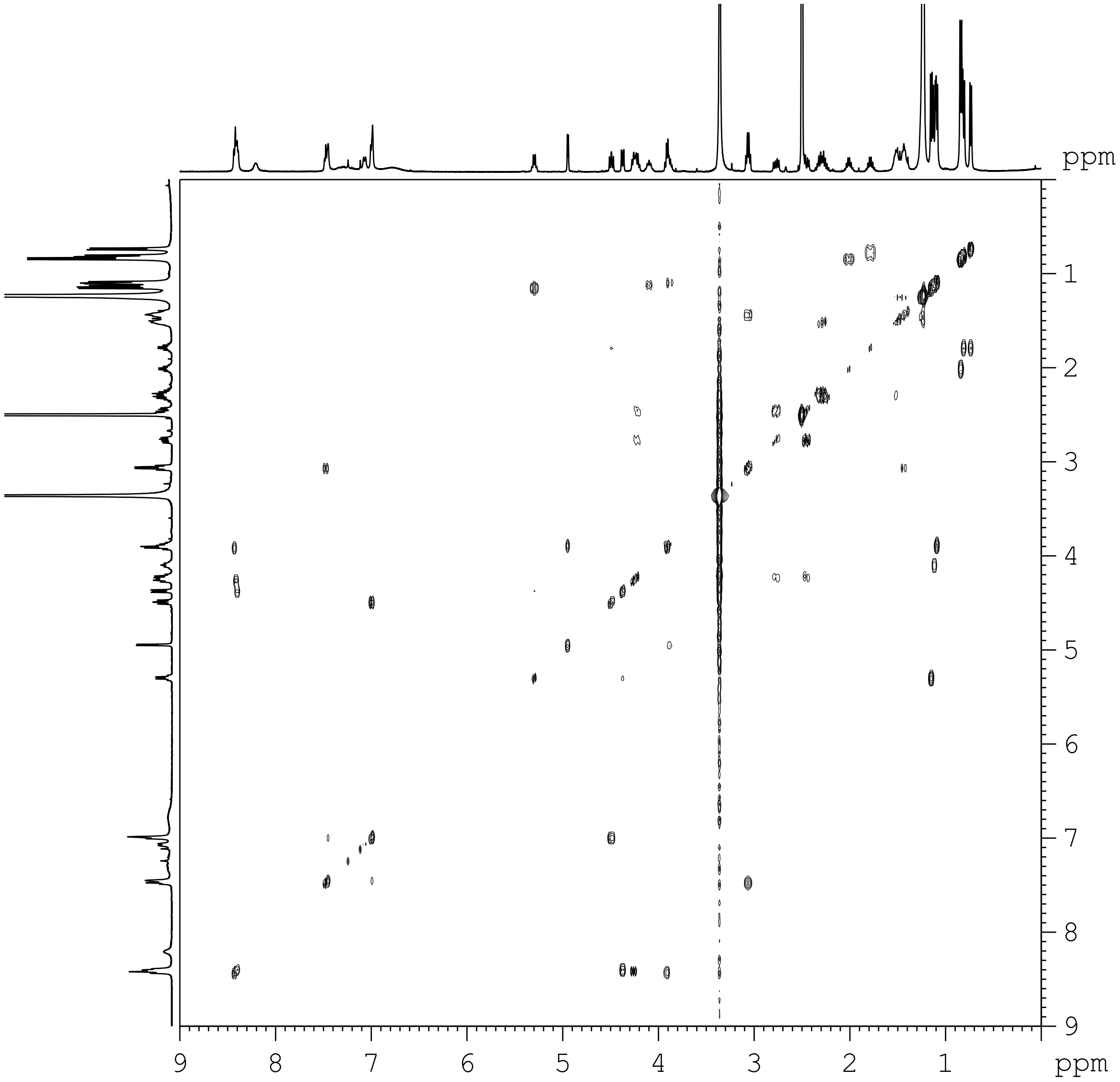

Dehydroxy LI-F04a analogue (21)

```
Current Data Parameters
NAME          JC497D13C
EXPNO                 2
PROCNO                1

F2 - Acquisition Parameters
Date_          20100903
Time              18.58
INSTRUM           spect
PROBHD   5 mm DUL 13C-1
PULPROG         zgpg30
TD               65536
SOLVENT          CDCl3
NS               98550
DS                   2
SWH          26178.010 Hz
FIDRES        0.399445 Hz
AQ           1.2517875 sec
RG                90.5
DW              19.100 usec
DE                6.00 usec
TE       0 K
D1          1.00000000 sec
d11         0.03000000 sec
DELTA       0.89999998 sec
MCREST   0 sec
MCWRK       0.01500000 sec

======== CHANNEL f1 ========
NUC1                13C
P1                 5.40 usec
PL1               -6.00 dB
SFO1        100.6445019 MHz

======== CHANNEL f2 ========
CPDPRG2         waltz16
NUC2                 1H
PCPD2             80.00 usec
PL2               -3.00 dB
PL12              13.92 dB
PL13              20.00 dB
SFO2        400.2101790 MHz

F2 - Processing parameters
SI                65536
SF          100.6324773 MHz
WDW                  EM
SSB      0
LB                 3.00 Hz
GB       0
PC                 1.00
```

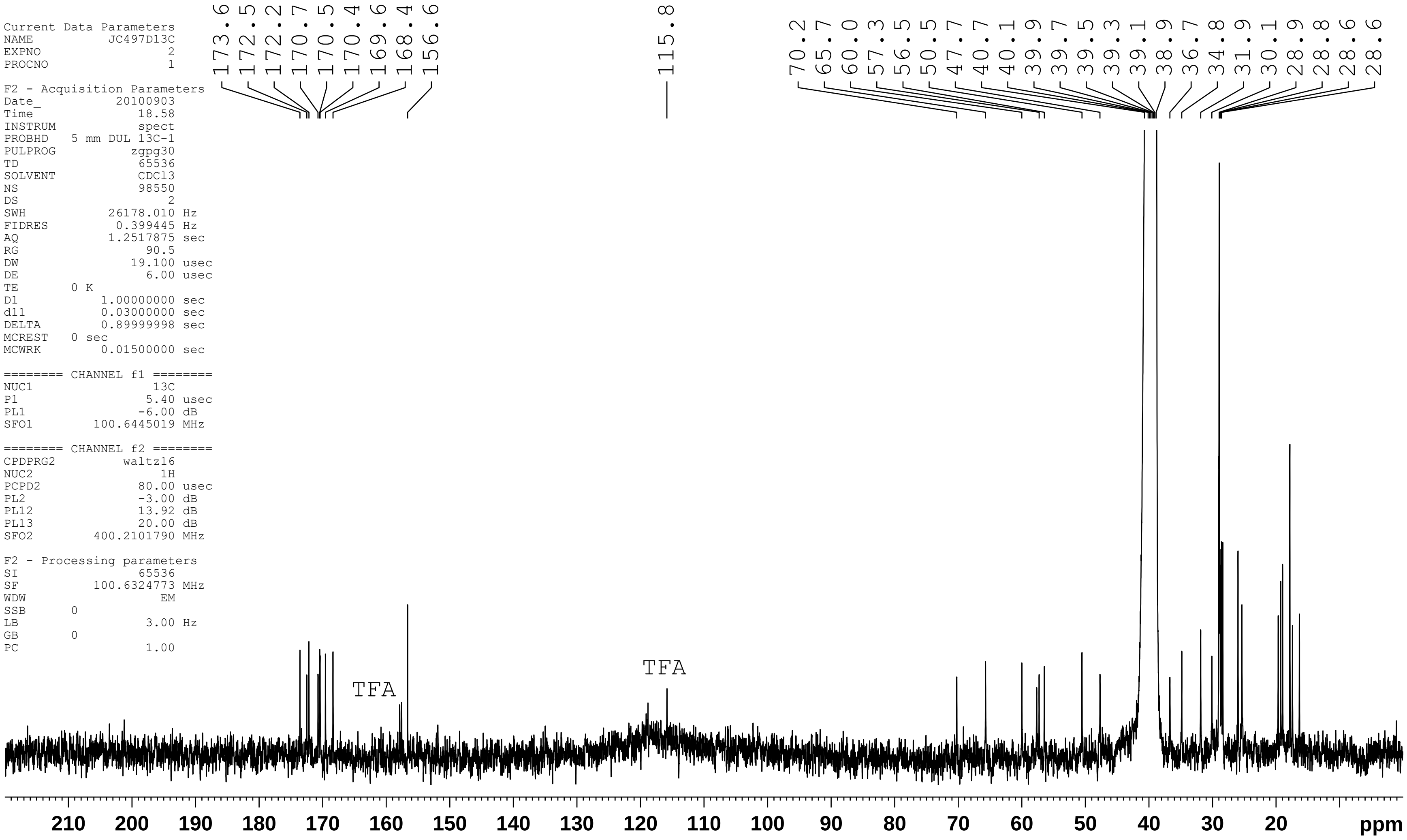

Dehydroxy LI-F04a analogue (21) HSQC

```
Current Data Parameters
NAME              JC497D
EXPNO                  7
PROCNO                 1

F2 - Acquisition Parameters
Date_           20100725
Time               12.54
INSTRUM            spect
PROBHD   5 mm BBI 1H-BB
PULPROG       invietgpsi
TD                  2048
SOLVENT             DMSO
NS                    64
DS                     8
SWH             4006.410 Hz
FIDRES          1.956255 Hz
AQ             0.2556404 sec
RG                   512
DW               124.800 usec
DE                  6.00 usec
TE                 309.9 K
CNST2        140.0000000
d0            0.00000300 sec
D1            1.50000000 sec
d4            0.00178571 sec
d11           0.03000000 sec
d13           0.00000400 sec
D16           0.00015000 sec
D24           0.00178500 sec
DELTA         0.00172240 sec
DELTA1        0.00165800 sec
IN0           0.00004969 sec
MCREST   0 sec
MCWRK         0.25000051 sec
ST1CNT                 0

======== CHANNEL f1 ========
NUC1                  1H
P1                  8.20 usec
p2                 16.40 usec
P28                 0.50 usec
PL1                -3.00 dB
SFO1         400.1320007 MHz

======== CHANNEL f2 ========
CPDPRG2             garp
NUC2                 13C
P3                 12.00 usec
p4                 24.00 usec
PCPD2              70.00 usec
PL2                -6.00 dB
PL12               10.70 dB
SFO2         100.6177596 MHz

====== GRADIENT CHANNEL =====
GPNAM1          sine.100
GPNAM2          sine.100
GPX1      0 %
GPX2      0 %
GPY1      0 %
GPY2      0 %
GPZ1               80.00 %
GPZ2               20.10 %
P16              1500.00 usec

F1 - Acquisition parameters
TD                   512
SFO1            100.6178 MHz
FIDRES         19.654089 Hz
SW               100.011 ppm
FnMODE    Echo-Antiecho

F2 - Processing parameters
SI                  1024
SF           400.1299973 MHz
WDW                 SINE
SSB                    2
LB        0 Hz
GB        0
PC                  1.40

F1 - Processing parameters
SI                  1024
MC2       echo-antiecho
SF           100.6128435 MHz
WDW
SSB                    2
LB        0 Hz
GB        0
```

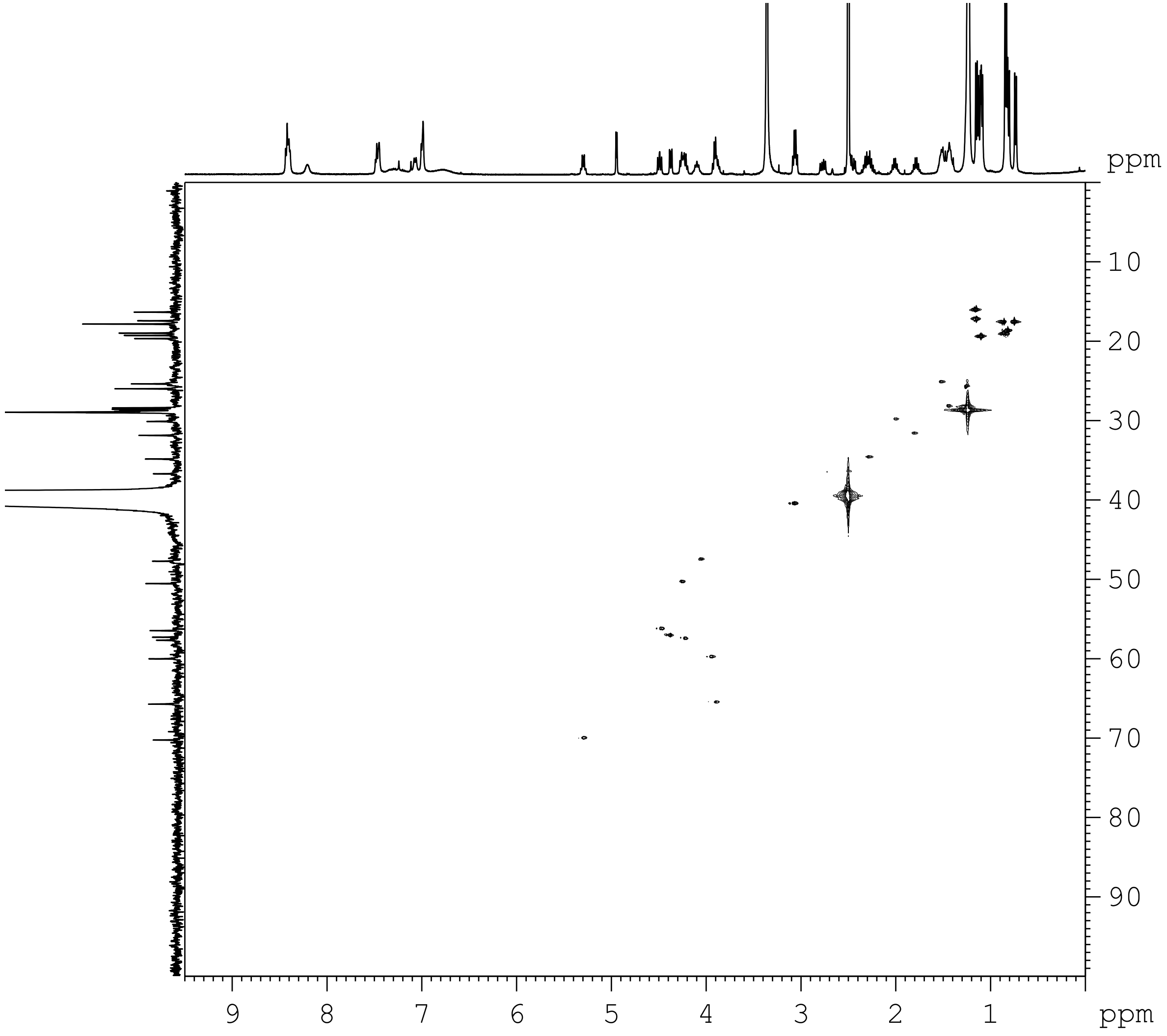

Dehydroxy LI-F04a analogue (21) HMBC

```
Current Data Parameters
NAME              JC497D
EXPNO                  7
PROCNO                 1

F2 - Acquisition Parameters
Date_           20100725
Time               12.54
INSTRUM            spect
PROBHD   5 mm BBI 1H-BB
PULPROG      invietgpsi
TD                  2048
SOLVENT             DMSO
NS                    64
DS                     8
SWH             4006.410 Hz
FIDRES          1.956255 Hz
AQ             0.2556404 sec
RG                   512
DW               124.800 usec
DE                  6.00 usec
TE                 309.9 K
CNST2        140.0000000
d0            0.00000300 sec
D1            1.50000000 sec
d4            0.00178571 sec
d11           0.03000000 sec
d13           0.00000400 sec
D16           0.00015000 sec
D24           0.00178500 sec
DELTA         0.00172240 sec
DELTA1        0.00165800 sec
IN0           0.00004969 sec
MCREST   0 sec
MCWRK         0.25000051 sec
ST1CNT                 0

======== CHANNEL f1 ========
NUC1                  1H
P1                  8.20 usec
p2                 16.40 usec
P28                 0.50 usec
PL1                -3.00 dB
SFO1         400.1320007 MHz

======== CHANNEL f2 ========
CPDPRG2             garp
NUC2                 13C
P3                 12.00 usec
p4                 24.00 usec
PCPD2              70.00 usec
PL2                -6.00 dB
PL12               10.70 dB
SFO2         100.6177596 MHz

====== GRADIENT CHANNEL =====
GPNAM1          sine.100
GPNAM2          sine.100
GPX1     0 %
GPX2     0 %
GPY1     0 %
GPY2     0 %
GPZ1               80.00 %
GPZ2               20.10 %
P16              1500.00 usec

F1 - Acquisition parameters
TD                   512
SFO1            100.6178 MHz
FIDRES         19.654089 Hz
SW               100.011 ppm
FnMODE    Echo-Antiecho

F2 - Processing parameters
SI                  1024
SF           400.1299973 MHz
WDW                 SINE
SSB                    2
LB       0 Hz
GB       0
PC                  1.40

F1 - Processing parameters
SI                  1024
MC2        echo-antiecho
SF           100.6128435 MHz
WDW
SSB                    2
LB       0 Hz
GB       0
```

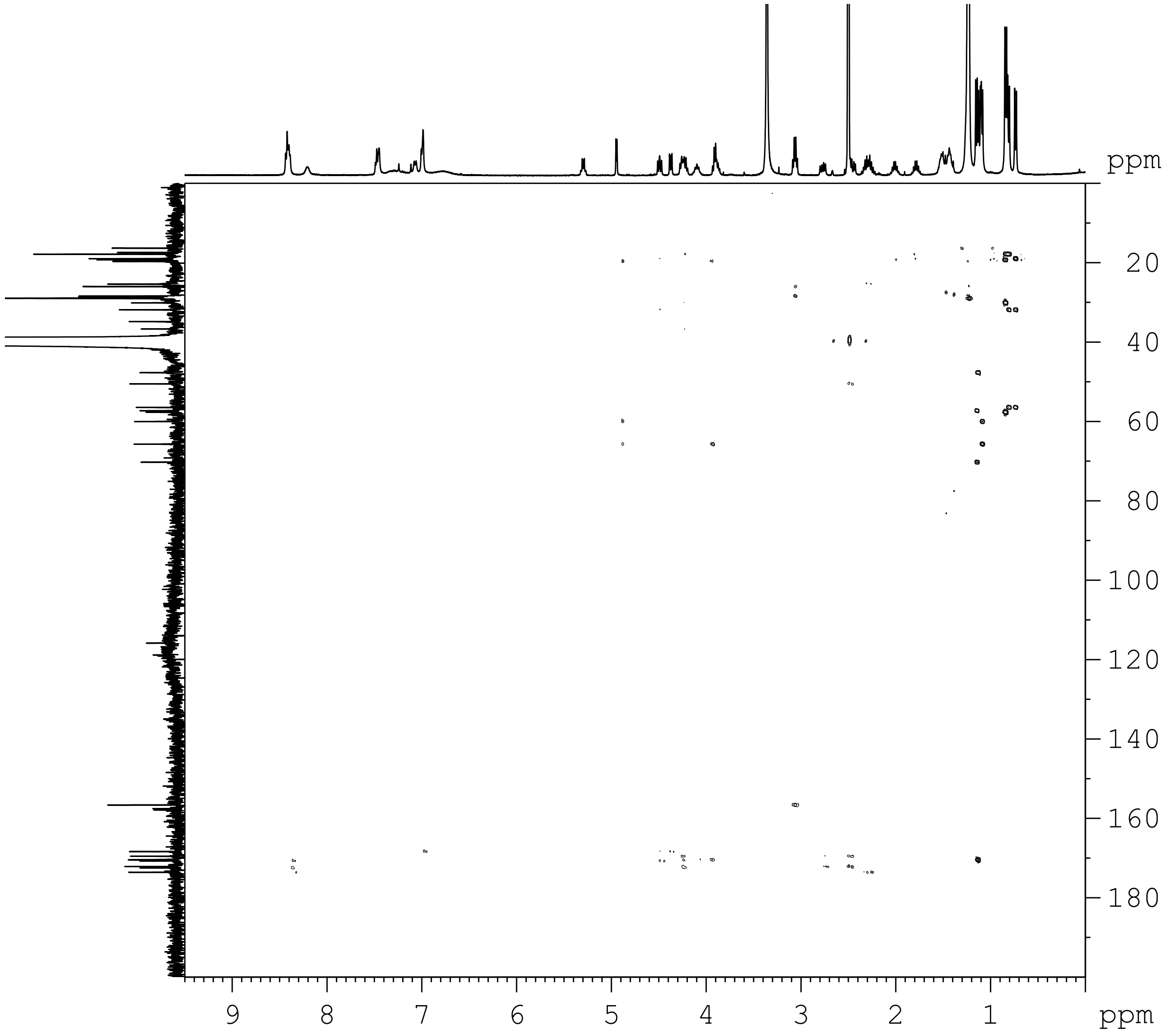

Supplement: File 2 — 1H, 13C and 2D NMR data for all new compounds. [file Beilstein_J_Org_Chem-08-1344-s002.pdf]
